# Copper starvation induces antimicrobial isocyanide integrated into two distinct biosynthetic pathways in fungi

**DOI:** 10.1038/s41467-022-32394-x

**Published:** 2022-08-16

**Authors:** Tae Hyung Won, Jin Woo Bok, Nischala Nadig, Nandhitha Venkatesh, Grant Nickles, Claudio Greco, Fang Yun Lim, Jennifer B. González, B. Gillian Turgeon, Nancy P. Keller, Frank C. Schroeder

**Affiliations:** 1 Boyce Thompson Institute and Department of Chemistry and Chemical Biology, Cornell University, Ithaca, NY USA; 2 Department of Medical Microbiology and Immunology, University of Wisconsin-Madison, Madison, WI USA; 3 Department of Bacteriology, University of Wisconsin-Madison, Madison, WI USA; 4 Department of Plant Pathology, University of Wisconsin-Madison, Madison, WI USA; 5 Department of Cellular and Molecular Biology, University of Wisconsin-Madison, Madison, WI USA; 6 Department of Plant Pathology and Plant-Microbe Biology, Cornell University, Ithaca, NY USA; 7 Present Address: Department of Molecular Microbiology, John Innes Centre, Norwich, NR4 7UH United Kingdom; 8 Present Address: Department of Chemistry, University of Washington, Seattle, WA USA; 9 Present Address: 104 Peckham Hall, Nazareth College, 4245 East Avenue, Rochester, NY USA

**Keywords:** Metabolomics, Fungal ecology, Biosynthesis, Antimicrobials

## Abstract

The genomes of many filamentous fungi, such as *Aspergillus* spp., include diverse biosynthetic gene clusters of unknown function. We previously showed that low copper levels upregulate a gene cluster that includes *crmA*, encoding a putative isocyanide synthase. Here we show, using untargeted comparative metabolomics, that CrmA generates a valine-derived isocyanide that contributes to two distinct biosynthetic pathways under copper-limiting conditions. Reaction of the isocyanide with an ergot alkaloid precursor results in carbon-carbon bond formation analogous to Strecker amino-acid synthesis, producing a group of alkaloids we term fumivalines. In addition, valine isocyanide contributes to biosynthesis of a family of acylated sugar alcohols, the fumicicolins, which are related to brassicicolin A, a known isocyanide from *Alternaria brassicicola*. CrmA homologs are found in a wide range of pathogenic and non-pathogenic fungi, some of which produce fumicicolin and fumivaline. Extracts from *A. fumigatus* wild type (but not *crmA*-deleted strains), grown under copper starvation, inhibit growth of diverse bacteria and fungi, and synthetic valine isocyanide shows antibacterial activity. CrmA thus contributes to two biosynthetic pathways downstream of trace-metal sensing.

## Abstract (web summary)

The genomes of filamentous fungi, such as *Aspergillus*, include many biosynthetic gene clusters of unknown function. Here, the authors show that copper starvation induces expression of an enzyme that generates a valine-derived isocyanide participating in two different pathways, for biosynthesis of acylated sugar alcohols and modified ergot alkaloids.

## Introduction

The Kingdom Fungi produces an enigmatic diversity of secondary metabolites derived from dedicated biosynthetic gene clusters (BGCs) that encode the enzymes required to convert common primary metabolic building blocks into secondary metabolites. Genomic sequencing of 1000 s of fungi has revealed a vast space of BGCs with as yet undetermined function; however, their distribution among taxa is uneven: whereas yeasts contain few, if any, BGCs, some filamentous fungi such as *Aspergillus* spp. may have upwards of 70 BGCs/genome^1^. This tremendous expansion of BGCs in specific taxa remains an evolutionary enigma. Experimental evidence for functional roles of a few secondary metabolites (such as polyketide pigment protection of fungal spores from UV light^2^ or antibacterial properties of fungal secondary metabolites induced in fungal/bacterial culture^2^) supports the hypothesis that BGC-encoded metabolites are produced to address specific environmental constraints or challenges.

We became intrigued with the potential of fungal BGCs evolved to fit specific environmental niches upon our discovery of a new family of secondary metabolite biosynthetic enzymes, the isocyanide synthases (ICSs), in the filamentous pathogen *Aspergillus fumigatus*^3^. Curiously we found that one ICS-BGC was regulated by MacA (also called Mac1), a copper fist transcription factor that functions to activate copper importers during copper starvation^4^. This BGC, termed the *crm* (*c*opper *r*esponsive *m*etabolite) BGC, was activated in defined media lacking the essential trace element copper in a MacA-dependent manner^3^. Copper homeostasis is carefully controlled in both prokaryotes and eukaryotes, as many biological processes require copper ions, yet too much copper is toxic.

Because loss of MacA and/or its importers greatly impair *A. fumigatus* growth in copper-limited conditions^4,5^, we wondered if the *crm* BGC was involved in fungal biology under low copper environments. We found that, under copper starvation conditions, CrmA converts l-valine to (*S*)−2-isocyanoisovaleric acid, which is then used for two different biosynthetic pathways. The isocyanide, in an unusual chemical reaction, attaches to chanoclavine-1 aldehyde (an ergot alkaloid biosynthesis intermediate)^6,7^ to form a new family of alkaloid peptides named the fumivalines. The formation of the fumivalines involves a carbon-carbon bond-forming reaction that is analogous to a well-known synthetic reaction, the Strecker amino acid synthesis. *Penicillium* spp., which contains a homolog of the *crm* BGC as well as an intact ergot alkaloid pathway, also produce fumivaline, which is, as in *A. fumigatus*, induced by copper starvation. Alternatively, the valine-derived isocyanoisovaleric acid is employed for the synthesis of acylated d-mannitol derivatives that represent plausible intermediates toward the biosynthesis of brassicicolin A, an isocyanide previously identified from *Alternaria brassicicola*^8^. Another ascomycete, *Cochliobolus heterostrophus*, whose genome includes a *crmA* homolog but lacks the ergot alkaloid pathway, produces related acylated d-mannitol derivatives under copper starvation conditions, but lacks the fumivalines.

Here we show that *A. fumigatus* employs an ICS to produce a reactive building block that is then integrated into two different secondary metabolite families via distinct chemical reactions, one of which provides the first example for Strecker-like amino acid biosynthesis in eukaryotes^9^. Copper starvation dependence of the identified metabolites and their antibacterial effects suggests that CrmA-dependent pathways provide fungi with niche-specific weaponry to increase fitness under environmental stress.

## Results

### CrmA encodes a multidomain ICS-NRPS-like enzyme

**Fig. 1:**
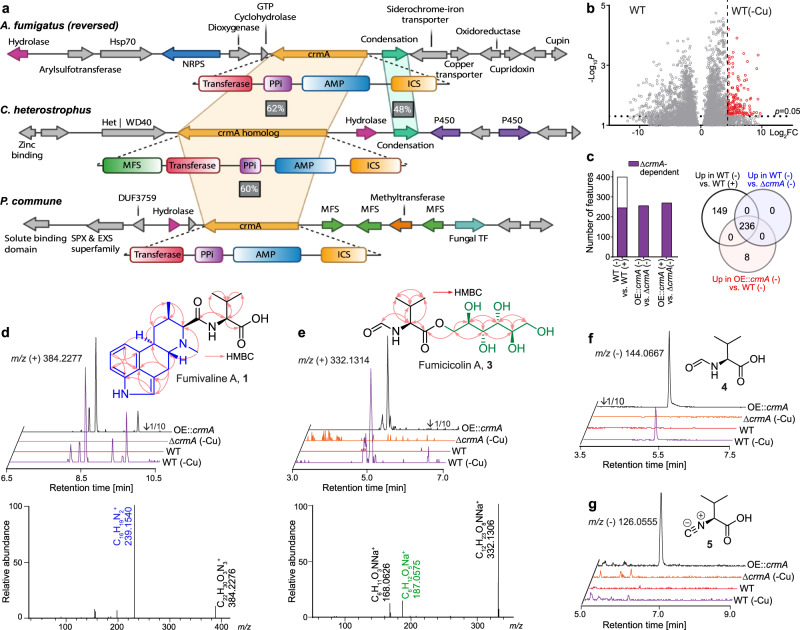
The *crm* BGC and associated metabolites from *A. fumigatus*. **a** Comparison of *crm* BGCs in *A. fumigatus*, *P. commune*, and *C. heterostrophus*. Homologous genes are marked with colors based on their corresponding functions. **b** Volcano plot of differential metabolites in wild type upregulated by copper starvation (red dots represent metabolites 20-fold upregulated at *p* < 0.05 as calculated by unpaired two-sided *t*-test, unadjusted for the number of comparisons, based on 6 independent replicates). **c** Total number of *crmA*-dependent metabolites in wildtype upregulated by copper starvation or *crmA* overexpression compared to the *crmA* deletion mutant and Venn diagram showing overlap of metabolites induced in WT *A. fumigatus* under copper-limited relative to copper-replete conditions, metabolites abolished in *crmA* deletion mutants, and metabolites induced in *crmA* overexpression mutants relative to WT grown under copper-limited conditions. **d**, **e** Structures, MS2 spectra, ESI + ion chromatograms, and HMBC correlations of fumivaline A (**1**) and fumicicolin A (**3**). Part of the structure highlighted in blue represents festuclavine (**2**). **f**, **g** Structures and ESI- ion chromatograms for *N-*formylvaline (**4**) and (*S*)−2-isocyanoisovaleric acid (valine isocyanide, **5**). Source data are provided as a Source Data file.

Comparative analyses of available fungal genomes revealed the multidomain ICS-NRPS-like gene *crmA* encoding adenylation, thiolation, and transferase domains, in addition to the isocyanide synthase domain (Fig. 1a)^3^. CrmA homologs are present primarily in three diverse ascomycete classes: Eurotiomycetes (e.g., *Aspergillus, Penicillium, Talaromyces, Trichophyton* spp.), Dothideomycetes (e.g., *Cochliobolus, Leptosphaeria, Alternaria* spp.) and Sordariomycetes (e.g., *Verticillium, Metarhizium, Fusarium, Cordyceps, Beauveria, Trichoderma* spp.) with the majority of genera consisting of pathogenic fungi. Variations in domain structure include the addition of a transporter domain found primarily in the Dothideomycetes, a clade that contains common leaf blight/spot fungal pathogens (Fig. 1a, Supplementary Fig. 1, and Supplementary Data 1). In *A. fumigatus*, CrmA is part of the four-gene *Crm* cluster which additionally includes a predicted alcohol-*O*-acetyl transferase (*crmB*), siderochrome-iron transporter (*crmC*), and CtrA1 copper transporter (*crmD*), all of which are co-regulated by copper (Fig. 1a)^3^. To our surprise, we found little conservation *crmB*, *C*, and *D* in more distantly related species.

### Identification of *crm* cluster-derived metabolites

To identify *crm*-derived metabolites, we started by comparing the metabolomes of wildtype (WT) *A. fumigatus* and a Δ*crmA* deletion mutant grown under copper-limited and copper-replete conditions, given that *crmA* expression is strongly copper-dependent^3^. Metabolite extracts were analyzed by high-resolution HPLC-MS (HPLC-HRMS), and the resulting datasets were compared using the *Metaboseek* software platform^10^, which facilitates comparative analysis of HPLC-HRMS data from multiple different conditions. These comparative analyses revealed a large number of MS features upregulated under low copper conditions. For example, we detected 385 features that were 20-fold or more upregulated under copper-limited conditions (*p* < 0.05) among a total of 30,000 detected features from both positive and negative ionization modes (Fig. 1b, see Methods for details on data processing). Production of 236 features induced under copper-limited conditions, representing ~30 distinct metabolites, was abolished in the Δ*crmA* mutant, and in fact all features abolished in the Δ*crmA* mutant were also copper-dependent (Fig. 1c). Next, we examined the metabolome of a *crmA* overexpression mutant (OE::*crmA*) under copper-limited and copper-replete conditions. Production of most metabolites abolished in the Δ*crmA* mutant was increased above wild-type levels in OE::*crmA* regardless of a copper regime (Fig. 1c), further supporting that they represent CrmA-derived metabolites. Interestingly, none of the *crmA-*dependent features were affected by the deletion of any of the other cluster genes, *crmB-D* (Supplementary Data 2).

The *crmA-* and copper-dependent MS features were further characterized via tandem MS (MS2) and MS2 networking, which revealed several families of structurally related *crmA*-dependent metabolites. We then selected the most abundant family members for partial purification via reversed-phase preparative HPLC for NMR spectroscopic analysis (Fig. 1d–g, Supplementary Fig. 2a–e, see Supplementary Information for NMR data). This analysis revealed two distinct families of metabolites, both of which integrate an *N*-formylvaline moiety. Fumivaline A (**1**) (Fig. 1d) and the hydroxylated derivative fumivaline B (Supplementary Fig. 2a) represent modified ergot alkaloids featuring a festuclavine (**2**)-like ring system, whereas fumicicolin A (**3**) (Fig. 1e) and fumicicolin B (Supplementary Fig. 2b) represent esters of *N*-formylvaline with D-mannitol or a hexose, respectively. In addition, we detected free *N*-formylvaline (**4**) (Fig. 1f and Supplementary Fig. 2c), as well as small amounts of (*S*)−2-isocyanoisovaleric acid (**5**) (Fig. 1g and Supplementary Fig. 2d, e), a plausible precursor of the identified *N-*formylvaline derivatives (Supplementary Table 1). The identities of fumicicolin A (**3**) and (*S*)−2-isocyanoisovaleric acid (valine isocyanide, **5**) were further confirmed using synthetic standards prepared from commercially available *N*-formylvaline (Supplementary Fig. 2f). Detailed descriptions of the syntheses are provided in the Supplementary Information. The (*S*)-configuration of the α-carbon in the valine-like moieties in fumivaline A and *N*-formylvaline was established using Marfey’s method^11^ (Supplementary Fig. 2g, h), suggesting that the *crmA*-derived metabolites are derived from l-valine. Lastly, we showed that the relative configuration of fumivaline A is identical to that of the related ergot alkaloid festuclavine (**2**) using NOESY spectra (Supplementary Fig. 2i)^7,12^.

### Fumivaline biosynthesis requires the ergot alkaloid BGC

**Fig. 2:**
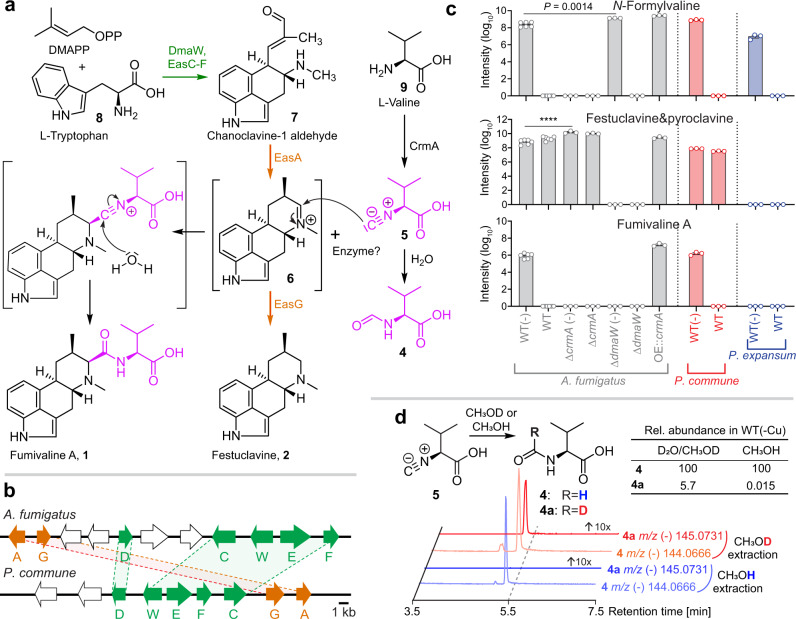
Comparison of fumivaline BGCs and ergot alkaloids production. **a** Ergot alkaloid biosynthesis pathways in *A. fumigatus* and *P. commune*. l-Tryptophan (**8**) and dimethylallyl pyrophosphate are converted into chanoclavine-1 aldehyde (**7**) by DmaW and EasC-F, which is then converted into festuclavine (**2**) by EasA and EasG in *A. fumigatus* and *P. commune*. Valine (**9**) is converted into valine isocyanide (**5**) by CrmA, which then reacts with the imine intermediate of festuclavine (**6**), resulting in formation of the amide bond in fumivaline A following hydration. In addition, hydration of valine isocyanide (**5**) produces *N*-formylvaline (**4**). **b** Comparison of *crm* BGCs of *A. fumigatus* and *P. commune*. Homologous genes are marked using the same colors as in **a**. A and C-G represent *easA* and *easC-G* respectively. W represents *dmaW*. **c** Relative abundances of *N*-formylvaline (**4**), fumivaline A (**1**), and festuclavine (**2**) and pyroclavine in *A. fumigatus*, *P. commune*, and *P. expansum* (gray, red, and blue, respectively) grown without copper (-) or with copper. Bars represent mean ± s.e.m. with six independent biological replicates for *A. fumigatus* wildtype and three for the other strains. *p* values were calculated by unpaired, two-sided *t*-test with Welch’s correction, ****P < 0.0001. **d** Relative abundances of *N-*formylvaline (**4**) and [7-^2^H]-*N-*formylvaline (**4a**) in extracts of wildtype *A. fumigatus* (grown without copper) extracted with deuterated or non-deuterated solvents. Source data are provided as a Source Data file.

Ergot alkaloids are a class of tryptophan-derived alkaloids featuring the characteristic ergoline tetracyclic ring structure (Fig. 2a and Supplementary Fig. 3a)^12,13^. Given that the structures of the fumivalines are related to that of the ergot alkaloid festuclavine, it appeared that *crmA* worked in conjunction with the ergot alkaloid BGC for fumivaline biosynthesis. To confirm the requirement of the ergot alkaloid BGC in the biosynthesis of fumivalines, we examined the metabolome of a deletion mutant of DmaW, a rate-limiting enzyme involved in the condensation reaction of tryptophan and dimethylallyl pyrophosphate (DMAPP) in ergot alkaloid BGCs^7,14,15^, in *A. fumigatus* (Fig. 2a, b). Deletion of *dmaW* eliminated the production of fumivalines A and B as well as of festuclavine and pyroclavine, whereas the abundance of other *crm*A-derived metabolites, including *N*-formylvaline remained unchanged or increased (Fig. 2c and Supplementary Fig. 3b). In turn, deletion of *crmA* eliminated *N*-formylvaline, fumivalines A and B synthesis but slightly increased the production of festuclavine and pyroclavine (Fig. 2c and Supplementary Fig. 3b). These data confirm that both *crmA* and the ergot alkaloid BGC are required for the production of the fumivalines.

### Strecker-like peptide formation in fumivaline biosynthesis

Known ergot alkaloids can be divided into three classes, ergoclavines, ergoamides, and ergopeptines, based on their substituents at C-8 position (Supplementary Fig. 3a). d-lysergic acid and its amides are ergoamides derived from the oxidation of a methyl group at the C-8 position of ergoclavines. Ergopeptines^12,13^ are peptides of d-lysergic acid showing complex structures linked with a peptide bond at the C-8 position synthesized via a canonical NRPS pathway. In contrast, fumivalines identified in this study represent a new class of ergot alkaloids that contains an unprecedented peptide moiety attached to the C-7 position.

Fumivaline A biosynthesis could plausibly result from a nucleophilic attack of valine-derived isocyanide from CrmA on an imine intermediate of the ergot alkaloid biosynthesis (Fig. 2a, b). Therefore, we hypothesized that CrmA first generates valine isocyanide (**5**), which is then attached to a previously proposed precursor of festuclavine, dehydrofestuclavine (**6**), via nucleophilic attack of the isocyanide carbon of **5** at the imine carbon of **6**. The formation of the carbon-carbon bond is then followed by hydration, resulting in the amide bond in position C-7 of the fumivalines (Fig. 2a). This mechanism is closely related to the well-known Strecker synthesis of amino acids and peptides from isocyanides and imine intermediates (Supplementary Fig. 3c).

The Strecker reactions usually yield racemic mixtures of α-amino acids as products. Correspondingly, our HPLC-HRMS analyses revealed multiple isomers of fumivalines A and B with identical MS2 spectra, likely representing diastereomers (Fig. 1d and Supplementary Fig. 2a), consistent with non-stereoselective peptide bond formation in fumivaline biosynthesis. Therefore, we asked whether fumivaline formation could have resulted from the reaction of valine isocyanide with festuclavine biosynthesis intermediates, e.g., **6**, during the processing or extraction of the fungal cultures. Although we did not detect valine isocyanide in WT *A. fumigatus* and the amounts of valine isocyanide found in our analyses of the *crmA* overexpression mutant are small compared to the amounts of the other *crmA*-derived metabolites (Fig. 2c and Supplementary Fig. 2e), it seemed possible that most of the initially produced valine isocyanide could have converted into *N-*formylvaline during extraction. In fact, we found that synthetic valine isocyanide is rapidly hydrated to form *N*-formylvaline under acidic conditions, e.g., during HPLC analysis. To test whether the *N*-formylvaline in our fungal extracts was derived from the hydration of valine isocyanide, we extracted WT *A. fumigatus* cultures grown under copper-limited conditions with deuterium-labeled solvents. If valine isocyanide was hydrated during extraction or subsequent solvent removal, some of the resulting *N-*formylvaline would incorporate one deuterium. In fact, HPLC-HRMS analysis of the deuterium-labeled extracts indicated that 5.7% of the observed *N*-formylvaline was deuterated (Fig. 2d), indicating that roughly 18% of the detected *N*-formylvaline in the MS analyses is derived from hydrolysis during or post extraction (also see Methods, Supplementary Table 2). The majority of the detected *N-*fomylvaline thus formed prior to extraction, either non-enzymatically or via one of the five putative isocyanide hydratases in *A. fumigatus* identified via a search for homologs of ThiJ/PfpI isocyanide hydratases from *Pseudomonas putida*^16^ (Supplementary Table 3). Likewise, 29% of fumicicolin A was observed to incorporate one deuterium when using deuterium-labeled extraction solvents, indicating that the majority of detected fumicicolin A is derived from hydrolysis of an ester of valine isocyanide and D-mannitol during extraction (fumicicolin C, Supplementary Fig. 3d).

### Conservation of fumivaline biosynthesis in *Penicillium commune*

Known producers of ergot alkaloids include the fungal genera *Claviceps*, *Penicillium*, *Aspergillus*, and *Epichloë*^12,13^. Many *Aspergillus* and *Penicillium* spp. produce ergoclavines as final products, whereas the *Claviceps* and *Epichloë* spp. instead produce d-lysergic acid derivatives, ergoamides, and ergopeptines (Supplementary Fig. 3a). *crmA* homologs are conserved in the ergot alkaloid producing *P. commune* as well as in the non-ergot alkaloid producing *P. expansum* (Fig. 1a, Supplementary Fig. 1, and Supplementary Data 1). Further, the ergot alkaloid BGC in *P. commune*^17–19^ (Fig. 2b and Supplementary Table 4), like the ergot alkaloid BGC in *A. fumigatus*, includes the complete festuclavine pathway (*dmaW, easA*, and *easC-G*, Fig. 2a, b).

To determine whether production of *N*-formylvaline and fumivalines A and B is conserved in other fungal genera harboring both *crm* and ergot alkaloid BGCs, we analyzed the metabolome extracts from *P. expansum* and *P. commune* grown under both copper-limited and copper-replete conditions. The two *Penicillium* spp. produced *N-*formylvaline under copper-limited conditions, whereas neither *N-*formylvaline nor any of the other *crmA*-dependent metabolites we had identified in *A. fumigatus* were present under copper-replete conditions (Fig. 2c). These results confirmed that in *Penicillium* spp., as in *A. fumigatus*, production of *crmA*-dependent metabolites is strongly regulated by copper levels. In addition, we detected fumivalines A and B under copper-limited conditions in *P. commune* but not in *P. expansum* (Fig. 2c and Supplementary Fig. 3b), consistent with the presence of the festuclavine pathway in *P. commune* but not *P. expansum* (Fig. 2a, b). Taken together, our results show that fumivaline production requires *crmA*, the festuclavine pathway, and copper starvation.

Next we asked whether the putative precursors of the fumivalines, dehydrofestuclavine and valine isocyanide, can be exchanged between different fungi. For this purpose we used mixed cultures of *A. fumigatus* Δ*crmA*, which produces ergot alkaloids but not valine isocyanide, with two different strains producing valine isocyanide but lacking ergot alkaloid biosynthesis: *A. fumigatus* Δ*dmaW*, which is defective in ergot alkaloid production, or OE::*crmA* of *P. expansum*, which lacks the ergot alkaloid pathway. Fumivaline A was not detected in the co-culture of *A. fumigatus* Δ*crmA* and *P. expansum*, whereas appreciable amounts of fumivaline A were detected in the co-culture of the two *A. fumigatus* mutants (Supplementary Fig. 3e, f). The latter result suggests that valine isocyanide or ergot alkaloid precursors can be exchanged between the two *A. fumigatus* strains, but not between *A. fumigatus* Δ*crmA* and *P. expansum*.

### Conservation of *crmA*-dependent mannitol derivatives

Homology searches showed that *crmA* homologs are also found in several species that lack ergot alkaloid BGCs, e.g., *P. expansum*, as well as in several phytopathogenic fungi including *A. brassicicola, L. maculans*, and *C. heterostrophus* (Fig. 1a, Supplementary Fig. 1, and Supplementary Data 1). *crmA* and *crmB*, with the addition of an α/β-hydrolase between the two genes, are conserved in *A. brassicicola, L. maculans, and C. heterostrophus*, whereas homologs of the two transporters, *crmC* and *crmD*, were not identified in any of these species (Supplementary Fig. 1).

**Fig. 3:**
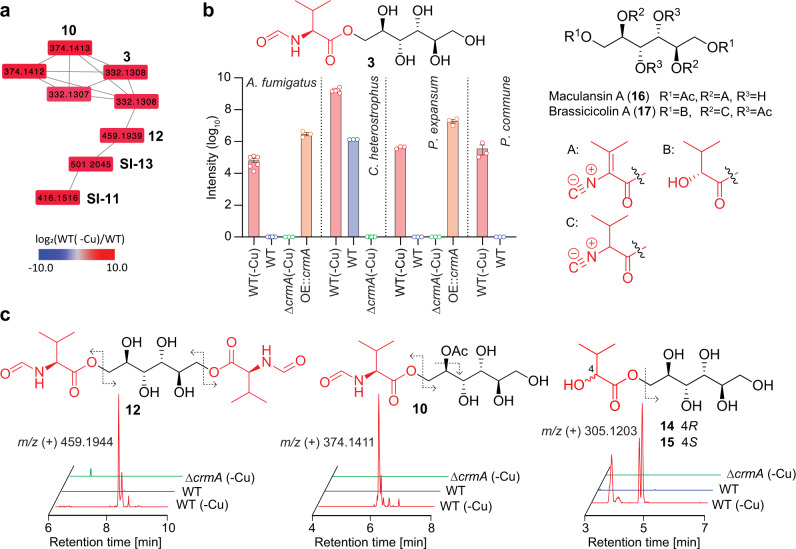
Identification of fumicicolin A and related metabolites. **a** Partial representation of MS2 network (cosine >0.7) for WT *C. heterostrophus* in ESI+, showing the cluster containing fumicicolin A (**3**) and the heterocicolins (**10**–**13**). Shown nodes are strongly upregulated in wildtype grown without copper (see Supplementary Fig. 4 for full network). **b** Relative abundance of fumicicolin A (**3**) in *A. fumigatus*, *C. heterostrophus*, and *Penicillium spp*., and its structural similarity with known d-mannitol derivatives, maculansin A (**16**) and brassicicolin A (**17**). **c** ESI + ion chromatograms of heterocicolins A (**10**), C (**12**), E (**14**), and F (**15**) in WT *C. heterostrophus* grown with (blue) or without copper (red) and Δ*crmA* (green) grown without copper. Dashed arrows indicate fragmentation in MS2 spectra. In **b**, bars represent mean ± s.e.m. with six independent biological replicates for *A. fumigatus* and *C. heterostrophus* wildtype under copper-limited conditions and three for the other strains/conditions. Source data are provided as a Source Data file.

To investigate the role of *crmA* in fungi that lack ergot alkaloid BGCs, we constructed *crmA*-deletion mutants for *P. expansum* and *C. heterostrophus*. Untargeted metabolomic comparison of *C. heterostrophus crmA* mutant and WT under copper-limited conditions followed by MS2 networking revealed several groups of *crmA*- and copper-dependent MS features (Fig. 3a and Supplementary Fig. 4). Detailed analysis of their MS2 spectra indicated that they represented a variety of d-mannitol derivatives, named heterocicolins A-F (**10**−**15**), that appeared to be related to fumicicolin A, which was also detected, along with *N*-formylvaline. In addition to *N*-formylvaline moieties, the heterocicolins additionally incorporate acetyl and/or 2-hydroxyisovaleryl moieties, similar to brassicicolin A (Fig. 3b, c and Supplementary Fig. 5a–c). The identities of heterocicolins C, E, and F were confirmed with synthetic standards (Supplementary Fig. 5d), whereas the structures of acetylated heterocicolins A, B, and D, which are produced as mixtures of isomers, were proposed solely based on MS2 spectra (Supplementary Fig. 5c). Similar to the *crmA*-dependent metabolites in *A. fumigatus*, production of heterocicolins A-F, fumicicolins A and B, and *N*-formylvaline was abolished or strongly downregulated under copper-replete condition (Fig. 3b, c and Supplementary Fig. 5a, c). Similarly, *crmA*-dependent production of *N-*formylvaline and fumicicolins A and B was observed in *P. expansum* under copper-limited but not copper-replete conditions (Fig. 3b and Supplementary Fig. 5a)*. crmA* overexpression in *P. expansum* resulted in increased production of *N-*formylvaline and fumicicolins A and B, as in *A. fumigatus*, and deletion of the gene eliminated production of all three metabolites (Fig. 3b and Supplementary Fig. 5a). Fumicicolins A and B were also detected in copper-limited *P. commune* (Fig. 3b and Supplementary Fig. 5a). The results indicate that *crmA* is required for the biosynthesis of the fumicicolins and related mannitol esters incorporating *N*-formylvaline.

The d-mannitol derived fumicicolins and heterocicolins are closely related to maculansin A (**16**), and brassicicolin A (**17**), previously described from *L. maculans*^20^ and *A. brassicicola*^21^, respectively (Fig. 3b), both of which also harbor *crmA* homologs (Supplementary Data 1), suggesting that **16** and **17** are derived from the *crmA* homologs in these species^20–22^. Differences in the composition of the *crm* cluster in these fungi may account for the observed differences in *crmA*-dependent d-mannitol derivatives. For example, the α/β-hydrolase present in the *crm* clusters of *C. heterostrophus, L. maculans*, and *A. brassicicol*a, but not *A. fumigatus*, could be responsible for the additional attachment of the hydroxyisovaleric acid or acetic acid moieties in the *crmA*-dependent d-mannitol derivatives in these species (Supplementary Fig. 1). α/β-hydrolase fold family of enzymes can have diverse biosynthetic roles^23^ and have been shown to serve as acyltransferases in natural products biosynthesis in *Acremonium chrysogenum*^24^ and *Caenorhabditis elegans*^25,26^.

### CrmA and associated products inhibit microbial growth from copper-starved *A. fumigatus*

**Fig. 4:**
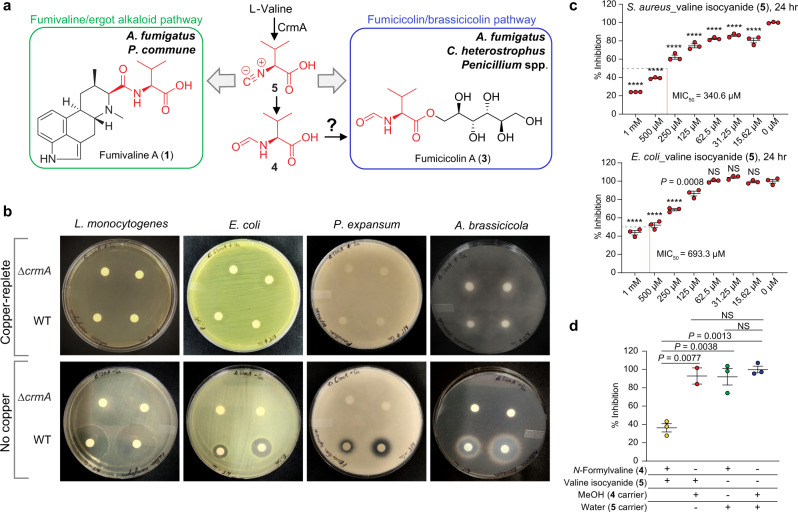
Putative biosynthesis of CrmA-derived metabolites in diverse fungi and antimicrobial activities. **a** Fumivaline biosynthesis was observed in *A. fumigatus* and *P. commune*, which has the ergot alkaloid BGC and a homolog for *crmA*, whereas fumicicolin biosynthesis was observed in *A. fumigatus*, *Penicillium* spp. and *C. heterostrophus*, all of which harbor *crmA* homologs. **b** Growth of *Listeria monocytogenes, Escherichia coli*, *Pencillium expansum*, and *Alternaria brassicicola* is inhibited when challenged with extracts from WT but not Δ*crmA A. fumigatus* grown without copper supplementation. Extracts from copper supplemented cultures do not inhibit microbial growth. **c** Valine isocyanide (**5**) significantly inhibits the growth of *Staphylococcus aureus* at all concentrations tested and inhibits the growth of *E. coli* at 125 μM and higher. MIC_50_, minimum inhibitory concentration to inhibit 50% growth. **d** Valine isocyanide (**5**) and *N*-formylvaline (**4**) show synergistic antifungal activity against *Candida auris* at 36 h. In **c** and **d**, bars represent mean ± s.e.m. with three independent biological replicates. One-way ANOVA with Dunnett’s multiple comparisons test was performed to assess if the differences in survival at the range of concentrations were statistically significant (at *p* < 0.05) from survival with solvent only (0 µM), ****p < 0.0001, NS, not significant. Source data are provided as a Source Data file.

Considering that several ergot alkaloids have antimicrobial properties^27^ and that several fungal isocyanides are antibacterial^28,29^, we tested whether CrmA-derived metabolites (Fig. 4a) have activity against a variety of bacterial and fungal species using disc assays. Disks soaked with crude extracts from WT *A. fumigatus*, the Δ*crmA* deletion mutant, and OE::*crmA* mutant, from cultures grown under copper-limited and copper-replete conditions, were placed on lawns of four bacteria (*Escherichia coli, Listeria monocytogenes, Pseudomonas aeruginosa*, and *Staphylococcus aureus*) and four fungi (*P. expansum, A. brassicicola, Candida albicans*, and *C. auris*). Zones of inhibition were observed against all fungi and all bacteria, except *P. aeruginosa*, from all OE::*crmA* extracts and, less consistently, from WT extracts grown under copper-limited conditions, in contrast to no inhibition zones observed from extracts of copper-replete WT or either extract of the Δ*crmA* mutant (Fig. 4b and Supplementary Table 5). These results suggested that CrmA metabolites contribute to the antimicrobial properties of *A. fumigatus* extracts.

Next we selected four CrmA-derived metabolites, valine isocyanide, *N*-formylvaline, fumivaline A, and fumicicolin A, for testing against *E. coli*, *S. aureus, C*. *auris* and *P. expansum*. Both bacteria were inhibited by valine isocyanide (Fig. 4c), but not the other metabolites (Supplementary Fig. 6a). No metabolite showed antifungal activity when applied alone, but a combination of valine isocyanide with *N*-formylvaline inhibited *C. auris* growth at early time points, suggestive of possible synergy or additive effects of the two compounds (Fig. 4d and Supplementary Fig. 6b) against this pathogen, which is listed by the CDC as an emerging global health threat due to its inherent antifungal resistance^30^. Valine isocyanide had no significant impact on growth *of A. fumigatus* (Supplementary Fig. 7a). Taken together, these data suggested that valine isocyanide, possibly in combination with other *A. fumigatus* metabolites, was partly responsible for the observed *crmA*-dependent activity of *A. fumigatus* extracts against bacteria and may contribute to antifungal activities as well; all in the context of environments devoid or poor in copper availability.

## Discussion

**Fig. 5:**
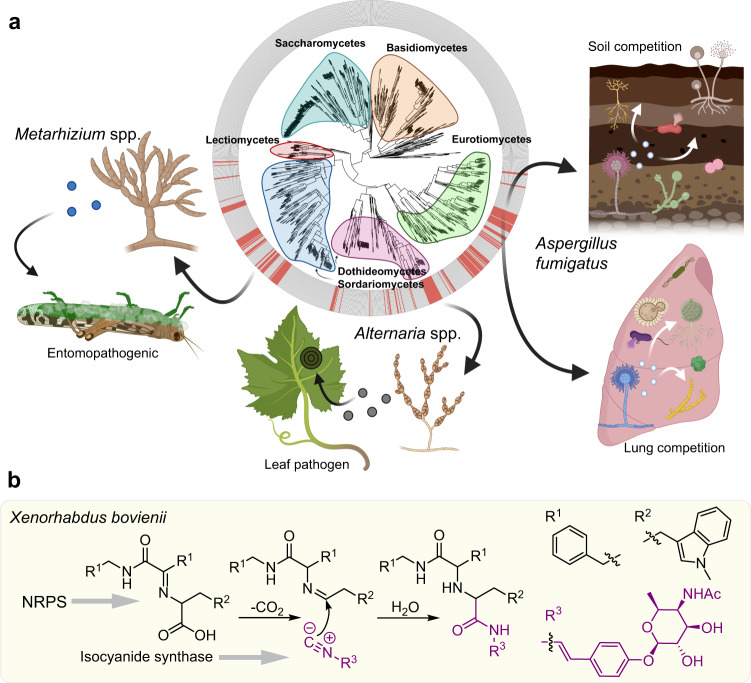
Phylogenetic tree and proposed functions of CrmA-derived metabolites in fungal ecology. **a** CrmA proteins are found in three fungal taxa (for details, see Supplementary Data 1). CrmA is primarily found in pathogenic fungi including the insect pathogens *Metarhizium, Cordyceps* and *Beauveria* and the fungal parasite *Trichoderma* (Sordariomycetes), leaf blight pathogens *Bipolaris*(e.g., *Cochliobolus*) and *Alternaria* (Dothideomycetes) as well as in opportunistic pathogens and saprophytic *Aspergillus* and *Pencillium* spp. and the dermatophytic genus *Trichophyton* (Eurotiales). Our model proposes that CrmA metabolites are synthesized under copper starvation, where they act as virulence factors facilitating copper acquisition during fungal/host encounters or as antimicrobial compounds during competition in copper-limited niches. **b** Proposed rhabdoplanin A biosynthesis via an Ugi-like reaction in bacteria^9^. Panel (**a**) was created with BioRender.com.

Filamentous fungi synthesize specific natural products to facilitate survival in response to various environmental stressors. Natural products can be elicited in response to abiotic stimuli, for example, UV induction of melanins protects fungal spores from harmful radiation^15,31,32^, or in response to competing microbes, where antibacterials^33^ or antifungals^34^ can confer competitive advantages to the producing fungus. Limitation of critical resources may also impact microbial secondary metabolite synthesis, as suggested by modulation of bacterial iron uptake genes dependent on confrontations with WT or a secondary metabolite mutant of *Penicillium*^35^. Like iron, copper levels are tightly regulated by microbes: copper is essential, but too much is toxic^36,37^. *A. fumigatus* maintains copper homeostasis in copper-deficient environments via the transcription factor MacA that activates copper importers during times of copper starvation^4^. Here we present a new paradigm in which MacA regulates the production of *crmA*-dependent antibiotics that inhibit the growth of potential *A. fumigatus* competitors under copper-deficient conditions. Our data support a view that *crmA* metabolites may function as conserved fitness factors in copper-deficient environments across fungal taxa (Fig. 5a and Supplementary Data 1). Valine isocyanide was efficient in inhibiting bacterial growth (Fig. 4c). This could be due to direct toxicity and/or the result of its copper chelating properties, as shown for other isocyanides including the *A. fumigatus* metabolite xanthocillin^28^. The antifungal effects of crude extracts of WT grown in copper-limited conditions may result from additive effects or synergy of valine isocyanide with other metabolites (Fig. 4b, d), including both CrmA- and non-CrmA-derived compounds. For instance, we found that in addition to CrmA metabolites, copper-starved *A. fumigatus* also produced higher quantities of helvolic acid, a known antifungal agent^38–40^ (Supplementary Fig. 7b). *A. fumigatus* occupies diverse primary niches, e.g., the lung as a common opportunistic pathogen, and as a common saprophyte in organic debris in fields and the rhizosphere where any advantage in the competition for scarce nutrients can be a significant fitness attribute (Fig. 5a). More detailed elucidation of the biological functions of the CrmA-derived metabolites we identified, e.g., to investigate synergistic activities, will require additional method development to ensure stability and accurate quantification of the involved metabolites, given that our isotope labeling studies demonstrated that valine isocyanide and its mannitol ester, fumicicolin C, are highly susceptible to hydrolysis and reaction with other nucleophiles.

Our analysis of CrmA-derived metabolites revealed biosynthetic pathways that produce (i) a new class of ergot alkaloids derived from Strecker-like peptide formation and (ii) valine isocyanide d-mannitol derivatives, whereby both pathways are strongly induced by copper starvation (Fig. 4a). In the former pathway, formation of the peptide bond in fumivaline biosynthesis appears to result from the nucleophilic attack of an isocyanide on an imine intermediate of ergot alkaloid biosynthesis, an unprecedented reaction in fungi that yields a new class of ergot alkaloids. Recently, a similar reaction was reported to be responsible for the formation of a peptide bond in the rhabdoplanins, a family of natural products isolated from the bacterium *Xenorhabdus bovienii*^9^ (Fig. 5b). Formation of the peptide bond in the rhabdoplanins was proposed to proceed as an Ugi-like reaction involving a decarboxylation step to generate the imine intermediate to which the isocyanide is then coupled, effectively resulting in replacement of the carbon lost by decarboxylation with the carbon from the isocyanide (Fig. 5b). In contrast, fumivaline A biosynthesis appears to proceed via nucleophilic attack of the isocyanide to the imine intermediate (Fig. 2a), thus producing a new ergot alkaloid carbon scaffold.

Ergot alkaloids have been primarily shown to be active against vertebrates ranging from insects to humans^41,42^ (Fig. 5a), and their pharmacological activities have been harnessed for diverse clinical uses^43^. Interestingly ergot alkaloid synthesis by insect pathogenic fungal *Metarhizium* spp. is tightly regulated and associated with insect colonization^41^, and we note that *A. fumigatus* ergot alkaloids are toxic to insects^44^. Regardless of fumivaline function, the intriguing reactivity of isocyanides shows their potential to function as versatile building blocks in the biosynthesis of other yet uncharacterized natural products^9^.

The valine isocyanide d-mannitol pathway, on the other hand, requires CrmA and the polyol, d-mannitol, where acylation, likely by an as-yet-unknown acyl transferase, with *N*-formylvaline or valine isocyanide followed by hydration yields fumicicolin A. Mannitol is a common sugar across the fungal Kingdom and thought to provide protection against osmotic stress^45,46^. Its role as a precursor for CmrA-dependent metabolites appears to be conserved in phytopathogenic fungi, such as *A. brassicicola, L. maculans, and C. heterostrophus*. In these species, CrmA homologs are located next to α/β-hydrolases that could be involved in the attachment of additional acyl moieties^26^. Notably, the CrmA homologs in these clades contain an additional transporter domain (Fig. 1a) that may also contribute to CrmA pathway metabolism in these fungi. The varying level of conservation of the other genes in the *A. fumigatus Crm* gene cluster, *crmB*, *C*, and *D*, in more distantly related species is consistent with a model in which CrmA-derived valine isocyanide is integrated into different pathways in different species, and is consistent with our finding that none of the other cluster genes are required for the *CrmA-*dependent metabolites in *A. fumigatus*.

Fumicicolin-related metabolites identified from phytopathogenic fungi, such as brassicicolin A synthesized by *A. brassicicola* and maculansins A and B produced by *L. maculans*, are host-selective phytotoxins^8,20^. Phytotoxin synthesis by leaf pathogens results in necrotic lesions in plant tissues and provides a source of easily obtained nutrients for the pathogen. A recent study of the plant pathogenic fungus *Sclerotinia sclerotiorum* showed that host copper was transported from healthy leaf tissues to necrotic tissues during infection, thus supplying the fungus with access to this micronutrient^47^. We speculate that the low copper availability in leaf tissues may be the signal to induce CrmA-derived phytotoxins which, in a similar manner as with *S. sclerotiorum*, could redirect copper supply to necrotic tissues colonized by the fungi (Fig. 5a).

The requirement of physically separated loci for fumivaline biosynthesis (conserved in both *A. fumigatus* and *P. commune*) demonstrates the complex signaling network fungi use to couple expression of non-clustered genes to increase unique chemical diversity. Although most secondary metabolites are reported to be generated from a single locus, examples of natural products requiring genes found in more than one locus are known, such as aspercryptin and nidulan A from *A. nidulans*^48,49^, aflatrem in *A. flavus*^50^, T toxin in *C. heterostrophus*^51^ and trichothecenes in some *Fusarium* spp.^52^. These examples suggest that greater consideration of cross-cluster exchange of intermediates could reveal other intriguing pathways. Our work further demonstrates that BGCs are still invisible to current bioinformatics tools (ICSs are not currently identified by BGC algorithms such as antiSMASH) but inferred from gene expression pattern changes in response to environmental stimuli, here copper starvation, can uncover unexpected chemical space. Our study shows that this approach can enable major advances in understanding fungal secondary metabolism, including genuinely new biosynthetic pathways and small-molecule functions. Given that homology searches revealed a large number of CrmA and other ICS homologs in fungi, it seems likely that isocyanides contribute to the production of many as-yet-undiscovered compounds.

In summary, our data show that the analysis of ICS-generated metabolites can reveal not only intriguing new structures and biosynthetic strategies but also striking connections of secondary metabolism with copper homeostasis. Our previous work on the *A. fumigatus* xanthocillin ICS-BGC showed that xanthocillin and xanthocillin-like isocyanides bind copper, deprive competing microbes of copper, and may facilitate copper uptake^28^. This latter study, coupled with the copper regulation of CrmA-dependent antimicrobials reported here, makes a strong case for micronutrient scarcity regulating fungal secondary metabolites biosynthesis to enable starvation survival via complementary ecological strategies (Fig. 5a).

## Methods

### Strains, media, and growth conditions

The fungal strains used in this study are listed in Supplementary Table 6. *Aspergillus* and *Penicillium* strains were maintained as glycerol stocks and activated on solid glucose minimal medium (GMM)^53^ at 37 °C. Growth media were supplemented with 0.56 g uracil L^−1^, 1.26 g uridine L^−1^, 1.0 g arginine L^−1^ for *pyrG* and *argB* auxotrophs, respectively. Unless otherwise noted, all *Aspergillus fumigatus* strains were grown at 37 °C and *Penicillum* strains were grown at 28 °C. *Cochliobolus heterostrophus strains* were activated on a complete medium with xylose^54^. For metabolomic analysis, strains were inoculated (1.0 × 10^6^ spores per mL) into 50 mL GMM^53^ in a 125-mL Erlenmeyer flask shaking at 200 rpm for 72 h. *Escherichia coli* strain DH5α was propagated in LB medium with appropriate antibiotics for plasmid DNA.

### Gene cloning, plasmid construction, and genetic manipulations

All fungal transformations were accomplished by polyethylene glycol (PEG) based methods as previously described^55,56^.

#### *A. fumigatus* overexpression *crmA*

For the construction of the *crmA* overexpression cassettes, two 1000–1500-bp fragments immediately upstream and downstream of the AFUA_3G13690 (CrmA) translational start site were amplified by PCR from AF293 genomic DNA. *A. parasiticus pyrG::A. nidulans gpdA(p)* as the selectable marker and overexpression promoter, respectively was amplified from the plasmid pJMP9^57^. The three fragments were fused by double joint PCR^58^ and then amplified using two nested primers to generate the deletion cassette (Supplementary Table 7). The overexpression cassette was transformed into strain TFYL45.1 to create strain TFYL82.2. Multiplex PCR was used to confirm the integration of the overexpression cassette at the native locus (data not shown). Single integration of the transformation construct was confirmed by Southern hybridization using two restriction enzyme digests (*Sca*I and *Mlu*I) and both the P-32 labeled 5′ and 3′ flanks (Supplementary Fig. 8a). To create a prototrophic strain (TJW209) of TFYL82.2, *A. fumigatus argB* was amplified using primers (fumargBF and fumargBR in Supplementary Table 7), transformed, and confirmed by PCR and sequencing (data not shown).

#### *A. fumigatus* deletion *crmBC*

TJW193.3 strain was made by a modified double joint method^58^. 1 kb DNA fragment of *crmD* open reading frame (ORF), a 2 kb DNA fragment of *A. parasiticus pyrG* and a 1 kb DNA fragment of *crmA* ORF. About 30 μL of Sephadex^®^ G-50 purified third-round PCR product was used for fungal transformation in TFYL80.1. All of the following fungal transformations were done using the polyethylene glycol (PEG) based method previously described^55^. *crmBC* deletants were confirmed by PCR and Southern blot (Supplementary Fig. 8b).

#### *P. expansum PEX2_062980* deletion and overexpression

For the deletion of the *P. expansum crmA* (PEX2_062980), ~1 kb upstream and downstream DNA regions of PEX2_062980 ORF were amplified by PCR with tails homologous to the hygromycin resistance self-excising β-rec/*six* blaster cassette^59^. Double joint PCR was used to build the knock-out construct^58^. For the overexpression of *P. expansum crmA*, ~1 kb upstream DNA region and ~1 kb of the ORF were amplified and fused to the overexpressed cassette, which consisted of the hygromycin resistance gene and the constitutive promoter *H2Ap*. The fungal transformation was done as described previously^55^. Twelve hours after transforming TJT14.1, an overlay of GMM (5 mL) containing sorbitol (1.2 M) and hygromycin (100 μg/mL) was put on top. The plates were incubated at 25 °C. The resulting transformants were subcultured twice on GMM containing hygromycin (100 μg/mL). The transformants were confirmed by PCR (Supplementary Fig. 8c). All primers for current gene cloning, plasmid construction, and genetic manipulations are listed in Supplementary Table 7.

#### *C. heterostrophus crmA* (JGI protein ID: 1220253, Genbank: EMD96666) deletion

Deletion of *crmA* was accomplished by PEG-mediated protoplast transformation using the split-marker approach^56^. The 5′ and 3′ regions flanking *crmA* were amplified from genomic DNA using primers, which included 5′ extensions complementary to those used to amplify the hygromycin B selectable marker (*HygB*). The *HygB* cassette was amplified from the plasmid pUCATPH^60^ as two separate overlapping fragments. The 5′ and 3′ *crmA* flanks were separately connected to the two *HygB* fragments by fusion PCR, generating two constructs. Targeted integration of the *HygB* cassette, and thus deletion of *crmA*, was accomplished by homologous recombination between the regions flanking *crmA*, and between the overlapping fragments of the *HygB* cassette. Candidate transformants were selected on 20 mL regeneration medium overlain with 10 mL 1% agar containing 150 μg/mL hygromycin B. Primers internal to *crmA* were used to screen candidates for the absence of *crmA*, while primers external to the original 5′ and 3′ flanks were used with primers internal to the *HygB* cassette to confirm targeted integration of the cassette. All primers are listed in Supplementary Table 7.

### Nucleic acid analysis

Plasmid preparation, digestion with a restriction enzyme, gel electrophoresis, blotting, hybridization, and probe preparation were performed by standard methods^61^. *Aspergillus* DNA for diagnostic PCR was isolated using the previously described method^62^. Sequence data were analyzed using the LASERGENE software package from DNASTAR.

### Analytical methods and equipment overview

#### NMR spectroscopy

NMR spectroscopy was performed on a Varian INOVA 600 MHz NMR spectrometer (600 MHz ^1^H reference frequency, 151 MHz for ^13^C) equipped with an HCN indirect-detection probe or a Bruker Avance III HD (800 MHz ^1^H reference frequency, 201 MHz for ^13^C) equipped with a 5-mm CPTCL ^1^H-^13^C/^15^N cryoprobe. Non-gradient phase-cycled dqfCOSY spectra were acquired using the following parameters: 0.6 s acquisition time; 400–600 complex increments; 8, 16, or 32 scans per increment. HSQC and HMBC spectra were acquired with these parameters: 0.25 s acquisition time, 200–500 increments, and 8–64 scans per increment. ^1^H, ^13^C-HMBC spectra were optimized for *J*_*H*,C_ = 6 Hz. HSQC spectra were acquired without decoupling, which allowed to measure one-bond proton-carbon coupling constants (^1^*J*_*H*,C_). NMR spectra were processed and baseline corrected using MestreLabs MNOVA software packages.

#### Mass spectrometry

LC−MS was performed on a Thermo Fisher Scientific Vanquish UHPLC system coupled with a Thermo Q-Exactive HF hybrid quadrupole-orbitrap high-resolution mass spectrometer equipped with a HESI ion source. Mass spectrometer parameters were used as spray voltage 3.5 kV, capillary temperature 380 °C, probe heater temperature 400 °C; 60 sheath flow rate, 20 auxiliary flow rate, and one spare gas; S-lens RF level 50, resolution 240,000, AGC target 3 × 10^6^. The instrument was calibrated weekly with positive and negative ion calibration solutions (Thermo Fisher). Each sample was analyzed in negative and positive ionization modes using an *m/z* range of 100 to 800.

#### Chromatography

Flash chromatography was performed using a Teledyne ISCO CombiFlash system. Semi-preparative chromatography was performed on an Agilent 1100 series HPLC system using Agilent Zorbax Eclipse XDB-C8 or XDB-C18 columns (25 cm × 10 mm, 5 μm particle diameter).

### Metabolite extraction and LC–MS analysis

Liquid fungal cultures (50 mL), including fungal tissue and medium, were frozen using liquid nitrogen and lyophilized. The lyophilized residues were extracted with 20 mL of methanol for 2.5 h with vigorous stirring. Extracts were pelleted at 5000×*g* for 5 min, and supernatants were transferred to 40 mL glass vials. Samples were then dried in a SpeedVac (Thermo Fisher Scientific) vacuum concentrator. Dried materials were resuspended in 1 mL of methanol and centrifuged to remove insoluble materials, and supernatants were transferred to 2 mL HPLC vials and subjected to UHPLC-HRMS analysis, as follows. An Agilent Zorbax RRHD Eclipse XDB-C18 column (2.1 × 100 mm, 1.8 μm particle diameter) was used with acetonitrile (organic phase) and 0.1% formic acid in water (aqueous phase) as solvents at a flow rate of 0.5 mL/min. A solvent gradient scheme was used, starting at 1% organic for 3 min, followed by a linear increase to 100% organic over 20 min, holding at 100% organic for 5 min, decreasing back, and holding at 1% organic for 3 min, for a total of 28 min.

### Feature detection and characterization

LC−MS RAW files were converted to mzXML format (centroid mode) using MSconvert (ProteoWizard), followed by analysis using the XCMS analysis feature in Metaboseek (metaboseek.com) based on the centWave XCMS algorithm to extract features^63^. Peak detection values were set as 4 ppm, 3 to 20 peak width, 3 snthresh, 3 and 100 prefilter, FALSE fitgauss, 1 integrate, TRUE firstBaselineCheck, 0 noise, wMean mzCenterFun, and −0.005 mzdiff. XCMS feature grouping values were set as: 0.2 minfrac, 2 bw, 0.002 mzwid, 500 max, 1 minsamp, and FALSE usegroup. Metaboseek peak filling values were set as 5 ppm_m, 5 rtw, and TRUE rtrange. The resulting tables of all detected features were then processed with the Metaboseek data explorer. To remove background-derived features, we first applied filters that only retained entries with a retention time window of 1 to 20 min and then applied maximum intensity (at least one repeat >10,000), and Peak Quality (>0.98) thresholds, as calculated by Metaboseek^10^. To select differential features, we applied a filter (using Metaboseek) to select entries with peak area ratios larger than 20 (upregulated by copper starvation in wildtype) or 5 (up in overexpression mutants compared to wild types). We manually curated the resulting list to remove false positive entries (i.e., features that upon manual inspection of raw data were not differential), which revealed a combined total of 236 differential features from positive and negative ionization modes. For verified differential features, we examined elution profiles, isotope patterns, and MS1 spectra to find molecular ions and remove adducts, fragments, and isotope peaks. The remaining features were put on the inclusion list for MS2 (ddMS2) characterization. Positive and negative ionization mode data were processed separately. To acquire MS2 spectra, we ran a top-10 data-dependent MS2 method on a Thermo Q-exactive-HF mass spectrometer with MS1 resolution 60,000, AGC target 3 × 10^6^, maximum injection time (IT) 100 ms, MS2 resolution 30,000, AGC target 5 × 10^5^, maximum IT 100 ms, isolation window 1.0 *m/z*, stepped normalized collision energy (NCE) 20 and 40 for both positive and negative ion modes, dynamic exclusion 3 s.

The initial comparative metabolomic analysis referred to in Fig. 1b was performed using six independent biological replicates. In addition, the *crmA*-dependence of the identified features was confirmed via HPLC-HRMS analysis of multiple additional sets of metabolite extracts used for compound isolation and bioassays.

### Chromatographic enrichment of compounds 1 and 6

Liquid fungal cultures (50 mL × 20), including fungal tissue and medium, were frozen using a dry ice acetone bath and lyophilized. The combined lyophilized residues were extracted with 500 mL of methanol for 3.5 h with vigorous stirring. Extracts were filtered over cotton, evaporated to dryness, and stored in 8 mL vials. Crude extracts were fractionated via semi-preparative HPLC using an Agilent XDB C-8 column (25 cm × 10 mm, 5 μm particle diameter) with acetonitrile (organic phase) and 0.1% acetic acid in water (aqueous phase) as solvents at a flow rate of 3.0 mL/min. A solvent gradient scheme was used, starting at 5% organic for 3 min, followed by a linear increase to 100% organic over 27 min, holding at 100% organic for 5 min, decreasing back to 5% organic for 0.1 min and holding at 5% organic for the final 4.9 min, for a total of 40 min. Further purification of fractions containing **1** and **6** was accomplished by semi-preparative HPLC using the same Agilent XDB C-8 column with acetonitrile (organic phase) and 0.1 M (pH 7.0) ammonium acetate in water (aqueous phase) as solvents at a flow rate of 3.0 mL/min with same gradient scheme as shown above.

### Analysis via Marfey’s method

Fumivaline A (0.5 mg) was hydrolyzed in 0.5 mL of 6 N hydrochloric acid at 115 °C. After 2 h, the reaction vial was cooled down in ice water for 3 min. Then the hydrochloric acid was removed in vacuo and the dry material was resuspended in 0.5 mL of water, and dried three times to remove residual hydrochloric acid completely. The residue was dissolved in 100 μL of 1 N sodium bicarbonate, followed by the addition of 50 μL of 10 mg/mL L-FDAA (1-fluoro-2,4-dinitrophenyl-5-l-alanine amide) in acetone. The reaction mixture was incubated in a water bath at 80 °C for 3 min. Then 50 μL of 2 N hydrochloric acid was added to quench the reaction by neutralization. Additionally, 300 μL of aqueous 50% acetonitrile was added to the solution to dissolve the reaction products. A 2 μL aliquot of the reaction mixture was analyzed by LC/MS using the gradient scheme described above. The same procedure was performed for a metabolome fraction containing *N*-formylvaline. Standards of l-valine and d-valine were derivatized with l-FDAA as described above. The FDAA derivatives of valine derived from fumivaline A and *N*-formylvaline showed the same retention time as the FDAA derivative of l-valine, which established that the valine moieties in *N*-formylvaline and fumivaline A possessed L-configuration (Supplementary Fig. 2g, h).

### Fungal culture extraction with deuterium-labeled solvents

About 5 mL of 7:3 mixture of methanol-*d*_1_ and deuterium oxide were added to liquid fungal cultures (5 mL), including fungal tissue and medium and vigorously mixed. The sample was frozen using a dry ice acetone bath and lyophilized. The lyophilized residues were extracted with 2.5 mL of 7:3:10 mixture of methanol-*d*_1_, deuterium oxide, and water for 24 h with vigorous stirring. Extracts were pelleted at 5000 × *g* for 10 min, and supernatants were transferred to another 4 mL glass vials. Samples were then dried in a SpeedVac (Thermo Fisher Scientific) vacuum concentrator. Dried materials were resuspended in 100 µL of methanol and vortexed for 1 min. Samples were pelleted at 5000×*g* for 5 min, and supernatants were transferred to 2 mL HPLC vials. Clarified extracts were transferred to fresh HPLC vials and stored at −20 °C until analysis. Percent of valine isocyanide hydrolysis was calculated using the following formula: Ratio (%) = (deuterated *N*-formylvaline, %) × (abundance of H + abundance of D)/(abundance of D), which takes into account the ratio between hydrogen and deuterium in the extraction solvent, also considers the contribution from water in the culture media (Supplementary Table 2).

### MS2-based molecular networking

An MS2 molecular network^64^ was created using Metaboseek version 0.9.7 and visualized in Cytoscape^65^. Features were matched with their respective MS2 scan within an *m/z* window of 5 ppm and a retention time window of 15 s, using the MS2scans function. To construct a molecular network, the tolerance of the fragment peaks was set to an *m/z* of 0.002 or 5 ppm, the minimum number of peaks was set to 3, and the noise level was set to 2%. Once the network was constructed, a cosine value of 0.7 was used, the number of possible connections was restricted to 6 for positive ion mode, and the maximum cluster size was restricted to 200 for positive ion mode.

### Synteny analysis of *crmA* loci

To locate *crmA* homologs, a local BLAST^66^ search was conducted against a database of *A. fumigatus* (NCBI accession: GCF_000002655.1), *A. brassicicola* (ATCC96836)^67^, *C. heterostrophus* (GCA_000338975.1), and *P. commune* (GCA_000338975.1, sequenced generously received from Dr. Benjamin Wolfe)^19^. Protein domains were predicted for the eight genes flanking the left and right of all three *crmA* homologs by searching against the CDD v3.19 database on the NCBI conserved domains web tool^68^. The expected value threshold was kept at 0.01 and composition-based statistic adjustment was turned on. Clinker and clustermap.js was utilized to make comparisons of gene cluster similarity^69^. Similarity scores were calculated by Clinker and are based on the cumulative sequence identity of homologous sequences and the number of shared contiguous sequence pairs. Visualization of the results was done with clustermap.js.

### Identification of all crmA homologs

To locate all of the *crmA* homologs, we extracted the predicted *crmA* isocyanide synthase domain amino acid sequence from *A. fumigatus*. This was done to capture variations of the *crmA* gene that have homologous ICS regions. The following was run using BLAST+^70^ version 2.12.0 on 11/01/2021: blastp -query ICS_crmA.fa -remote -db nr -evalue 1e-6 -out ICScrmAHits.txt -outfmt “6 qseqid sseqid sacc sgi staxids sscinames scomnames sblastnames sskingdoms stitle length mismatch gapopen qstart qend sstart send sstrand evalue bitscore”. As only the *crmA* isocyanide region was used in this search, we filtered out hits that had a protein length of less than 1000 amino acids (for reference the crmA protein in *A. fumigatus* is 1537 amino acids). This prevented returned hits from proteins that were solely an ICS domain (Supplementary Data 1). To generate a visualization of these hits, species with homologs to the crmA protein were mapped onto a taxonomic tree that was generated by identifying single copy orthologs from BUSCO^71^, followed by an estimation of the maximum-likelihood phylogeny with IQ-TREE^72^.

### Antimicrobial assay using crude extracts

Antimicrobial assays were assessed for *Escherichia coli*, *Listeria monocytogenes*, *Staphylococcus aureus*, *Pseudomonas aeruginosa*, *P. expansum*, *A. brassicicola*, *Candida albicans*, and *C. auris* using Kirby–Baur’s disc-diffusion method^73^. A loopful of *E.coli* was inoculated in 4 mL of LB media and *L. monocytogenes* in 4 mL of BHI (brain–heart infusion) liquid media, followed by incubation at 37 °C in a shaker incubator at 200 rpm overnight. *C. auris* and *C. albicans* were grown on YEPD (yeast extract peptone dextrose) media at 33 °C for 48 h, a small amount of the yeast was scraped off of the plates using a sterile toothpick and resuspended in 4 ml 0.85% sodium chloride. A sterile cotton swab was used to dip and swab the yeast and overnight bacterial cultures, completely and evenly on the respective agar plates. 1 × 10^6^ spores from 5-day old *P. expansum* plates and 7-day old *A. brassicicola* plates were inoculated in 10 mL GMM top agar (GMM with 0.75% agar) and MEA top agar (MEA with 0.75% agar), respectively, before pouring them on the respective agar plates. The lyophilized crude extract samples were resuspended with 500 uL of 100% methanol and mixed well. Sterile 6 mm blank paper disks were individually injected with 25 µL of crude extracts from wildtype *A. fumigatus*, crmA deletion and crmA overexpression strains grown on GMM liquid media with copper and without copper. They were then placed at equal distances from each other on all the bacterial and fungal plates, immediately after their inoculation. The bacterial plates were incubated for 24 h at 37 °C, while the *P. expansum* plate was incubated at 25 °C for 3 days and *A. brassicicola* at 25 °C for 5 days. The Candida plates were incubated at 33 °C for 2 days. The presence or absence of antimicrobial susceptibility with all the extracts was determined and zones of inhibition were measured at the end of the respective incubation periods.

### Antimicrobial assay using purified metabolites

All antimicrobial assays with purified compounds were performed in flat-bottomed 96-well microtiter plates. Bacteria used in these assays (*E. coli* and *S. aureus)* were used from fresh plates of LB incubated at 30 °C overnight. Liquid cultures were raised in 3 mL of liquid LB medium by inoculating a single colony incubated at 30 °C with constant shaking at 250 rpm. Overnight cultures were harvested with centrifugation, washed 2X with phosphate-buffered saline, and resuspended in phosphate-buffered saline to be used as inoculum for the assays.

Samples of purified fumivaline A, synthetic fumicicolin A, synthetic (*S*)−2-isocyanoisovaleric acid, and *N-*formylvaline obtained from TCI America were resuspended to a final concentration of 10 mM in water, DMSO, and methanol respectively, which were treated as stocks. A concentration gradient was set up using these stocks for each metabolite against each bacterium. All metabolites were set up at half gradients; valine isocyanide with 1 mM as the highest and 15 µM as the lowest concentrations; fumivaline A and *N*-formylvaline with 250 µM and 4 µM as the highest and lowest concentrations respectively. Each well of the 96-well plate was inoculated to hold a final volume of 200 µL with 2.5% by volume of the desired metabolite (for fumivaline A and *N*-formylvaline) and 10% by volume of valine isocyanide. Corresponding solvents were used as positive control and gentamicin at 50 µg/mL was used as the negative control. Bacteria were added to obtain a final OD_600_ = 0.01. The plates were incubated for 24 h before OD_600_ was read again. Percent survival reported was calculated by normalizing OD_600_ at 24 h to OD_600_ at inoculation (0 h).

Antifungal assays were carried out against *C. auris* with the stocks prepared as above. Synergistic activities of valine isocyanide with *N*-formylvaline (see results in Fig. 4d) were tested in 96-well plates. The compounds were added at concentrations of 1 mM and 250 µM, respectively, with corresponding solvent combinations as a control in a total volume of 200 µL of YPD medium (10 g yeast extract, 20 g peptone, and 20 g glucose in 1 L). Three replicates were monitored per treatment. *C. auris* cells were grown overnight in a 1 mL YPD medium at 30 °C without shaking. The cells were washed in phosphate-buffered saline and added to YPD at a final concentration of 5 × 10^4^ cells/mL. The plates were incubated at 30 °C and OD_600_ readings were taken after 24–36 h. Percent survival reported was calculated by normalizing OD_600_ at 36 h to OD_600_ at inoculation (0 h).

Antifungal activity of valine isocyanide against *A. fumigatus* was tested using stocks prepared as above. The compound was tested with 5 mM as the highest concentration and a serial half dilution from 1 mM to 15.62 µM as the lowest concentration. About 100 µL of the *A. fumigatus* spores in GMM liquid media^74,75^ were inoculated to each well of the 96-well plate for a final concentration of 1 × 10^5^ cells/mL with 100 µL of each concentration of valine isocyanide in water. Water was used as negative control and polymyxin B^76^ at 40 mM was used as a positive control. Three replicates were monitored per treatment. The entire experiment was set up on ice and optical density was checked at 600 nm upon completion. The plate was then incubated at 37 °C for 24 h, after which contents of the wells were mixed using a multichannel pipette to resuspend fungi, and OD_600_ was measured again.

One-way ANOVA with Dunnett’s multiple comparisons test was performed to assess if the differences in survival at the range of concentrations were statistically significant (at *p* value <0.05) from survival with solvent only.

### Data and statistical analysis

LC–MS data were collected using Thermo Scientific Xcalibur software version 4.1.31.9. LC–MS data were analyzed using Metaboseek software version 0.9.7 and quantification was performed via integration in Excalibur Quan Browser version 4.1.31.9. NMR spectra were processed and baseline corrected using MestreLabs MNOVA software packages version 11.0.0-17609. All statistical analysis were performed with GraphPad Prism version 9.2 or Metaboseek version 0.9.7. The *P* values of datasets were determined by unpaired two-tailed Student’s *t*-test with a 95% confidence interval unless specified otherwise.

### Reporting summary

Further information on research design is available in the Nature Research Reporting Summary linked to this article.

## Supplementary information

Supplementary Information

Reporting Summary

Description of Additional Supplementary Files

Supplementary Dataset 1

Supplementary Dataset 2

## Data Availability

MS and MS2 data for all fungal metabolome samples analyzed in this study (*A. fumigatus*, *P. commune*, *P. expansum*, *C. heterostrophus*, co-culture of *A. fumigatus* and *P. expansum*, and deuterium labeling experiments) are available at the GNPS Web site (massive.ucsd.edu) under MassIVE ID number MSV000089206. The *crmA* sequence data used in this study are available in the NCBI database under accession code XP_754255 for *A. fumigatus*, XP_016596276 for *P. expansum*, and EMD96666 for *C. heterostrophus*. The sequence data used in this study are available in the NCBI database under accession code GCF_000002655.1 for *A. fumigatus*, GCA_008931945.1 for *P. commune*, GCA_000338975.1 for *C. heterostrophus*. The sequence data for *A. brassicicola* is available in the JGI Mycocosm database under accession code ATCC96836. Source data are provided with this paper.

## Source data

Source Data file
