# Supplementary material for: Copper starvation induces antimicrobial isocyanide integrated into two distinct biosynthetic pathways in fungi
Source: Nat Commun. 2022 Aug 16;13:4828. doi: 10.1038/s41467-022-32394-x (PMC9381783; doi:10.1038/s41467-022-32394-x)
Supplement: Supplementary file 1 — Supplementary Information [file 41467_2022_32394_MOESM1_ESM.pdf]

Copper starvation induces antimicrobial isocyanide integrated into two distinct biosynthetic pathways in fungi

Supplementary Information

Tae Hyung Won[1], Jin Woo Bok[2], Nischala Nadig[3], Nandhitha Venkatesh[4], Grant Nickles[5], Claudio Greco[2,§], Fang Yun Lim[2,§], Jennifer B. González[6,§], B. Gillian Turgeon[6], Nancy P. Keller[2,3]*, Frank C. Schroeder[1]*

[1]Boyce Thompson Institute and Department of Chemistry and Chemical Biology, Cornell University, Ithaca, NY, United States.

[2]Department of Medical Microbiology and Immunology, University of Wisconsin-Madison, Madison, WI, United States.

[3]Department of Bacteriology, University of Wisconsin-Madison, Madison, WI, United States.

[4]Department of Plant Pathology, University of Wisconsin-Madison, Madison, WI, United States.

[5]Department of Cellular and Molecular Biology, University of Wisconsin-Madison, Madison, WI, United States.

[6]Department of Plant Pathology and Plant-Microbe Biology, Cornell University, Ithaca, NY, United States.

*Correspondence to npkeller@wisc.edu and schroeder@cornell.edu

[§]Current address:

Claudio Greco: Department of Molecular Microbiology, John Innes Centre, Norwich NR4 7UH, United Kingdom.

Fang Yun Lim: Department of Chemistry, University of Washington, Seattle, WA, United States.

Jennifer B. González: 104 Peckham Hall, Nazareth College, 4245 East Avenue, Rochester, NY, United States.

Table of Contents

1. Supplementary Figures 1-8

2. Supplementary Tables 1-7

3. Supplementary Methods

4. NMR Spectroscopic Data and Spectra. Includes data for fumivaline A (**1**), fumicicolin A (**3**), *N*-formylvaline (**4**), (*S*)-2-isocyanoisovaleric acid (**5**), heterocicolins C (**12**), E (**14**), and F (**15**)

5. Supplementary References

1. Supplementary Figures

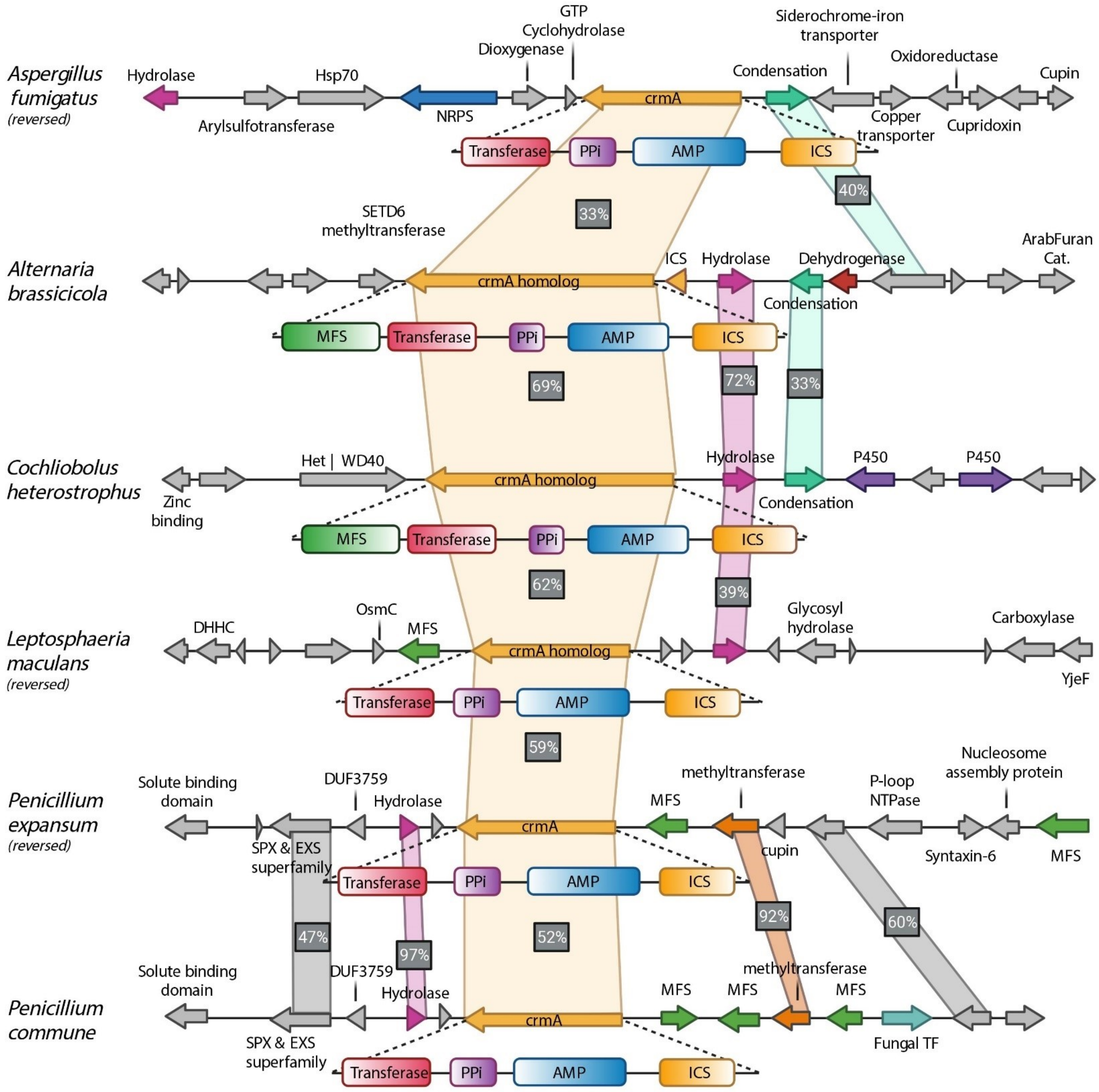

Supplementary Figure 1. Comparison of *crm* BGCs in various other fungi. Homologous genes are marked with colors based on their corresponding functions.

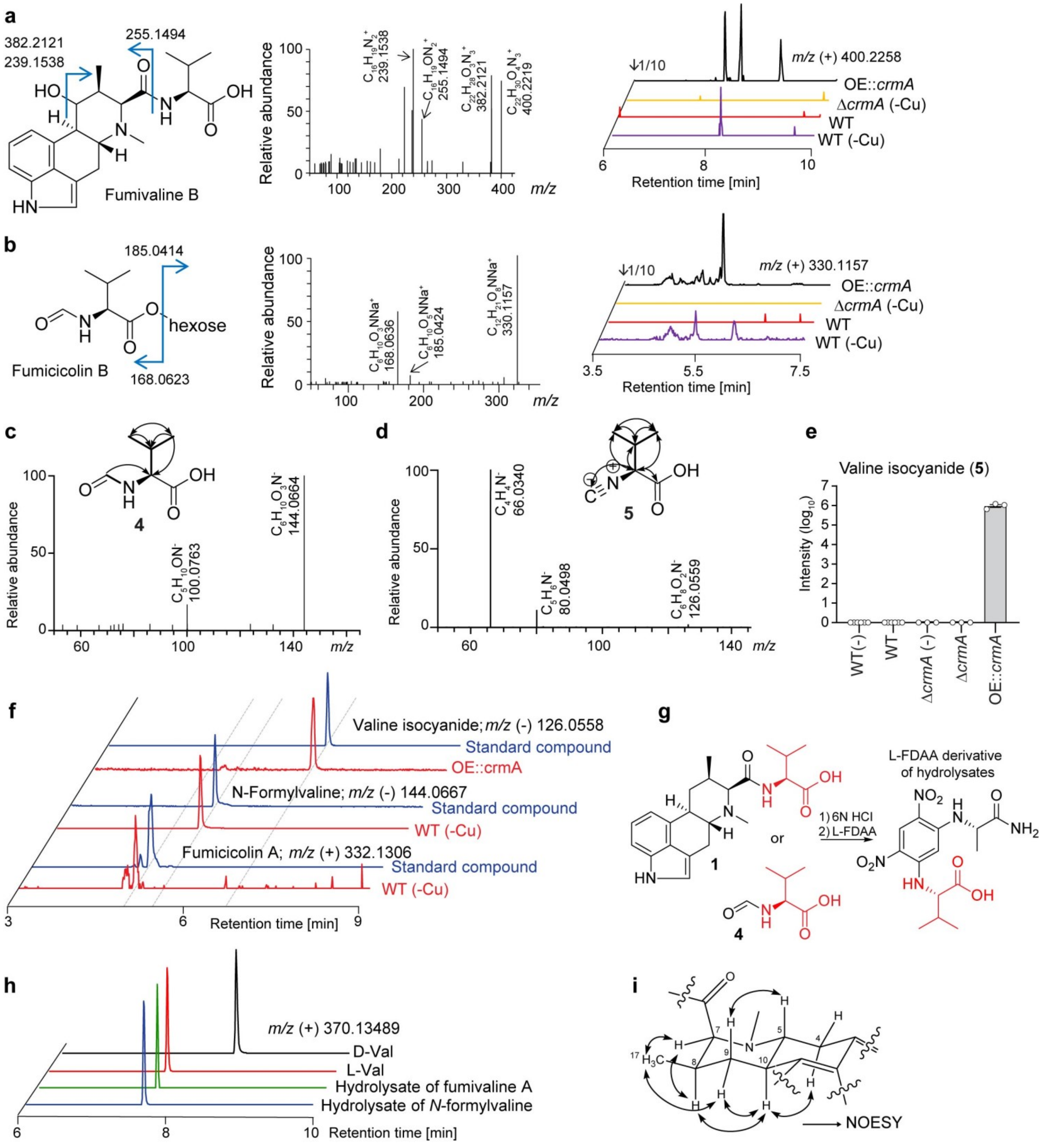

Supplementary Figure 2. Analysis of *crm*-dependent metabolites. **a**,**b**, Proposed structures, MS2 spectra, and ESI+ ion chromatograms of fumivaline B and *N*-formylvaline glycoside in *A. fumigatus*. **c**,**d**, COSY and HMBC correlations and MS2 spectra in ESI- mode of *N*-formylvaline (**4**) and (*S*)-2-isocyanoisovaleric acid (valine isocyanide, **5**). **e**, Relative abundance of valine isocyanide (**5**) in *A. fumigatus* (from extraction with non-deuterated solvents). **f**, Extracted ion chromatograms (EICs) of fumicicolin A (**3**), *N*-formylvaline (**4**), and valine isocyanide (**5**) in *A. fumigatus* and comparison with synthetic standards. **g**,**h**, Determination of the absolute configuration of valine moieties in *N*-formylvaline (**4**) and fumivaline A (**1**) using Marfey's method. Reaction

schemes for acid hydrolysis and FDAA derivatization (**g**), and EICs for L-FDAA derivatives of L-Val (red), D-Val (black), hydrolysate of *N*-formylvaline (blue), and fumivaline A (green) (**h**). **i,** Determination of the relative configuration of fumivaline A (**1**) based on NOESY correlations. In **e,** bars represent mean ± s.e.m. with six independent biological replicates for *A. fumigatus* wild type and thee for the other strains. Source data are provided as a Source Data file.

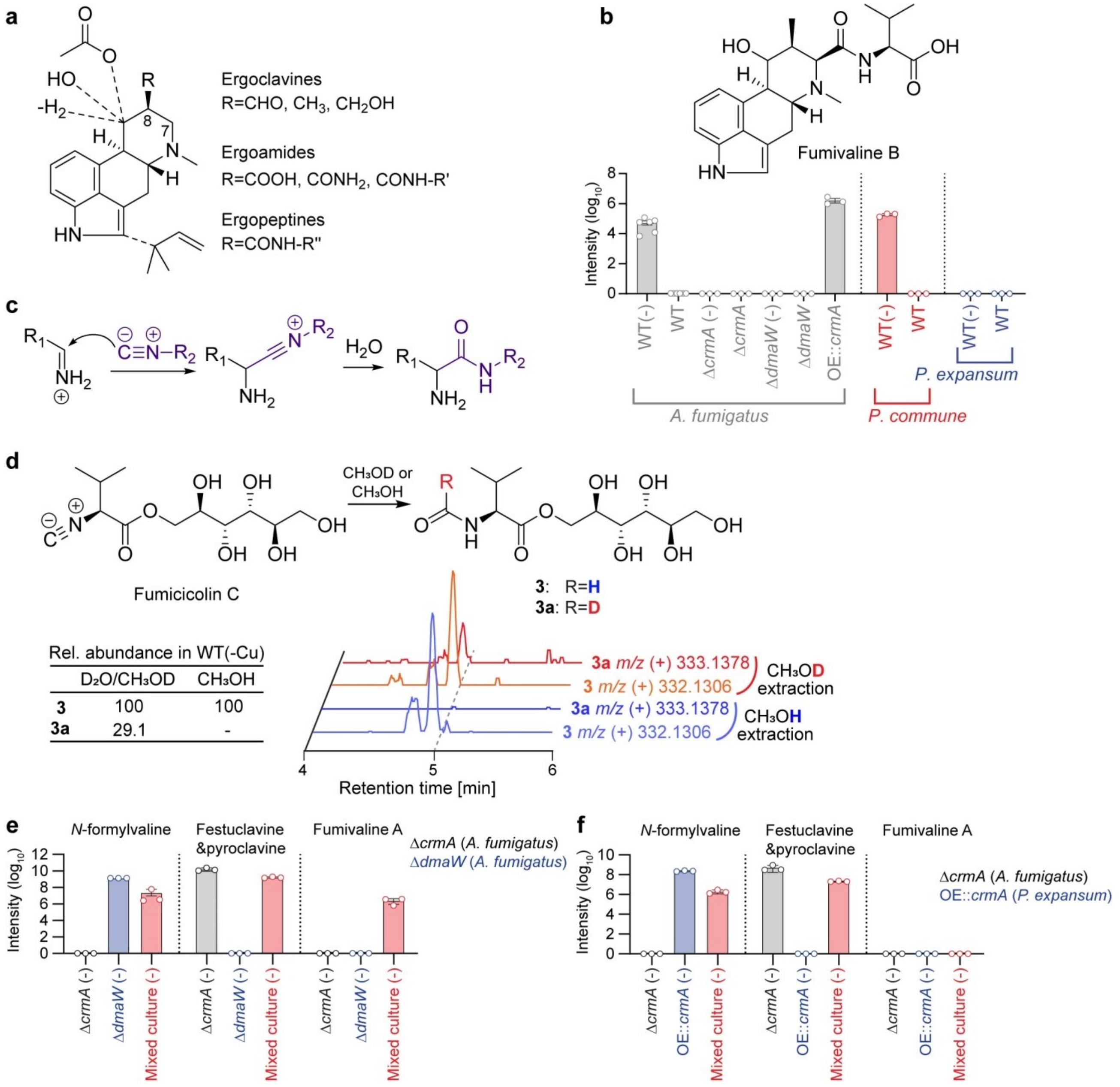

Supplementary Figure 3. Interaction of ergot alkaloid and *crmA* pathways. **a**, Comparison of the structures of the different ergot alkaloid families, ergoclavines, ergoamides, and ergopeptines. **b**, Relative abundance of fumivaline B in *A. fumigatus*, *P. commune*, and *P. expansum* (grey, red, and blue, respectively). **c**, Strecker amino acid synthesis. **d**, Relative abundances of fumicicolin A (**3**) and [7-^{2}H]-fumicicolin A (**3a**) derived from fumicicolin C, an ester of valine isocyanide and D-mannitol, in extracts of wild type *A. fumigatus* (grown without copper) extracted with deuterated or non-deuterated solvents. **e**,**f**, Relative abundances of *N*-formylvaline (**4**), festuclavine (**2**), pyroclavine, and fumivaline A (**1**) in pure and mixed cultures of Δ*crmA* and Δ*dmaW* of *A. fumigatus* (**e**) or Δ*crmA* of *A. fumigatus* and OE::*crmA* of *P. expansum* (**f**) under copper starvation conditions. In **b**, **e,** and **f**, bars represent mean ± s.e.m. with six independent biological replicates for *A. fumigatus* wild type and three for the other strains. Source data are provided as a Source Data file.

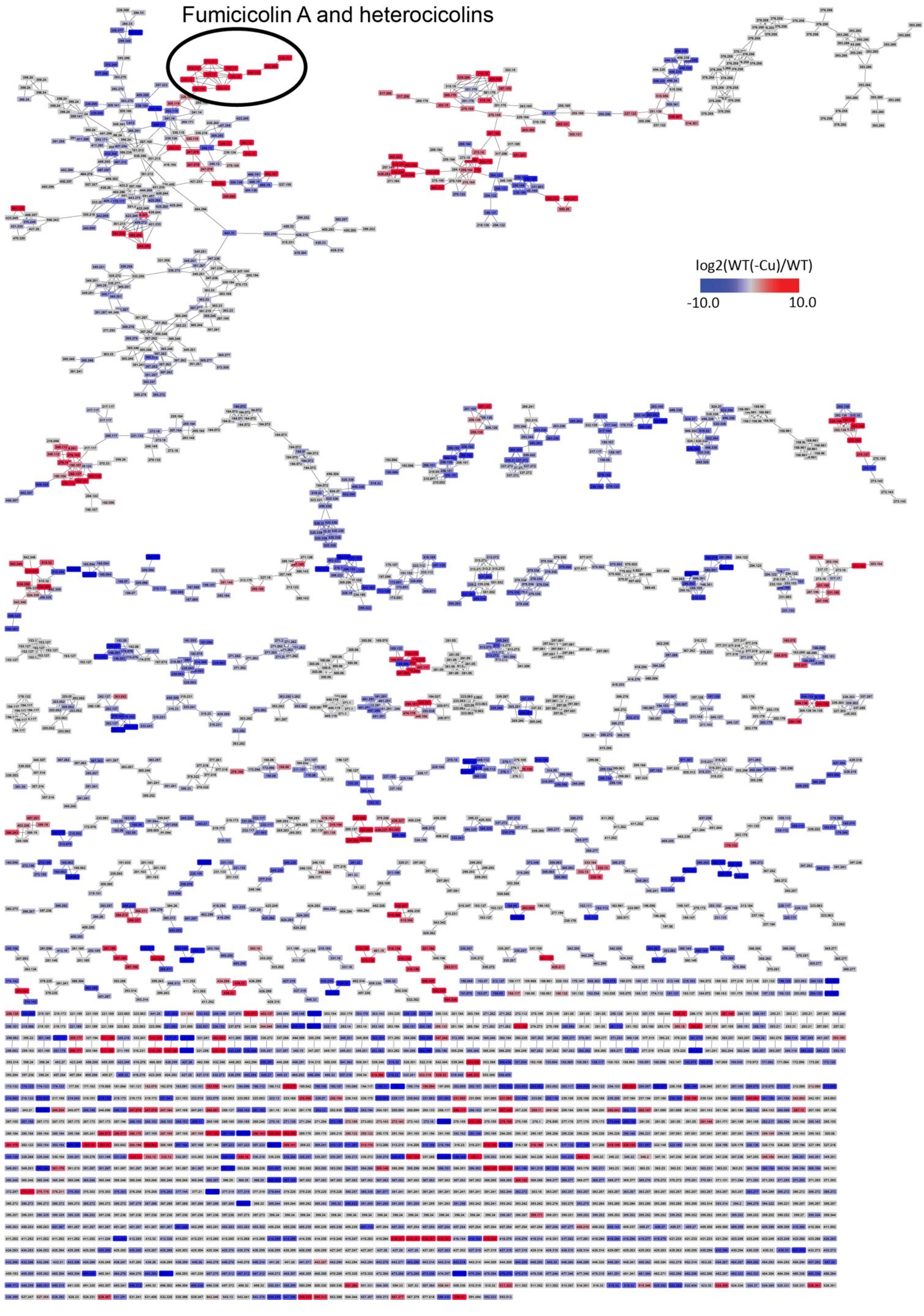
Fumicicolin A and heterocicolins
log2(WT(-Cu)/WT)
-10.0
10.0

Supplementary Figure 4. MS2 network of copper-dependent differential features in WT of *C. heterostrophus* in ESI+*.* Blue and red represent downregulated and upregulated features, respectively, in WT grown without copper relative to WT *C. heterostrophus* grown with copper. Black circles highlight the subnetworks for the most abundant differential MS features related to fumicicolin A (**3**) and the heterocicolins (**10-15**).

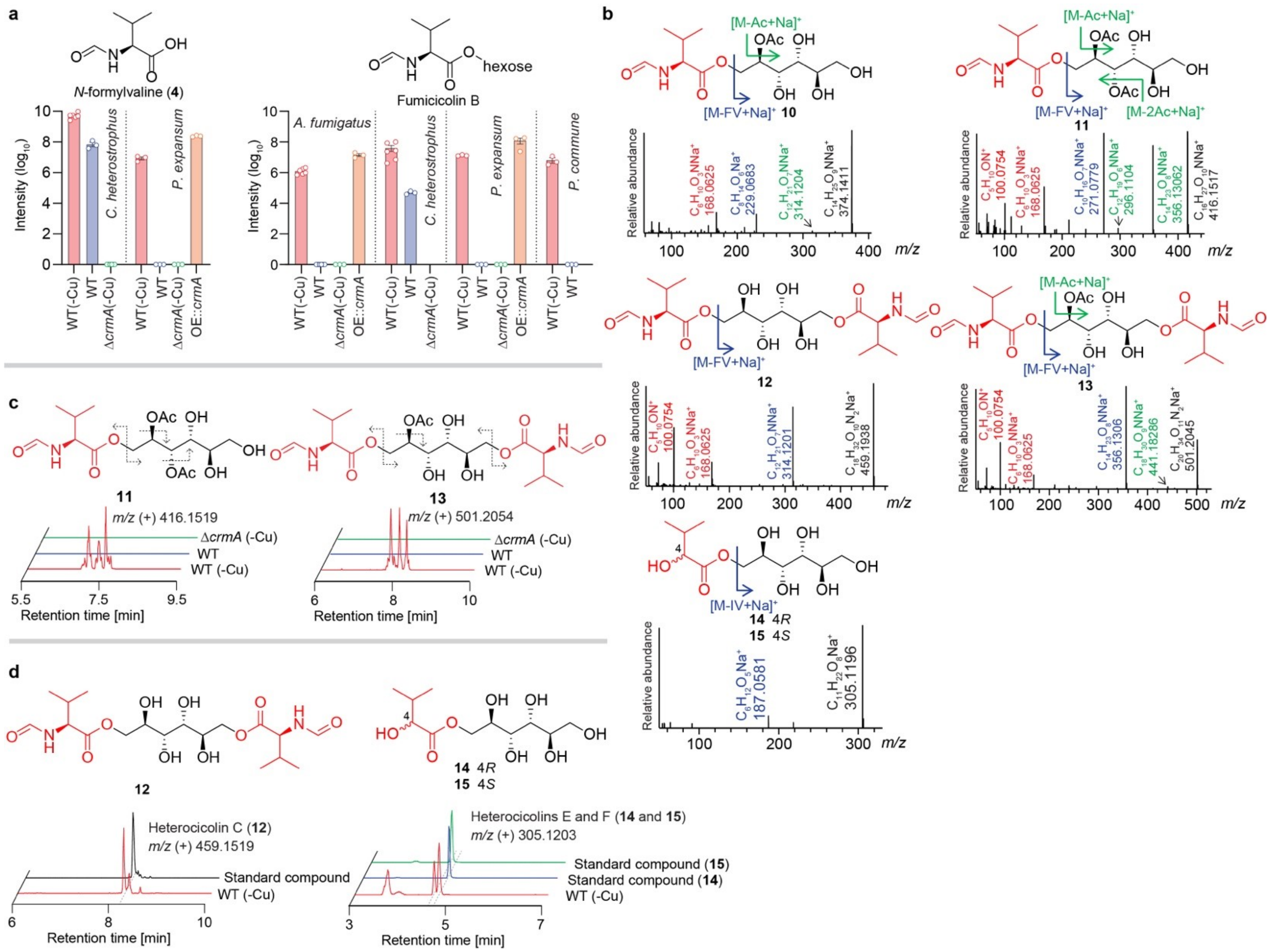

Supplementary Figure 5. Analysis of fumicicolin-like metabolites in different fungi. **a**, Relative abundances of *N*-formylvaline (**4**) in *C. heterostrophus* and *P. expansum*, and fumicicolin B in *A. fumigatus*, *C. heterostrophus,* and *Penicillium spp.* **b**, MS2 spectra of heterocicolins A-F (**10-15**). Red represents fragments derived from the *N*-formylvaline groups (FV, *N*-formylvaline and IV, 2-hydroxyisovaleric acid), blue represents fragments that include a D-mannitol moiety, and green represents fragments derived from loss of acetic acid (Ac). **c**, EICs of heterocicolins B (**11**) and D (**13**) in *C. heterostrophus*. WT was grown under both copper-limited (red) and copper-replete (blue) conditions, and the Δ*crmA* mutant was grown under copper-limited condition (green). Dashed arrows indicate fragmentation in MS2 spectra. **d**, EICs of heterocicolins C (**12**), E (**14**), and F (**15**) in *C. heterostrophus* and comparison with synthetic standards. In **a**, bars represent mean ± s.e.m. with six independent biological replicates for *A. fumigatus* and *C. heterostrophus* wild type under copper-limited conditions and three for the other strains, respectively. Source data are provided as a Source Data file.

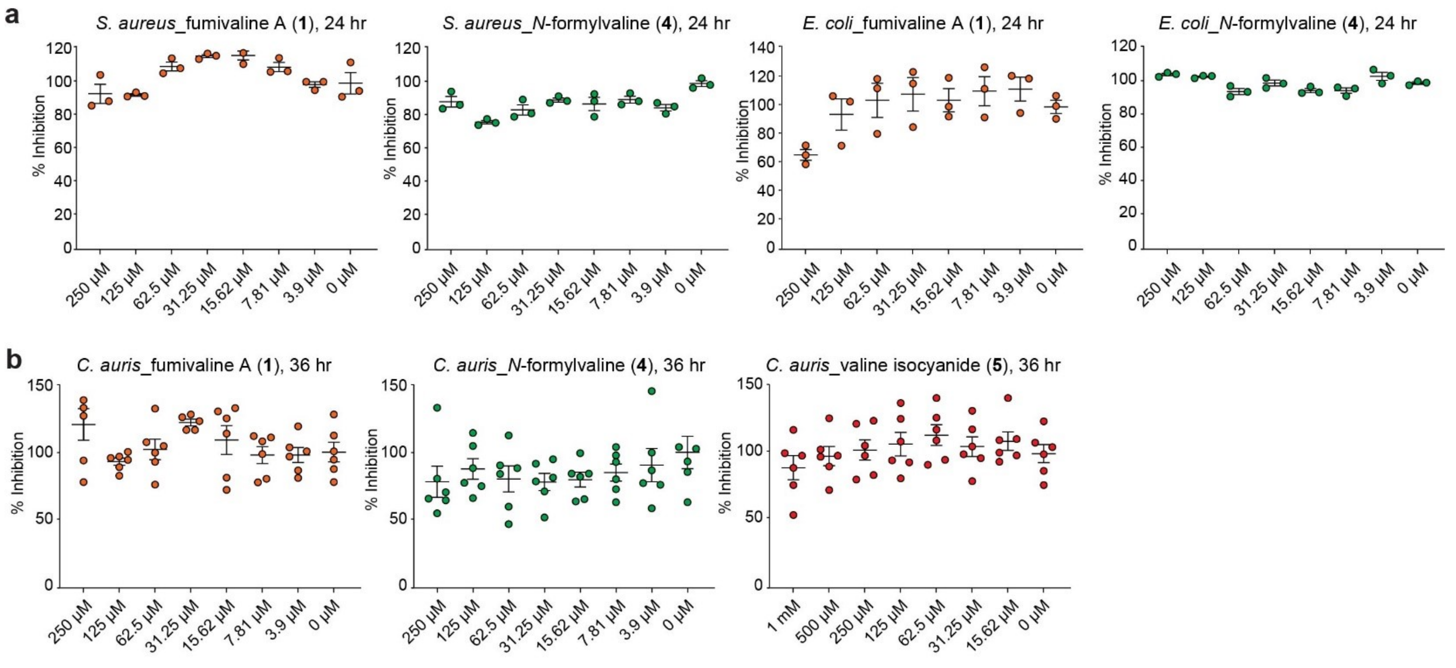

Supplementary Figure 6. a, Antimicrobial assays of fumivaline A (**1**) and *N*-formylvaline (**4**) against *E. coli* and *S. aureus*. **b**, Antimicrobial assays of fumivaline A (**1**), *N*-formylvaline (**4**), and valine isocyanide (**5**) against *C. auris*. In **a** and **b**, bars represent mean ± s.e.m. with three independent biological replicates for *S. aureus* and *E. coli,* and six for *C. auris*. Source data are provided as a Source Data file.

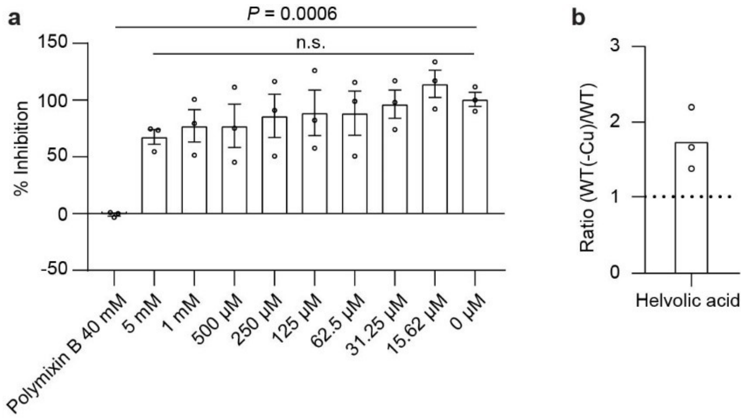

Supplementary Figure 7. Impact of valine isocyanide on *A. fumigatus*, and copper-dependency of helvolic acid production. **a**, Antimicrobial assays of valine isocyanide (**5**) against *A. fumigatus*. Valine isocyanide (**5**) showed no significant growth inhibition for *A. fumigatus* at all concentrations tested. Bars represent mean ± s.e.m. with three independent biological replicates. One-way ANOVA with Dunnett's multiple comparisons test was performed to assess if the differences in survival at the range of concentrations were statistically significant (at p-value<0.05) from survival with solvent only (0 µM). **b**, Copper-dependent production of helvolic acid. Abundance of helvolic acid in WT *A. fumigatus* grown under copper limited conditions relative to copper replete conditions. Bar represents mean of three independent biological replicates. Source data are provided as a Source Data file.

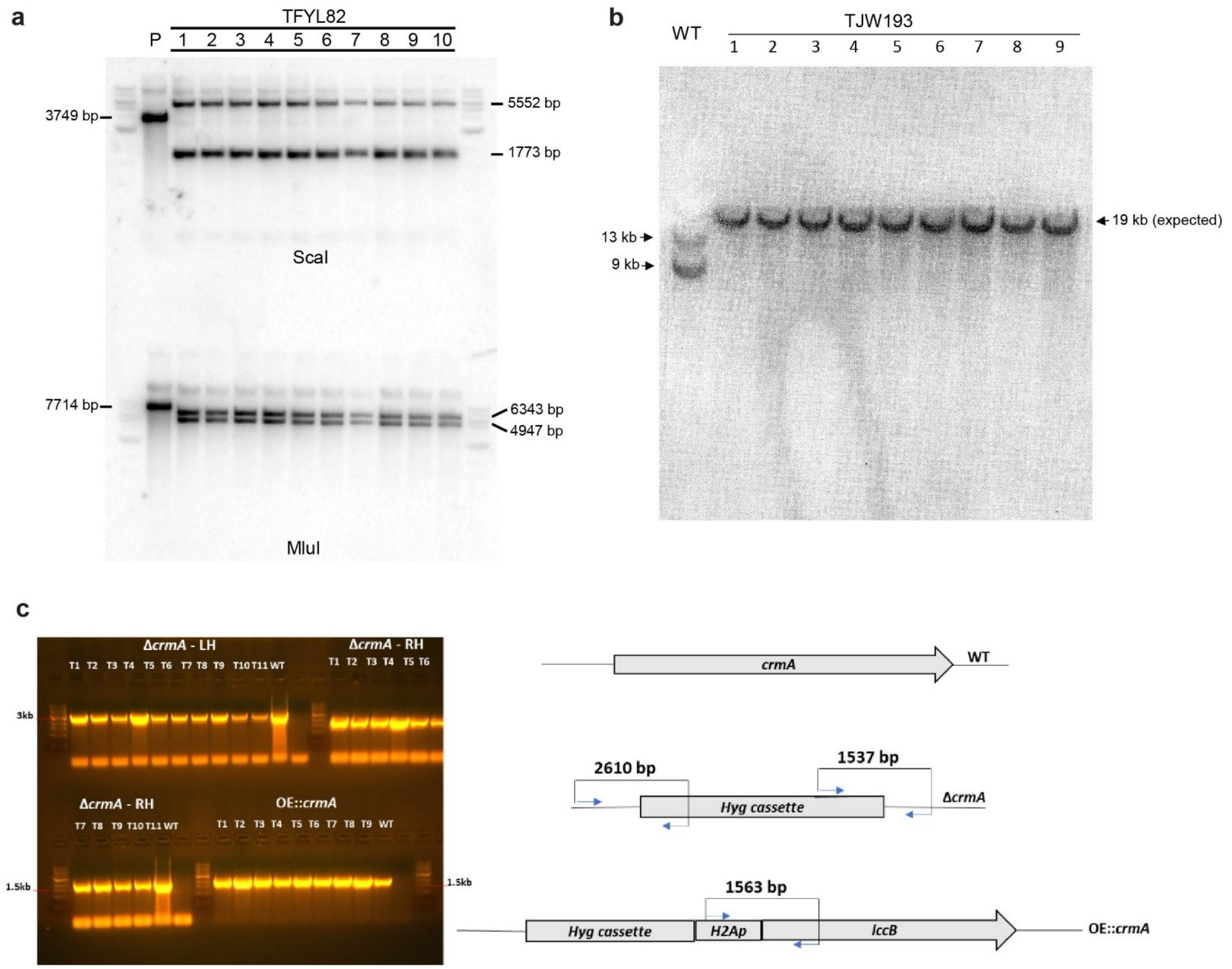

Supplementary Figure 8. Construction of overexpression and deletion mutants. **a,** Southern confirmation of *A. fumigatus crmA* overexpression (OE) mutants. Genomic DNA was digested by *Sca*I *and Mlu*I. Parent (P, 3749 bp), and OE (5552 and 1773 bp) for *Sca*I digestion; P (7714 bp), and OE (6343 and 4947 bp) for *Mlu*I digestion. TFYL82.2 was chosen for the subsequent experiments. **b**, Southern confirmation of *A. fumigatus crmBC* deletion mutants. Genomic DNA was digested by *Nde*I. WT (19 kb), and Δ*crmBC* (13 and 9 kb). TJW193.3 was chosen for the subsequent experiments. **c**, PCR confirmation of *P. expansum crmA* mutants. Expected PCR products; for Δ*crmA* LH (2610 bp) and RH (1537 bp); for OE::*crmA* (1563 bp). For all transformations, DNA was extracted from each transformant and PCR primers were used to determine if the correct genetic manipulation was achieved. PCR results were confirmed for each transformed strain with Southern blots.

2. Supplementary Tables

Supplementary Table 1. HPLC-HRMS data for compounds **1-15**

Compound	HRESI(+/-) observed (*m/z*)	Ion	Calculated ion formula	Calculated *m/z*	Retention time (min)	Yield of compound (per L culture)[a]
1	384.2278	$[M+H]^+$	$C_{22}H_{30}O_3N_3^+$	384.2282	7.8	~ 0.2-0.3 mg
2	241.1696	$[M+H]^+$	$C_{16}H_{21}N_2^+$	241.1699	7.8, 8.1[b]	
3	332.1306	$[M+Na]^+$	$C_{12}H_{23}O_8NNa^+$	332.1316	5.0	
4	144.0667	$[M-H]^-$	$C_6H_{10}O_3N^-$	144.0666	5.5	~0.1mg
5	126.0558	$[M-H]^-$	$C_6H_8O_2N^-$	126.0561	6.9	
10	374.1411	$[M+Na]^+$	$C_{14}H_{25}O_9NNa^+$	374.1422	5.9	
11	416.1519	$[M+Na]^+$	$C_{16}H_{27}O_{10}NNa^+$	416.1527	7.0-7.7	
12	459.1944	$[M+Na]^+$	$C_{18}H_{32}O_{10}N_2Na^+$	459.1949	7.2	
13	501.2054	$[M+Na]^+$	$C_{20}H_{34}O_{11}N_2Na^+$	501.2055	8.0-8.6	
14	305.1203	$[M+Na]^+$	$C_{11}H_{22}O_8Na^+$	305.1207	3.5	
15	305.1203	$[M+Na]^+$	$C_{11}H_{22}O_8Na^+$	305.1207	3.5	
Fumivaline B	400.2228	$[M+H]^+$	$C_{22}H_{30}O_4N_3^+$	400.2231	5.2	
Fumicicolin B	330.1157	$[M+Na]^+$	$C_{12}H_{21}O_8NNa^+$	330.1159	5.0-5.5	

[a]Numbers indicate estimated production of each compound based on peak areas for the molecular ions in fresh extracts compared to synthetic or isolated samples.
[b]Numbers indicate retention times for festuclavine and pyroclavine.

Supplementary Table 2. Calculation of relative abundances of hydrogen isotopes in extracts from the deuterium labeling experiment

Solvent	Molecular weight [g/mol]	Volume used [mL]	Density [g/mL]	Amount [mol]	Contribution to corres-ponding hydrogen isotope abundance in the extracts*
Water (H_2O)	18.0	5.0	0.997	5 x 0.9997/18 = 0.277	0.277 x 2 = 0.554 (H)
Deuterium oxide (D_2O)	20.0	1.5	1.110	1.5 x 1.110/20 = 0.083	0.083 x 2 = 0.166 (D)
Methanol-d_1 (CH_3OD)	33.0	3.5	0.813	3.5 x 0.813/33 = 0.086	0.086 x 1 = 0.086 (D)

*As a result, the ratio of protium to deuterium in the extract solvent is 0.554/(0.166+0.086) = 2.2, corresponding to a deuteration level of (0.166+0.086)/(0.166+0.086+0.554) = 31.3%.

Supplementary Table 3. Accession number, putative functions, and sequence identity with the ThiJ/PfpI family protein in *Pseudomonas putida,* of homologous proteins in *A. fumigatus*

E value	Identity (%)	Accession number in *A. fumigatus*	Putative function
6e-24	32.86%	XP_753233.1	ThiJ/PfpI family protein
3e-15	31.53%	XP_746317.2	ThiJ/PfpI family protein
2e-14	35.94%	XP_748565.1	ThiJ/PfpI transcriptional regulator
4e-11	32.30%	XP_754934.1	ThiJ/PfpI family protein
2e-09	26.03%	XP_748075.1	ThiJ/PfpI family protein

Supplementary Table 4. Accession numbers and sequence identity of the CrmA and the ergot alkaloid synthases in *A. fumigatus* with homologous proteins in *P. commune*

Gene name in *A. fumigatus*	Gene name in *Claviceps purpurea*	*A. fumigatus*	*P. commune* (*P. commune 162_3FA)*[1]
crmA	*crmA*	Q4WYN6.2	Pc.00g057360 (60%)
fgaOx3	*easA*	XP_756133.1	AFM84626.1 (64%)
fgaFS	*easG*	XP_756134.1	AFM84625.1 (61%)
fgaDH	*easD*	XP_756137.1	Pc.00g221860 (73%)
fgaCat	*easC*	XP_756140.1	Pc.00g221830 (64%)
fgaPT2	*dmaW*	XP_756141.1	Pc.00g221850 (63%)
fagOX1	*easE*	XP_756142.1	Pc.00g221840.1 (49%)
gaMT	*easF*	XP_756143.1	Pc.00g221840.2 (61%)

Supplementary Table 5. Antimicrobial susceptibility of various bacteria and yeast against extracts from different *A. fumigatus* strains grown in normal copper and copper devoid media.

Microorganism	*A.fumigatus* strain	Copper condition	Zone of inhibition (in cm, 3 replicates)
C. auris	Wildtype	-Cu	nd*, 1.1, 1.8
	Δ*crmA*		nd, nd, nd
	OE::*crmA*		1.7, 1.8, 1.9
	Wildtype	+Cu	nd, nd, nd
	Δ*crmA*		nd, nd, nd
	OE::*crmA*		nd, nd, nd
C. albicans	Wildtype	-Cu	nd, nd, 1.7
	Δ*crmA*		nd, nd, nd
	OE::*crmA*		1.7, 1.8, 1.7
	Wildtype	+Cu	nd, nd, nd
	Δ*crmA*		nd, nd, nd
	OE::*crmA*		nd, nd, nd
S. aureus	Wildtype	-Cu	nd, 1.3, 1.6
	Δ*crmA*		nd, nd, nd
	OE::*crmA*		nd, 1.6, 1.5
	Wildtype	+Cu	nd, nd, nd
	Δ*crmA*		nd, nd, nd
	OE::*crmA*		nd, nd, nd
P. aeruginosa	Wildtype	-Cu	nd, nd, nd
	Δ*crmA*		nd, nd, nd
	OE::*crmA*		nd, nd, nd
	Wildtype	+Cu	nd, nd, nd
	Δ*crmA*		nd, nd, nd
	OE::*crmA*		nd, nd, nd
A.brassicicola	Wildtype	-Cu	nd, 1.1, 2.4
	Δ*crmA*		nd, nd, nd
	OE::*crmA*		2.1, 2.5, 2.7
	Wildtype	+Cu	nd, nd, nd
	Δ*crmA*		nd, nd, nd
	OE::*crmA*		nd, nd, nd
P.expansum	Wildtype	-Cu	nd, 0.9, 1.1
	Δ*crmA*		nd, nd, nd
	OE::*crmA*		1.1, 1.4, 1.2
	Wildtype	+Cu	nd, nd, nd
	Δ*crmA*		nd, nd, nd
	OE::*crmA*		nd, nd, nd
E.coli	Wildtype	-Cu	nd, 1.0, 1.4
	Δ*crmA*		nd, nd, nd
	OE::*crmA*		1.4, 1.7, 1.5
	Wildtype	+Cu	nd, nd, nd
	Δ*crmA*		nd, nd, nd
	OE::*crmA*		nd, nd, nd
L.monocytogenes	Wildtype	-Cu	nd, 2.1, 2.2
	Δ*crmA*		nd, nd, nd
	OE::*crmA*		2.4, 2.6, 2.4
	Wildtype	+Cu	nd, nd, nd
	Δ*crmA*		nd, nd, nd
	OE::*crmA*		nd, nd, nd

*Not detected.

Supplementary Table 6. Fungal and bacterial strains used in this study

Name	Genotype	Reference
<u>*A. fumigatus*</u>		
TFYL45	*ΔnkuA::mluc; pyrG1; argB1*	Ref[2]
TFYL81	*fumipyrG; fumiargB; ΔnkuA::mluc; pyrG1; argB1*	Ref[2]
TFYL80.1	*fumiargB; ΔnkuA::mluc; pyrG1; argB1*	Ref[2]
TFYL93	*ΔcrmA::fumiargB; fumipyrG; ΔnkuA::mluc; pyrG1; argB1*	Ref[2]
TFYL89.1	*ΔcrmCD::fumiargB; ΔnkuA::mluc; fumipyrG; pyrG1; argB1*	Ref[2]
TFYL82.2	*parapyrG::gpdA(p)::crmA; pyrG1; argB1*	This study
TJW193.3	*ΔcrmBC::parapyrG; fumiargB; ΔnkuA::mluc; pyrG1; argB1*	This study
TTC32.1	*ΔdmaW::parapyrG; fumiargB; ΔnkuA; pyrG1; argB1*	Ref[3]
<u>*Penicillium expansum*</u>		
TJT14.1	*Δku*	Ref[4]
TCG7	*Δku; H2A(p)::062980::hygB*	This study
TCG8	*Δku; Δ062980::hygB*	This study
Other fungi		
P. commune	WT	Ref[1]
Cochliobolus heterostrophus	WT race O strain C5	Ref[5]
C. heterostrophus	*ΔcrmA*	This study
Alternaria brassicicola ATCC96836	WT	Strain from Dr.Mehdi Kabbage, UW-Madison
Candida albicans SC5314	WT	Strain from Dr.David Andes, UW-Madison
Candida auris B11804	WT	Strain from Dr.David Andes, UW-Madison
Bacteria		
Listeria monocytogenes 10403S	mCherry::ChlR (chromosomal integration)	Ref[6]
Escherichia coli UTI89	GFP (pGEN-GFP LVA)::AmpR	Ref[7]
Staphylococcus aureus (MRSA) FPR3757	dsRed::chlR (β-lactamase deactivated)	Strain from Dr. J D Sauer, UW-Madison
Pseudomonas aeruginosa PaO1	GFP (pSMC2)::CarbenicillinR	Ref[8]

Supplementary Table 7. PCR primers for this study

Name	Sequence (5'-3')	Use
Aspergillus fumigatus work		
crmDkoBCF	CTGGGTTGGTTATGTGTTGACGG	ΔcrmBC
crmDkoBCR	CGATATCAAGCTATCGATACCTCGACTCAT	ΔcrmBC
	GCACCACAACGACCACAGCAACGGGATGC	ΔcrmBC
ParapyrGF	GAGTCGAGGTATCGATAGCTTG	ΔcrmBC
ParapyrGR	ATTCGACAATCGGAGAGGCTGC	ΔcrmBC
crmAkoBCF	GTCGCTGCAGCCTCTCCGATTGTCGAATA	ΔcrmBC
	TGCATCACTCCGACAGAATGTTTCACAAGG	ΔcrmBC
crmAkoBCR	AGCCAGTCTTGGTGTTCAGCAGGG	ΔcrmBC
OE3G13690 5'F FOR	GTCTTGCTCTTCGGCCGC	CrmA OE
OE3G13690 5'F REV	ATTCGCCCTATAGTGAGTCGTATTAC<br>GGGTACTGCACTGTGATGGGGCC	CrmA OE
OE3G13690 3'F FOR	CAGCTACCCCGCTTGAGCAGACATCAC<br>CATGCATCACTCCGACAGAATGTTTC	CrmA OE
OE3G13690 3'F REV	GATGAGGAGGTGGAAGCAGCAC	CrmA OE
OE3G13690 nest FOR	GGCGAAGTGAGGATTCGGAG	CrmA OE
OE3G13690 nest REV	GATGAGGAGGTGGAAGCAGCAC	CrmA OE
Extended T7 FOR	CGTAATACGACTCACTATAGGG	CrmA OE
gpdA(p) fusion REV	GGTGATGTCTGCTCAAG	CrmA OE
fumargBF	AGCCGAACGTGGCAATAGAACG	Complementation
fumargBR	TCTGGGACTGACAGACCTTAGC	Complementation
Penicillium expansum work		
PEX2_062980-LH-F	TAATGCCAACTTTGTACAAAAAAGCAG<br>GCTCCAACCCACCAACCTTGGAA	Deletion/OE
PEX2_062980-LH-F-int	CCTTGGAATGGGCTGAGTTTTAG	Deletion/OE
PEX2_062980-LH-R	TATTGACCTATAGGACCTGAGTGAT<br>GCGGACTATATCTGGCCGGTAGG	Deletion/OE
PEX2_0629800OE_ORF_F	TACAACATTTTAACCACTTAATCA<br>GACAAAATGGCTTCGCTGGACCC	OE
PEX2_062980-ORF-Rin	GCTGTCTTGGCTGAACTCG	OE
PEX2_062980-ORF-R	AATGCCAACTTTGTACAAGAAAGCT<br>GGGTCGATACGGTGCAGCCTTTCTT	OE
PEX2_062980-KO-RH-F	GTTGAGCATAATATGGTCCATCTAGT<br>GCCGTTAGACGGCGAACTCCA	Deletion
PEX2_062980-KO-RH-Rin	CGCCACGAAGATCATCGAC	Deletion
PEX2_062980-KO-RH-R	AATGCCAACTTTGTACAAGAAAGCTGGG<br>TCGAGGTCATCGATTCGGACAG	Deletion
PEX2_062980-checkLH-F	CCGCAAGATCACATCAACCC	Confirmation
PEX2_062980-checkLH-R	GCAGTTGGTTCTTCGAGTCGA	Confirmation
PEX2_062980-checkRH-F	GCCGATGCAAAGTGCCGATA	Confirmation
PEX2_062980-checkRH-R	GGTTCATGATCAACAAGCCCGGGA	Confirmation
PEX2_062980-check-OE-R	GACATAAGCATTGACATCAATG	Confirmation
PEX2_062980-check-OE-F	GGAGCACTGGACCATGATCT	Confirmation
Cochliobolus heterostrophus work		

*Ch*C5_1220253-UpF	GACGCCATGCCTACTAATCC	Confirmation
*Ch*C5_1220253-DownR	GTCCACGCCATGGTAGTAGC	Confirmation
*Ch*C5_1220253-intF	GGCAGTGCTAGAGGCAACTC	Confirmation
*Ch*C5_1220253-intR	TGCGCAGATAAACTTTGACG	Confirmation
*Ch*C5_1220253-FP1	CGATCGTCTCACTGTGCTTG	Deletion
*Ch*C5_1220253-RP1(M13R)	tcctgtgtgaaattgttatccgct<br>AGTATGTGCCCAGGCTTCTG	Deletion
*Ch*C5_1220253-FP2(M13F)	gtcgtgactgggaaaaccctggcg<br>TCAACTTCCATTTCAGACAGTTTC	Deletion
*Ch*C5_1220253-RP2	CGACTGTTCCGAACTGACAAC	Deletion
M13R	AGCGGATAACAATTTCACACAGGA	Deletion
M13F	CGCCAGGGTTTTCCCAGTCACGAC	Deletion
NLC37	GGATGCCTCCGCTCGAAGTA	Deletion
NLC38	CGTTGCAAGACCTGCCTGAA	Deletion

3. Supplementary Methods

Synthesis of fumicicolin A (3) and heterocicolin C (12)

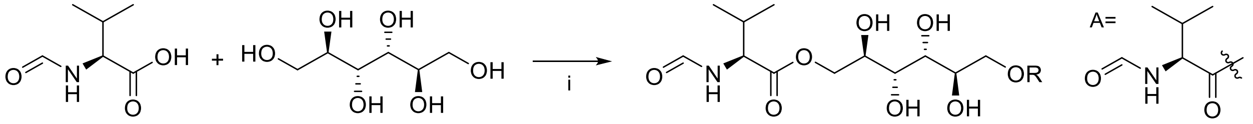

4 D-Mannitol **3** R=H, **12** R=A

(i) To a stirred solution of *N*-formylvaline (TCI America, 145 mg, 1 mmol), 4-dimethylaminopyridine (488 mg, 4 mmol, 4.0 equiv.), and 1-ethyl-3-(3-dimethylaminopropyl) carbodiimide hydrochloride (383 mg, 2 mmol, 2.0 equiv.) in 2 mL of *N*,*N*-dimethylformamide was added D-mannitol (Sigma, 200 mg, 1.1 mmol, 1.1 equiv.). The reaction mixture was stirred at room temperature for 2 hr. The resulting mixture was concentrated *in vacuo*. Purification by flash chromatography on a reverse-phase column (C18) using a gradient of 0-50 % acetonitrile in 0.1 % acetic acid afforded fumicicolin A (**3**, 150 mg, 45 %) and heterocicolin C (**12**, 150 mg, 45 %).

Fumicicolin A (**3**): HRMS (ESI) *m/z*: $[M+Na]^+$ calcd for $C_{12}H_{23}NO_8Na^+$ 332.1316; found 332.1311.

Heterocicolin C (**12**): HRMS (ESI) *m/z*: $[M+Na]^+$ calcd for $C_{18}H_{32}N_2O_{10}Na^+$ 459.1949; found 459.1944. See next section for NMR spectroscopic data and spectra.

Synthesis of (*S*)-2-isocyanoisovaleric acid (5)

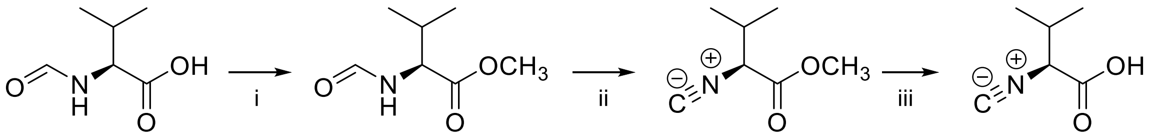

4 **5a** **5b** **5**

(i) To a stirred solution of *N*-formylvaline (TCI America, 145 mg, 1 mmol) in 1 mL of methanol was added (trimethylsilyl)diazomethane (*ca*. 0.6 mol/L solution in hexane, 2 mL, 1.2 mmol, 1.2 equiv.). The reaction mixture was stirred at room temperature for 24 hr, then quenched with the dropwise addition of acetic acid until the yellow color of the reaction mixture disappeared and bubbling ceased. The resulting mixture was concentrated *in vacuo*. Purification by flash column chromatography on silica using a gradient of 0-10% methanol in dichloromethane afforded *N*-formylvaline methyl ester (**5a**, 155 mg, 97 %). HRMS (ESI) *m/z*: $[M-H]^-$ calcd for $C_7H_{12}NO_3^-$ 158.0823; found 158.0822. ^1H NMR, 600 MHz, chloroform-*d*: δ (ppm) Major rotamer: 8.24 (s, 1H), 6.30 (m, 1H) 4.64 (dd, *J* = 9.0, 4.8 Hz, 1H), 3.74 (s, 3H), 2.18 (m, 1H), 0.95 (d, *J* = 6.7 Hz, 3H), 0.90 (d, *J* = 6.6 Hz, 3H). Minor rotamer: 7.98 (d, *J* = 11.8 Hz, 1H), 6.30 (m, 1H), 4.51 (dd, *J* = 10.2, 5.2 Hz, 1H), 3.76 (s, 3H), 2.32 (m, 1H), 0.97 (d, *J* = 6.6 Hz, 3H), 0.89 (m, 3H).

(ii) To a stirred solution of *N*-formylvaline methyl ester (**5a**, 143 mg, 0.9 mmol) in 9 mL dichloromethane was added triethylamine (626 µL, 4.5 mmol, 5 equiv.), followed by the dropwise addition of phosphoryl chloride (103 µL, 1.1 mmol, 1.2 eq) at -20 °C. The reaction mixture was stirred for 4 hr, then quenched with the dropwise addition of water (0.9 mL) at -20 °C. The organics were extracted three times with dichloromethane. Combined organics were washed with brine, dried with sodium sulfate, filtered, and concentrated *in vacuo*. Purification by

flash column chromatography on silica using a gradient of 0-10% methanol in dichloromethane afforded (*S*)-2-isocyanoisovaleric acid methyl ester (**5b**, 95 mg, 67 %).

HRMS (ESI) *m/z*: $[M-H]^-$ calcd for $C_7H_{10}NO_2^-$ 140.0717; found 140.0716.

^{1}H NMR, 600 MHz, methanol-d_4: δ (ppm) 4.51 (d, *J* = 4.0 Hz, 1H), 3.79 (s, 3H), 2.32 (m, 1H), 1.08 (d, *J* = 6.7 Hz, 3H), 0.95 (d, *J* = 6.7 Hz, 3H).

(iii) To a stirred solution of (*S*)-2-isocyanoisovaleric acid methyl ester (**5b**, 13 mg, 0.092 mmol) in 1 mL of 1,4-dioxane was added lithium hydroxide (2.4 mg, 0.1 mmol, 1.1 eq), followed by the addition of 1 mL of water. The reaction mixture was stirred at room temperature for 1 hr, and the organics were extracted with ethyl acetate (3x). The resulting solution was concentrated *in vacuo* to afford (*S*)-2-isocyanoisovaleric acid (**5**, 11 mg, 0.086 mmol, 94 %).

HRMS (ESI) *m/z*: $[M-H]^-$ calcd for $C_6H_8NO_2^-$ 126.0561; found 126.0558. See next section for NMR spectroscopic data and spectra.

Synthesis of heterocicolins E and F (14 and 15)

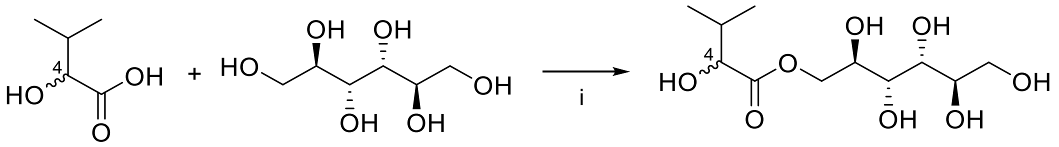

14a 4*R*
15a 4*S*

D-Mannitol

14 4*R*
15 4*S*

(i) To a stirred solution of (*R*)-(-)-2-hydroxyisovaleric acid (**14a**, VWR, 118 mg, 1 mmol), 4-dimethylaminopyridine (488 mg, 4 mmol, 4.0 equiv.), and 1-ethyl-3-(3-dimethylaminopropyl) carbodiimide hydrochloride (383 mg, 2 mmol, 2.0 equiv.) in 2 mL of *N*,*N*-dimethylformamide was added D-mannitol (Sigma, 200 mg, 1.1 mmol, 1.1 equiv.). The reaction mixture was stirred at room temperature for 2 hr. The resulting mixture was concentrated *in vacuo*. Purification by flash chromatography on a reverse phase column (C18) using a gradient of 0-5 % acetonitrile in 0.1 % acetic acid afforded heterocicolin E (**14**, 65 mg, 23 %). The same procedure was performed with (*S*)-(+)-2-hydroxyisovaleric acid (**15a**, VWR, 118 mg, 1 mmol) as an alternative to (*R*)-(-)-2-hydroxyisovaleric acid for synthesis of heterocicolin F (**15**, 65 mg, 23 %).

Heterocicolin E (**14**): HRMS (ESI) *m/z*: $[M+Na]^+$ calcd for $C_{11}H_{22}O_8Na^+$ 305.1207; found 305.1203. See next section for NMR spectroscopic data and spectra.

Heterocicolin F (**15**): HRMS (ESI) *m/z*: $[M+Na]^+$ calcd for $C_{11}H_{22}O_8Na^+$ 305.1207; found 305.1203. See next section for NMR spectroscopic data and spectra.

4. NMR Spectroscopic Data and Spectra

^{1}H (800 MHz) and ^{13}C (201 MHz) NMR spectroscopic data for fumivaline A (1) in methanol-d_4

Chemical shifts were referenced to δ(C$\underline{H}$D$_2$OD) = 3.31 and δ($^{13}\underline{C}$HD$_2$OD) = 49.0. ^{13}C chemical shifts were determined via HMBC and HSQC spectra. One-bond (^{13}C,^{1}H)-*J*-coupling constants were determined from the acquired HSQC spectra. (^{1}H,^{1}H)-*J*-coupling constants were determined from the ^{1}H or dqfCOSY spectra. HMBC correlations are from the proton(s) stated to the indicated ^{13}C atom.

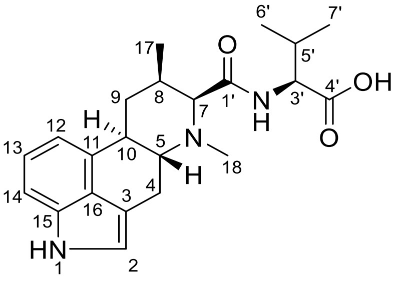

Fumivaline A (**1**)

No.	δc	Proton	$^1J_{CH}$	δH (J_{HH}[Hz])	HMBC	NOESY
2	117.9	2-H	182.5	6.90	4b	
3	109.9				2, 4b	
4	26.6	4-Ha (4α)	126.4	2.56 ($J_{4a,4b}$ = 13.5 Hz, $J_{4a,5}$ = 11.0 Hz)		10
		4-Hb (4β)		3.42 ($J_{4b,4a}$ = 13.5 Hz, $J_{4b,5}$ = 4.0 Hz)		
5	60.3	5-H	138.5	3.72 ($J_{5,4a}$ = 11.0 Hz, $J_{5,4b}$ = 4.0 Hz, $J_{5,10}$ = 11.0 Hz)	4b, 9ab, 18	9β
7	69.1	7-H	139.8	3.73 ($J_{7,8}$ = 5.5 Hz)	9ab, 17, 18	8, 17
8	32.8	8-H	124.6	2.31 ($J_{8,7}$ = 5.5 Hz, $J_{8,9a}$ = 3.0, $J_{8,9b}$ = 12.5 Hz, $J_{8,17}$ = 7.0 Hz)	9ab, 17	7, 10
9	30.3	9-Ha (9β)	130.0	1.93 ($J_{9a,9b}$ = 12.5 Hz, $J_{9a,8}$ = 3.0 Hz, $J_{9a,10}$ = 4.0 Hz)	17	5
		9-Hb (9α)		2.37 ($J_{9b,8}$ = 12.5 Hz, $J_{9b,9a}$ = 12.5 Hz, $J_{9b,10}$ = 11.0 Hz)	17	10
10	40.9	10-H	125.3	2.94 ($J_{10,5}$ = 11.0 Hz, $J_{10,9b}$ = 11.0 Hz, $J_{10,9a}$ = 4.0 Hz)	4b, 9ab, 12, 14	4α, 8, 9α
11	132.1				13	
12	111.9	12-H	156.1	6.82 ($J_{12,13}$ = 7.0 Hz)	14	
13	122.1	13-H	150.6	7.04 ($J_{13,12}$ = 7.0 Hz, $J_{13,14}$ = 8.0 Hz)		
14	108.3	14-H	161.5	7.12 ($J_{14,13}$ = 8.0 Hz)	12	
15	133.8				2, 13	
16	126.2				2, 4b, 12, 14	

17	17.4	17-H	126.1	1.15 ($J_{17,8}$ = 7.0 Hz)	9a	7, 9α
18	39.2	18-H	136.0	2.69		
1'	170.1				3'	
3'	58.7	3'-H	140.4	4.24 ($J_{3',5'}$ = 5.5 Hz)	5', 6', 7'	
4'	175.0				3'	
5'	30.9	5'-H	130.4	2.15 ($J_{5',3'}$ = 5.5 Hz, $J_{5',6'}$ = 7.0 Hz, $J_{5',7'}$ = 7.0 Hz)	3', 6', 7'	
6'	18.8	6'-H	125.5	0.96 ($J_{6',5'}$ = 7.0 Hz)	3', 5', 7'	
7'	17.2	7'-H	125.6	0.95 ($J_{7',5'}$ = 7.0 Hz)	3', 5', 6'	

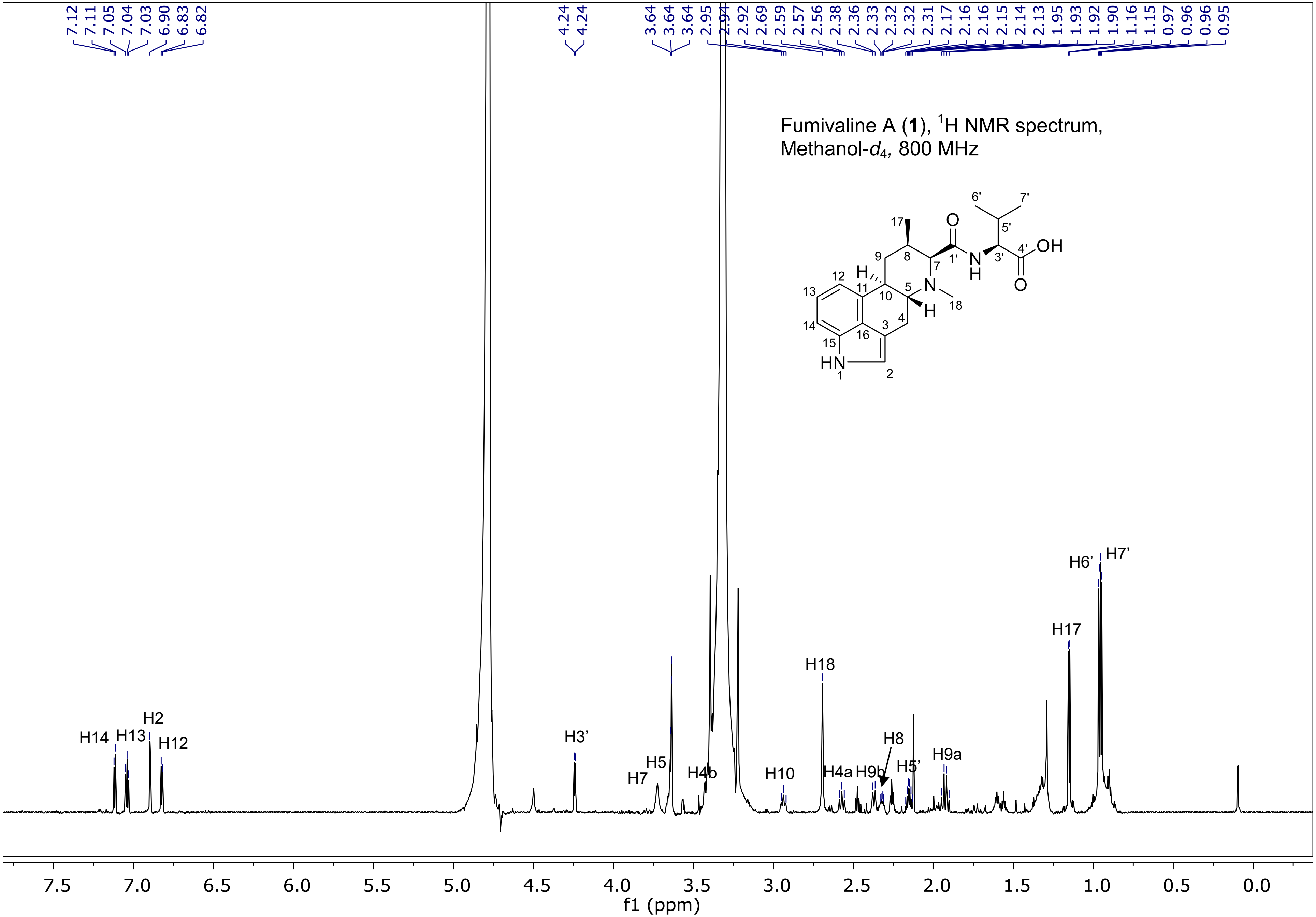
7.12 7.11 7.05 7.04 7.03 6.90 6.83 6.82
4.24 4.24
3.64 3.64 3.64 2.95 2.94 2.92 2.69 2.59 2.57 2.56 2.38 2.36 2.33 2.32 2.32 2.31 2.17 2.16 2.16 2.15 2.14 2.13 1.95 1.93 1.92 1.90 1.16 1.15 0.97 0.96 0.96 0.95
Fumivaline A (1), 1H NMR spectrum,
Methanol-d4, 800 MHz
H14
H13
H2
H12
H3'
H7
H5
H4b
H10
H18
H4a
H9b
H8
H5'
H9a
H17
H6'
H7'
7.5 7.0 6.5 6.0 5.5 5.0 4.5 4.0 3.5 3.0 2.5 2.0 1.5 1.0 0.5 0.0
f1 (ppm)

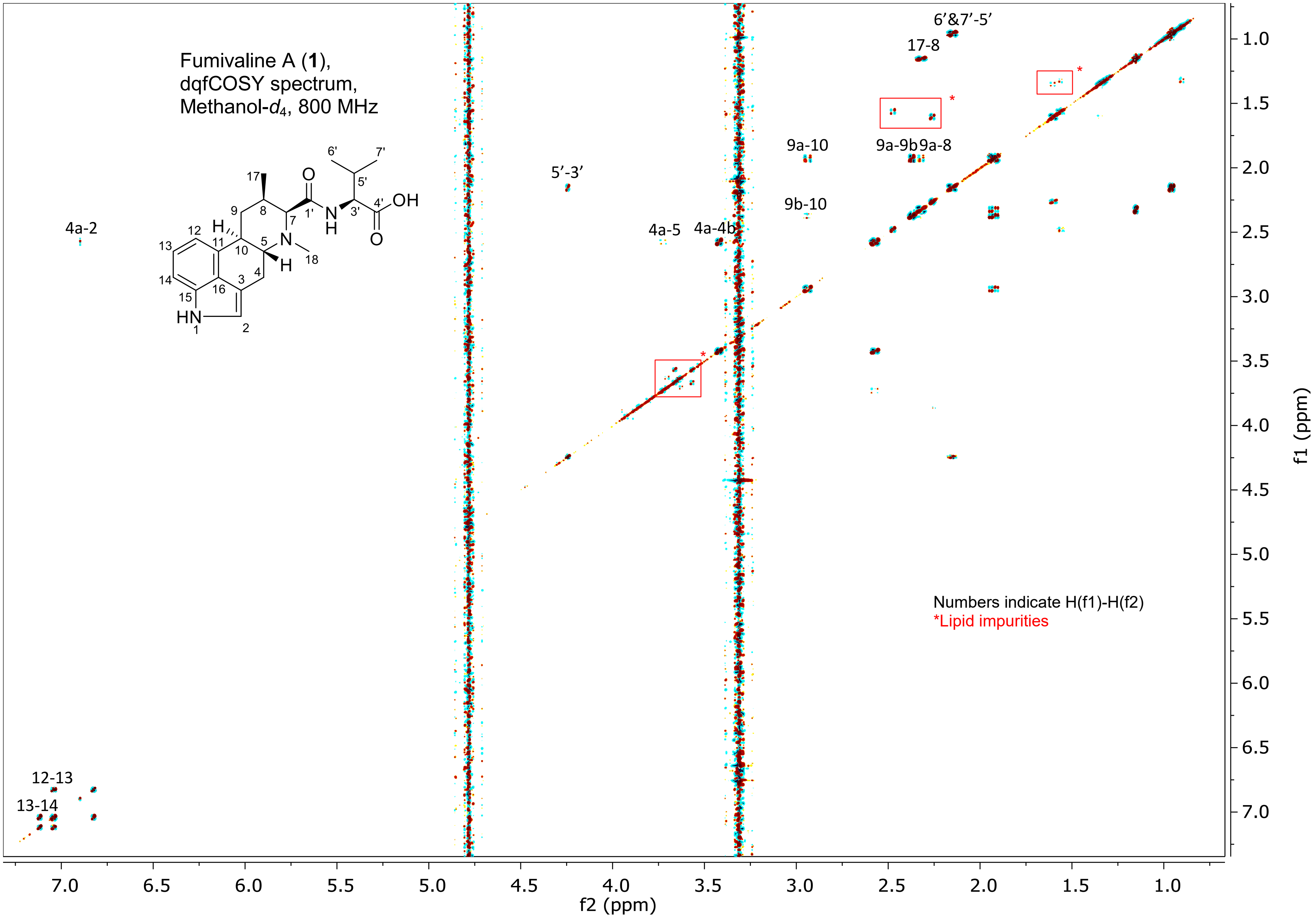
Fumivaline A (1),
dqfCOSY spectrum,
Methanol-d4, 800 MHz
Numbers indicate H(f1)-H(f2)
*Lipid impurities
6’&7’-5’
17-8
9a-10
9a-9b
9a-8
5’-3’
9b-10
4a-2
4a-5
4a-4b
12-13
13-14
f2 (ppm)
f1 (ppm)

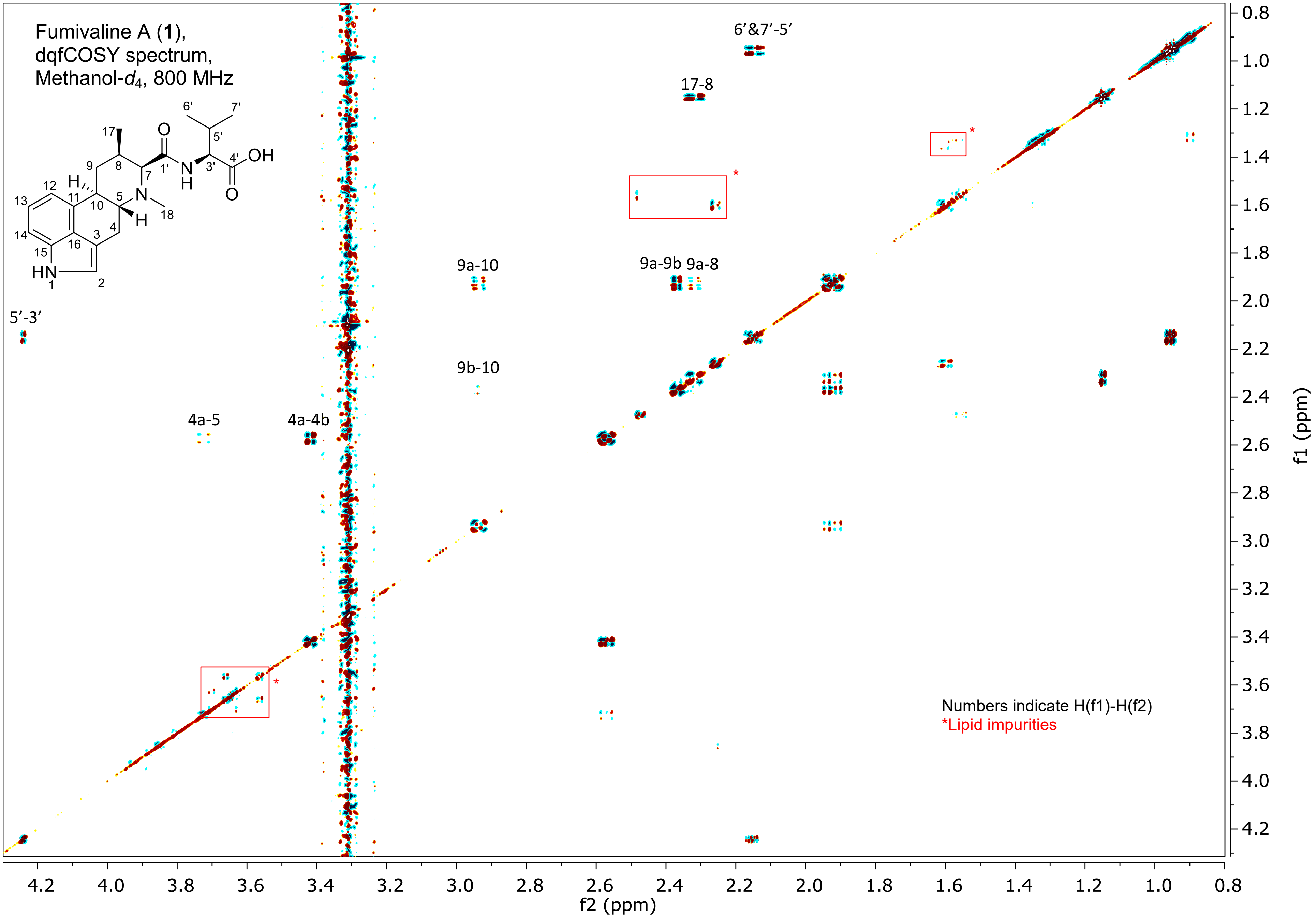

Fumivaline A (1),
dqfCOSY spectrum,
Methanol-d_4, 800 MHz
6'&7'-5'
17-8
9a-10
9a-9b 9a-8
5'-3'
9b-10
4a-5
4a-4b
Numbers indicate H(f1)-H(f2)
*Lipid impurities
f2 (ppm)
f1 (ppm)

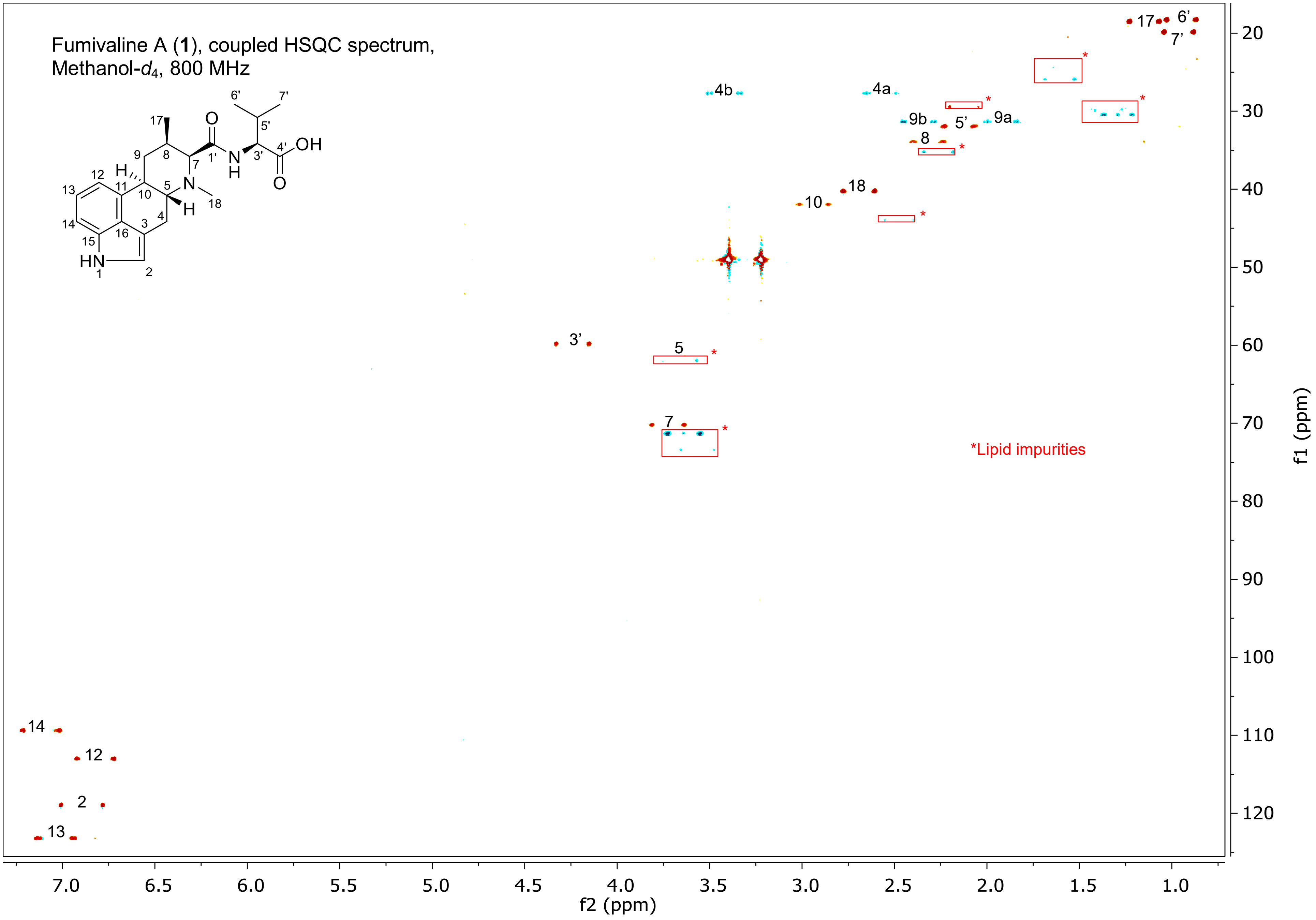

Fumivaline A (1), coupled HSQC spectrum,
Methanol-d_4, 800 MHz
*Lipid impurities
f2 (ppm)
f1 (ppm)

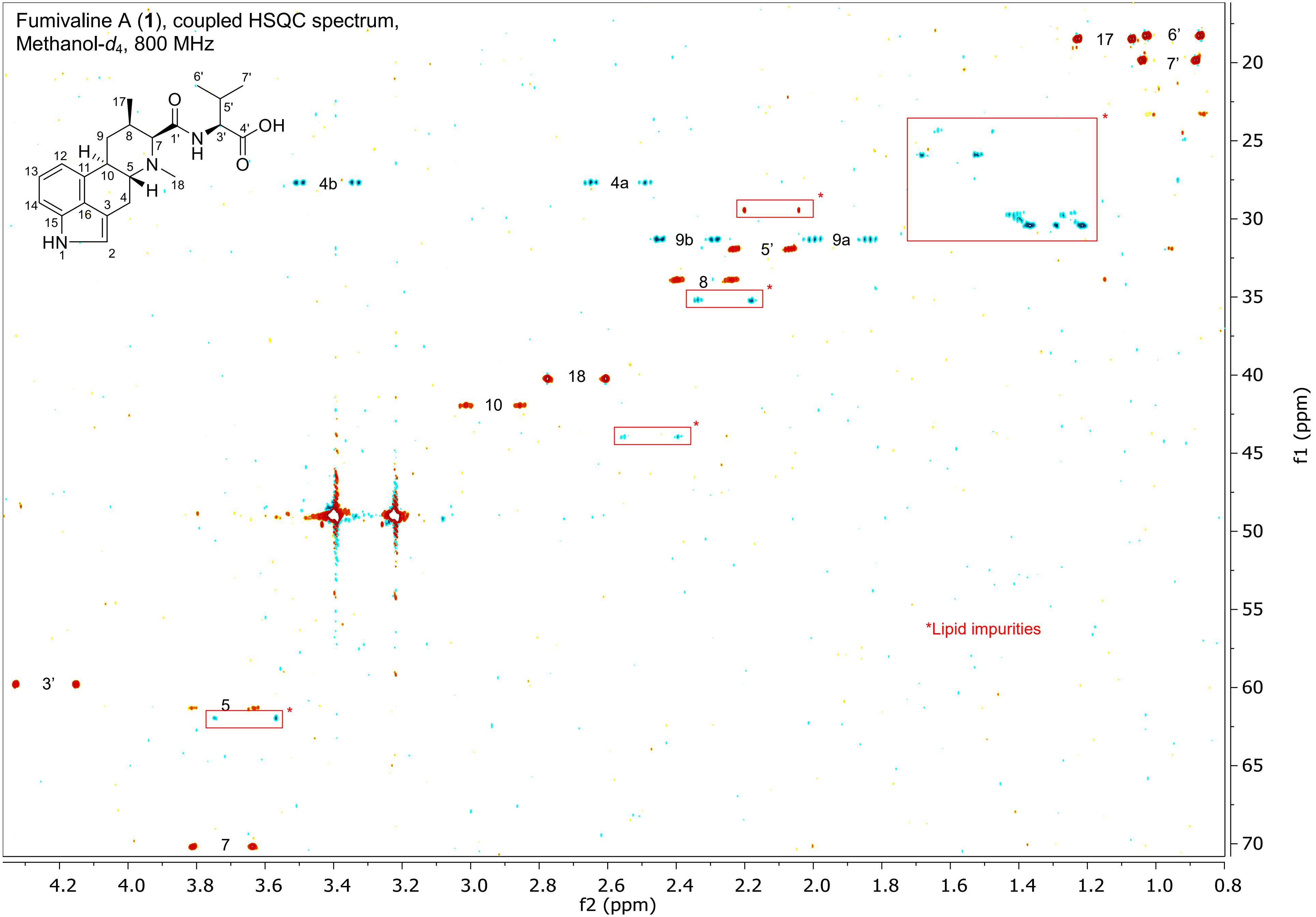
Fumivaline A (1), coupled HSQC spectrum,
Methanol-d_4, 800 MHz
17
6'
7'
*
4b
4a
9b
5'
9a
8
18
10
*Lipid impurities
3'
5
7
f2 (ppm)
f1 (ppm)

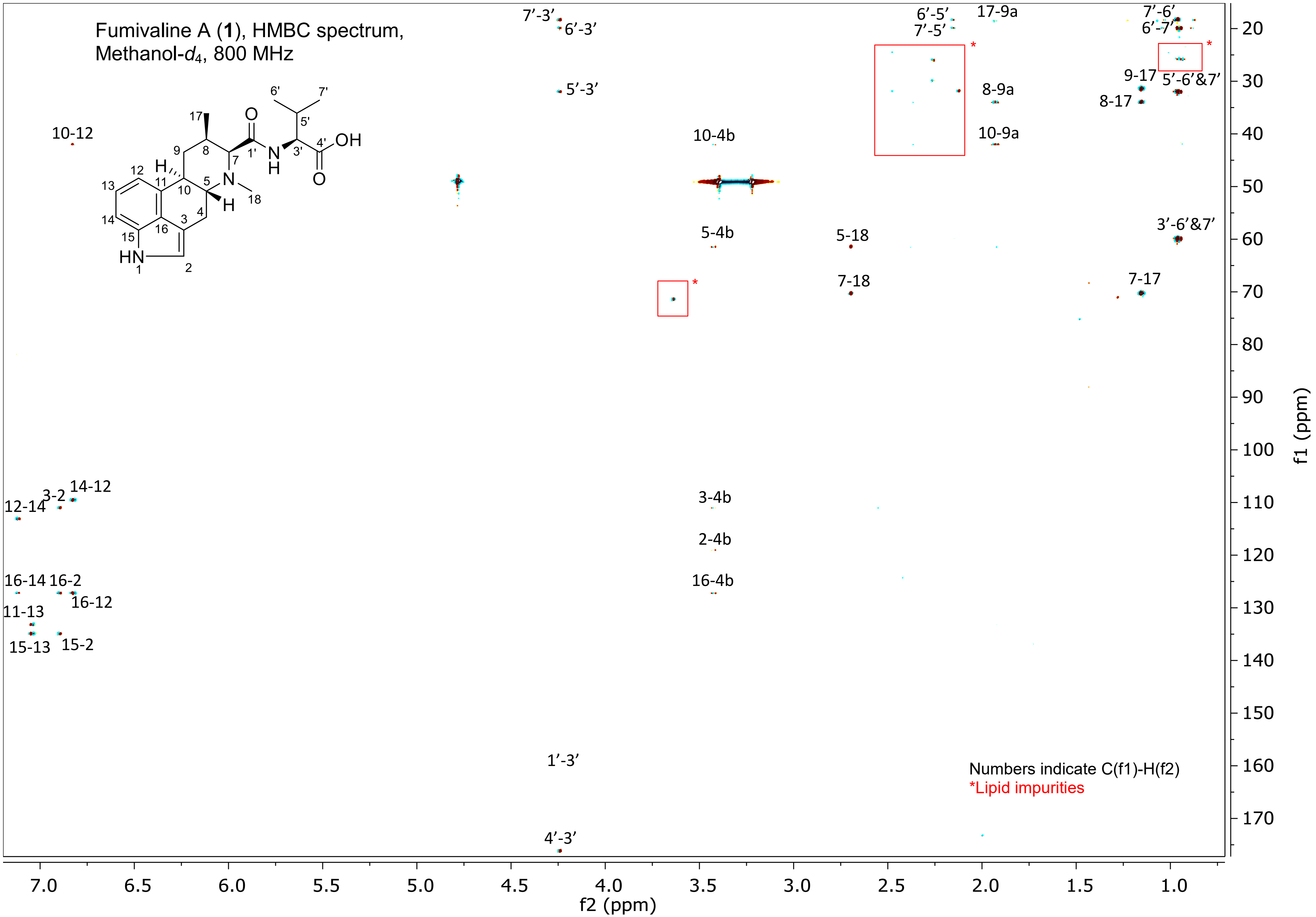
Fumivaline A (1), HMBC spectrum,
Methanol-d_4, 800 MHz
Numbers indicate C(f1)-H(f2)
*Lipid impurities
7’-3’
6’-3’
5’-3’
1’-3’
4’-3’
6’-5’
7’-5’
17-9a
7’-6’
6’-7’
9-17
5’-6’&7’
8-9a
8-17
10-9a
10-4b
10-12
5-4b
5-18
3’-6’&7’
7-18
7-17
3-4b
2-4b
16-4b
12-14
3-2
14-12
16-14
16-2
16-12
11-13
15-13
15-2
f2 (ppm)
f1 (ppm)

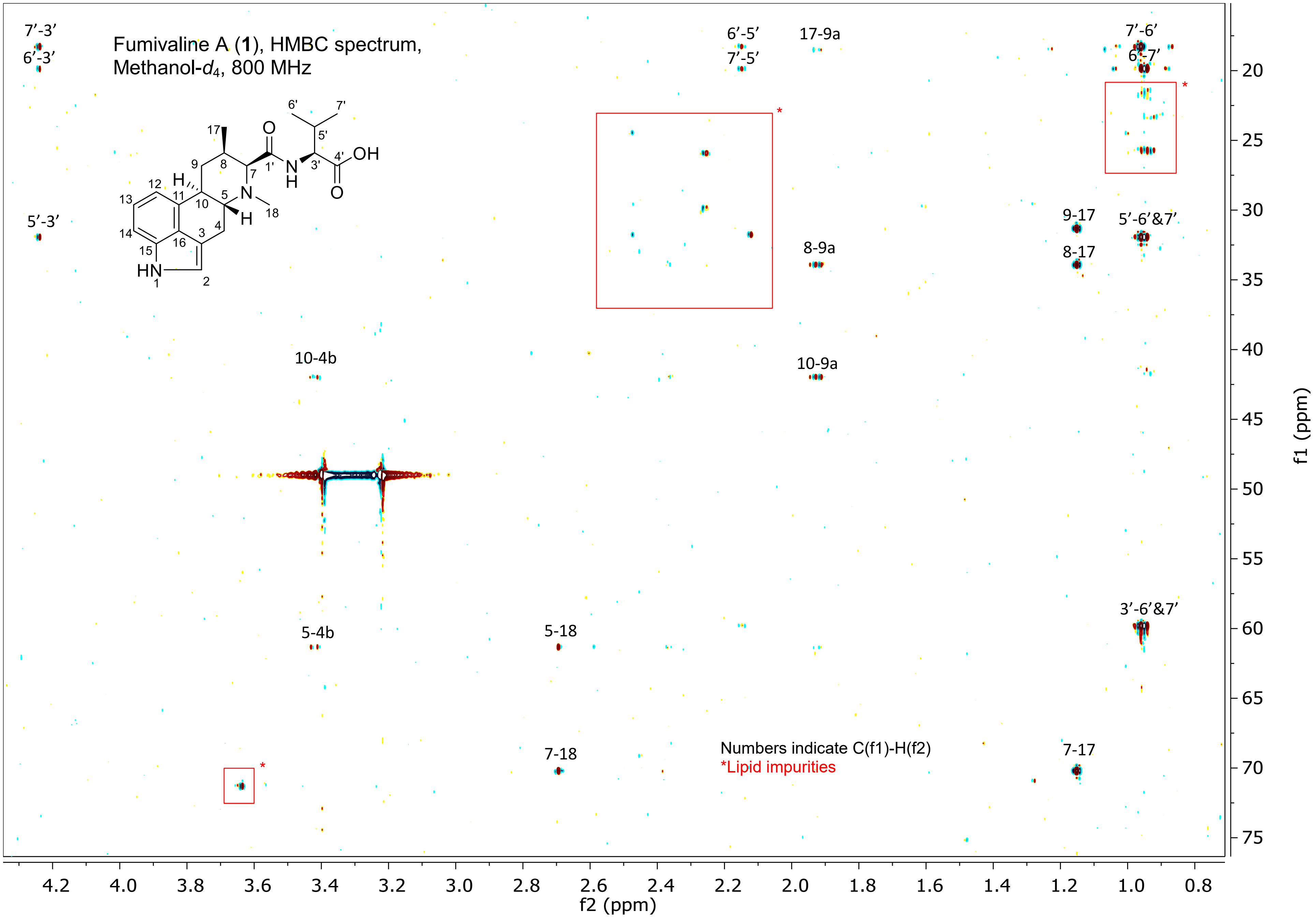
Fumivaline A (1), HMBC spectrum,
Methanol-d4, 800 MHz
7′-3′
6′-3′
5′-3′
6′-5′
7′-5′
17-9a
7′-6′
6′-7′
9-17
5′-6′&7′
8-17
8-9a
10-4b
10-9a
5-4b
5-18
3′-6′&7′
7-18
7-17
Numbers indicate C(f1)-H(f2)
*Lipid impurities
f2 (ppm)
f1 (ppm)

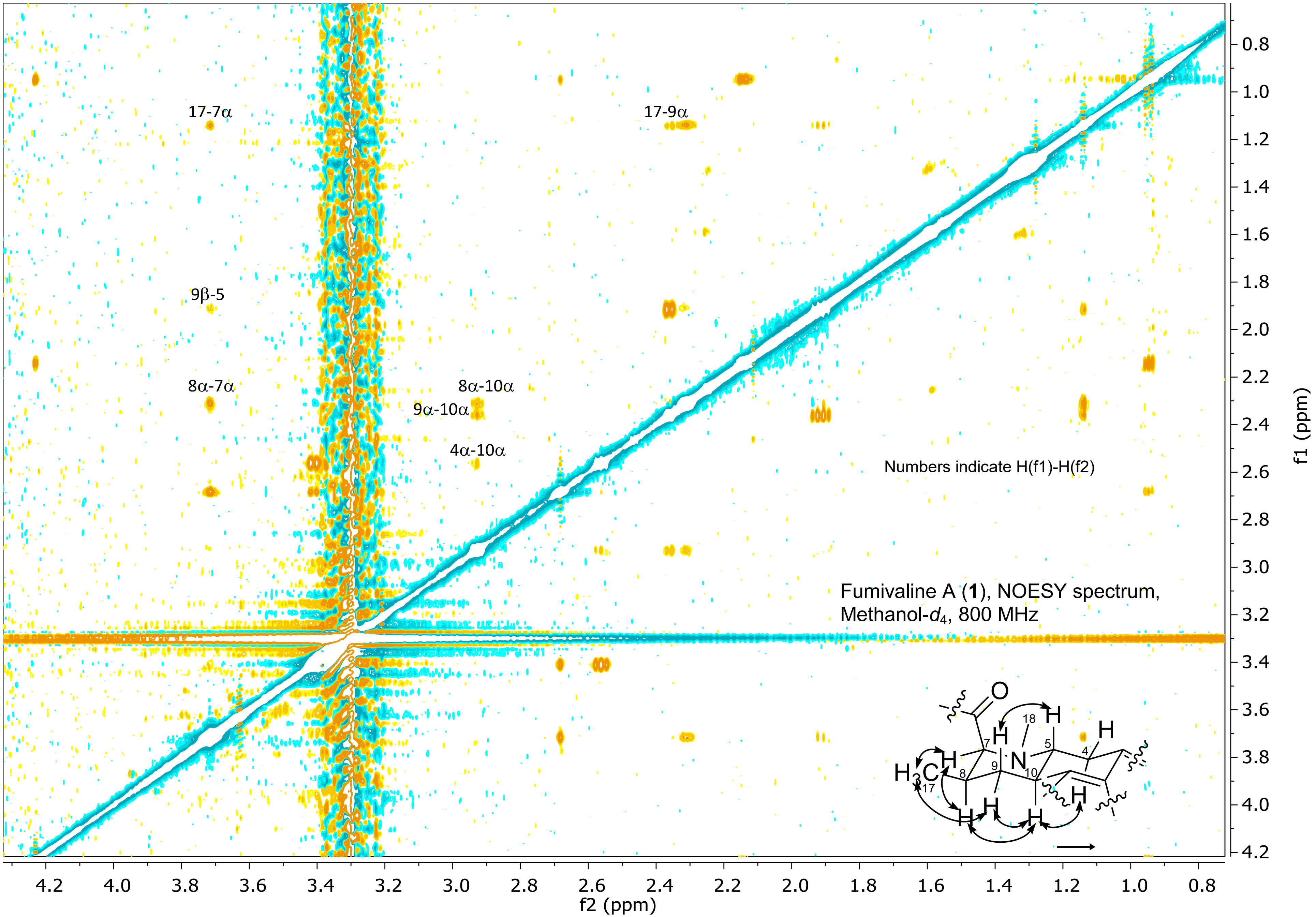

17-7α
17-9α
9β-5
8α-7α
8α-10α
9α-10α
4α-10α
Numbers indicate H(f1)-H(f2)
Fumivaline A (1), NOESY spectrum,
Methanol-d_4, 800 MHz
f1 (ppm)
f2 (ppm)

^{1}H (600 MHz) and ^{13}C (151 MHz) NMR spectroscopic data for fumicicolin A (3) in methanol-d_4

Chemical shifts were referenced to δ(C<u>H</u>D$_2$OD) = 3.31 and δ(^{13}<u>C</u>HD$_2$OD) = 49.0. (^{1}H,^{1}H)-*J*-coupling constants were determined from the ^{1}H or dqfCOSY spectra. HMBC correlations are from the proton(s) stated to the indicated ^{13}C atom.

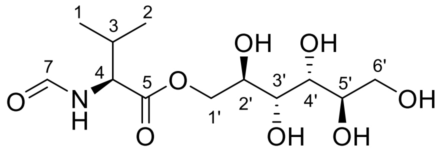

No.	δc	Proton	δH (J_{HH}[Hz])	HMBC
1	18.0	1-H	0.96 ($J_{1,3}$ = 6.8 Hz)	2, 3, 4
2	19.5	2-H	0.99 ($J_{2,3}$ = 6.8 Hz)	1, 3, 4
3	31.9	3-H	2.25 ($J_{3,1}$ = 6.8 Hz, $J_{3,2}$ = 6.8 Hz, $J_{3,4}$ = 5.5 Hz)	1, 2, 4
4	57.7	4-H	4.51 ($J_{4,3}$ = 5.7 Hz)	1, 2, 3, 7
5	172.7			4, 1'
7	163.8	7-H	8.14 (s)	4
1'	68.6	1'-Ha	4.45 ($J_{1'a,1'b}$ = 11.5 Hz, $J_{1'a,2'}$ = 3.5 Hz)-rotamer 1<br>4.43 ($J_{1'a,1'b}$ = 11.5 Hz, $J_{1'a,2'}$ = 3.5 Hz)-rotamer 2	3'
		1'-Hb	4.25 ($J_{1'b,1'a}$ = 11.5 Hz, $J_{1'b,2'}$ = 6.5 Hz)-rotamer 1<br>4.22 ($J_{1'b,1'a}$ = 11.5 Hz, $J_{1'b,2'}$ = 6.5 Hz)-rotamer 2	
2'	70.3	2'-H	3.90 ($J_{2',1'a}$ = 3.5 Hz, $J_{2',1'b}$ = 6.5 Hz, $J_{2',3'}$ = 8.5 Hz)	1b', 3', 4'
3'	71.0	3'-H	3.79 (m)	1a', 2'
4'	70.9	4'-H	3.79 (m)	5', 6'
5'	72.9	5'-H	3.69 ($J_{5',4'}$ = 8.5 Hz, $J_{5',6'a}$ = 4.0 Hz, $J_{5',6'b}$ = 6.5 Hz)	3', 4', 6'
6'	65.1	6'-Ha	3.81 ($J_{6'a,6'b}$ = 11.5 Hz, $J_{6'a,5'}$ = 4.0 Hz)	4', 5'
		6'-Hb	3.64 ($J_{6'b,6'a}$ = 11.5 Hz, $J_{6,5}$ = 6.5 Hz)	

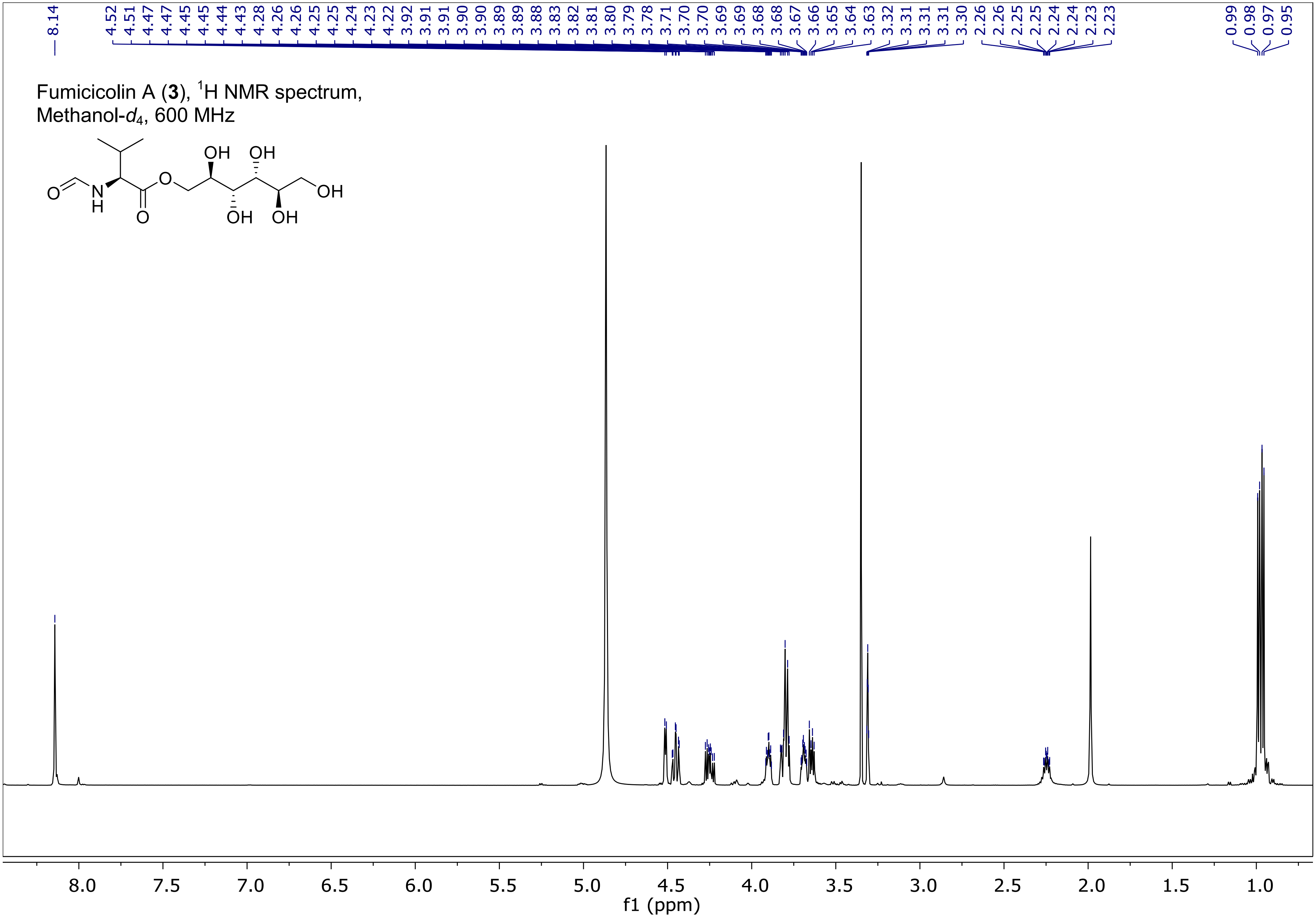
8.14
4.52 4.51 4.47 4.47 4.45 4.45 4.44 4.43 4.28 4.26 4.26 4.25 4.25 4.24 4.23 4.22 3.92 3.91 3.91 3.90 3.90 3.89 3.89 3.88 3.83 3.82 3.81 3.80 3.79 3.78 3.71 3.70 3.70 3.69 3.69 3.68 3.68 3.67 3.66 3.65 3.64 3.63 3.32 3.31 3.31 3.31 3.30
2.26 2.26 2.25 2.25 2.24 2.24 2.23 2.23
0.99 0.98 0.97 0.95
Fumicicolin A (3), 1H NMR spectrum,
Methanol-d4, 600 MHz
OH OH
O N H O O OH
OH OH
8.0 7.5 7.0 6.5 6.0 5.5 5.0 4.5 4.0 3.5 3.0 2.5 2.0 1.5 1.0
f1 (ppm)

Fumicicolin A (**3**), ^{13}C NMR spectrum, Methanol-d_4, 151 MHz

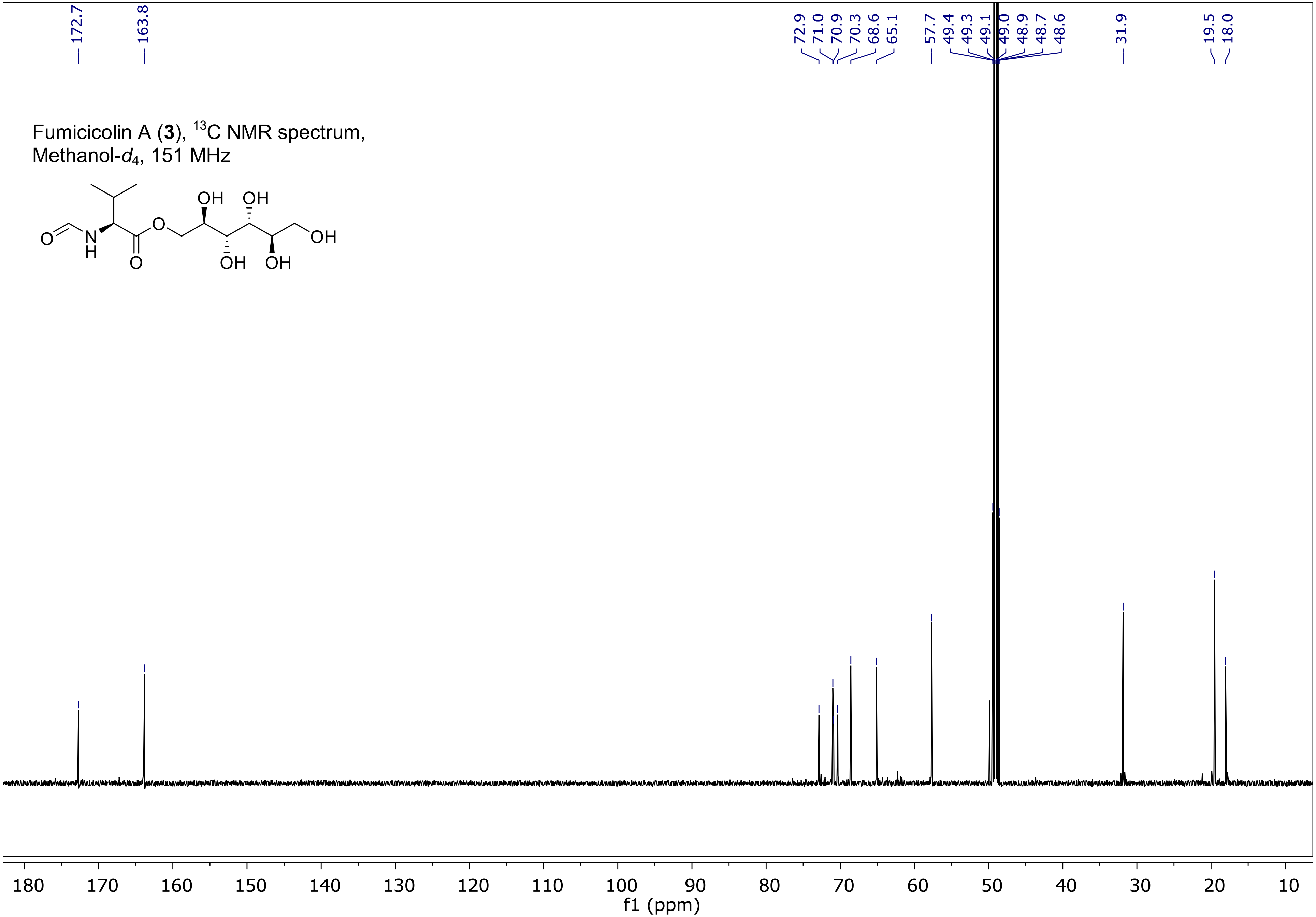

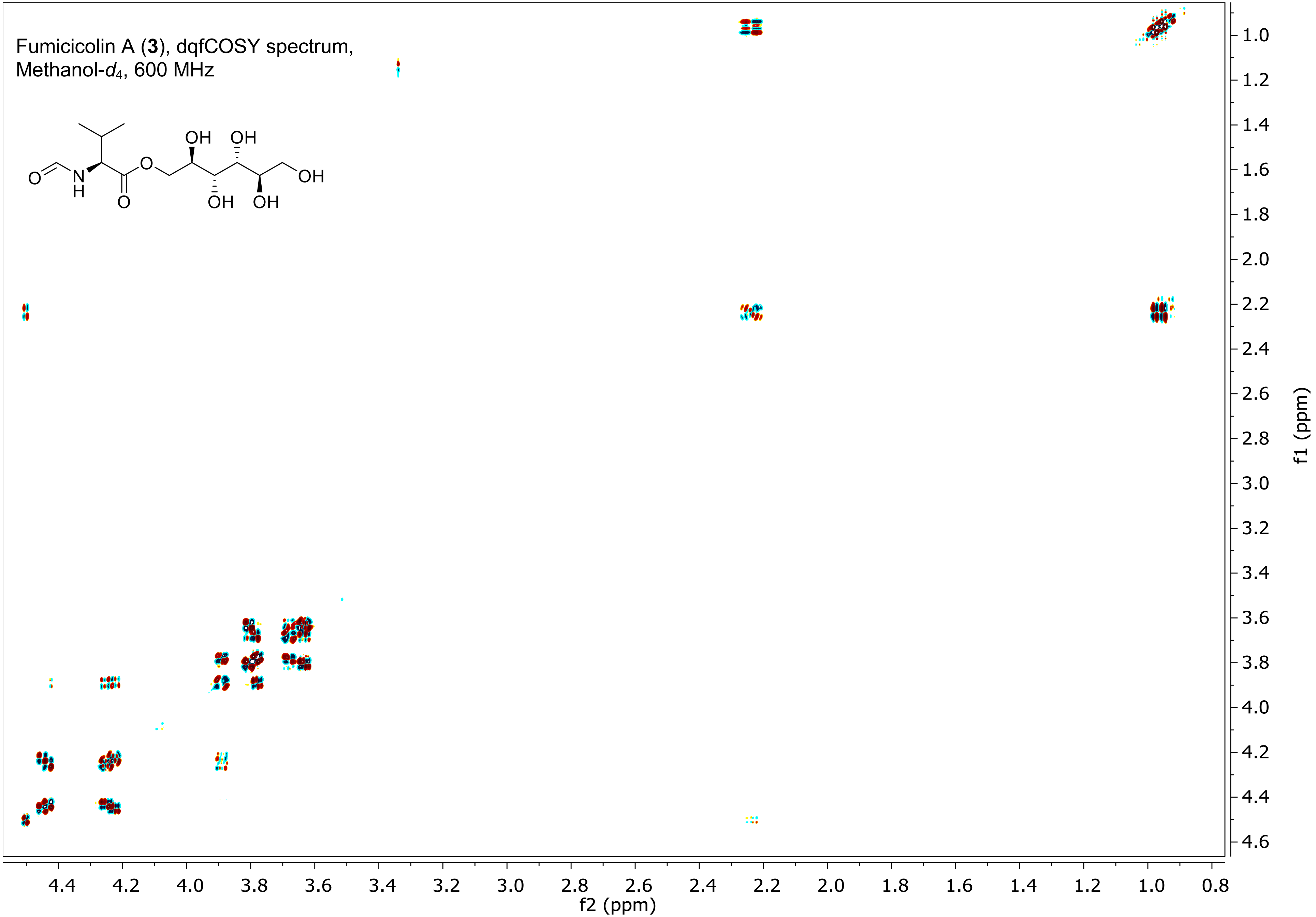

Fumicicolin A (**3**), dqfCOSY spectrum,
Methanol-d_4, 600 MHz
f2 (ppm)
f1 (ppm)

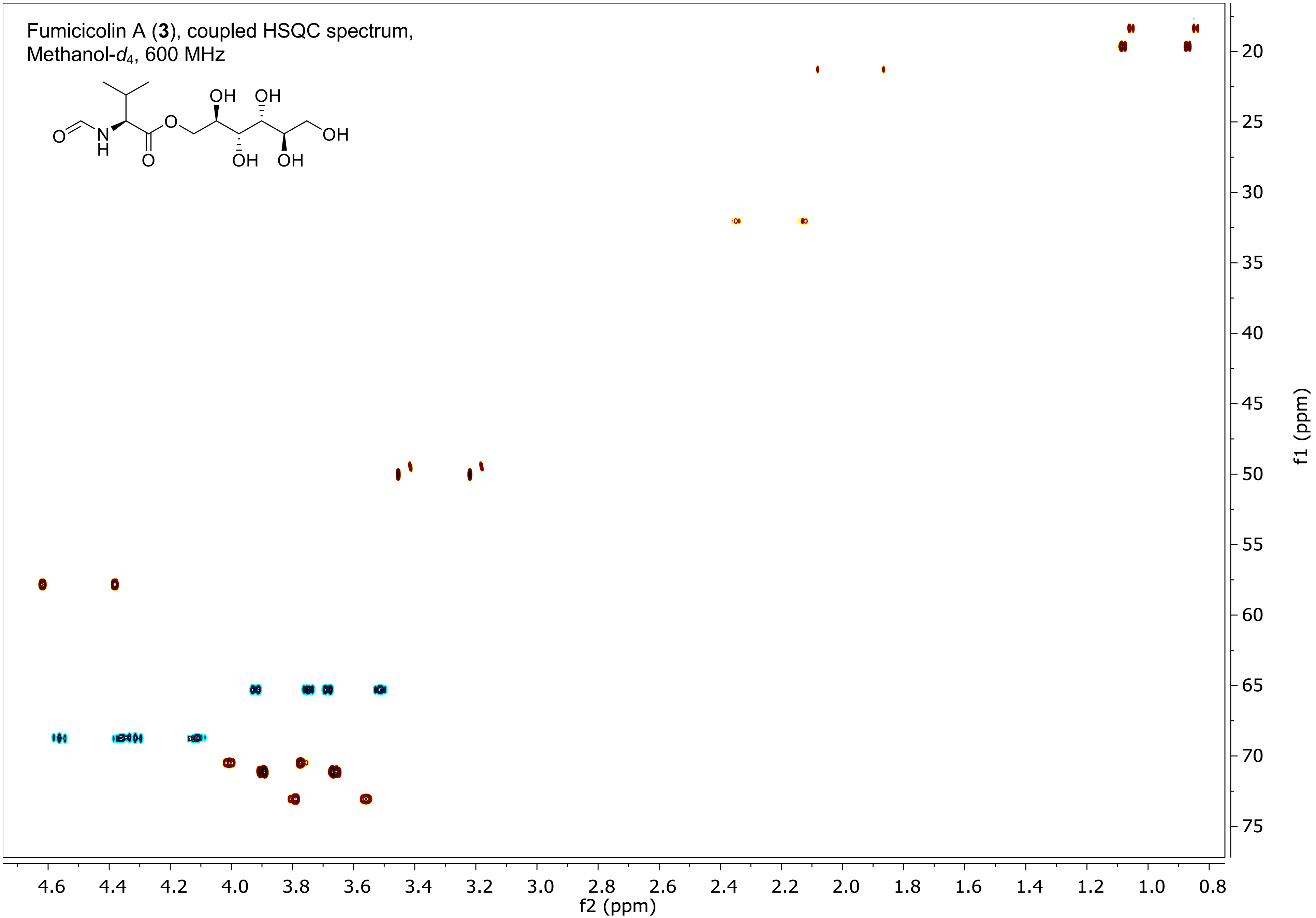
Fumicicolin A (3), coupled HSQC spectrum,
Methanol-d4, 600 MHz
OH OH
O N H O O OH
OH OH
f2 (ppm)
f1 (ppm)
4.6 4.4 4.2 4.0 3.8 3.6 3.4 3.2 3.0 2.8 2.6 2.4 2.2 2.0 1.8 1.6 1.4 1.2 1.0 0.8
20 25 30 35 40 45 50 55 60 65 70 75

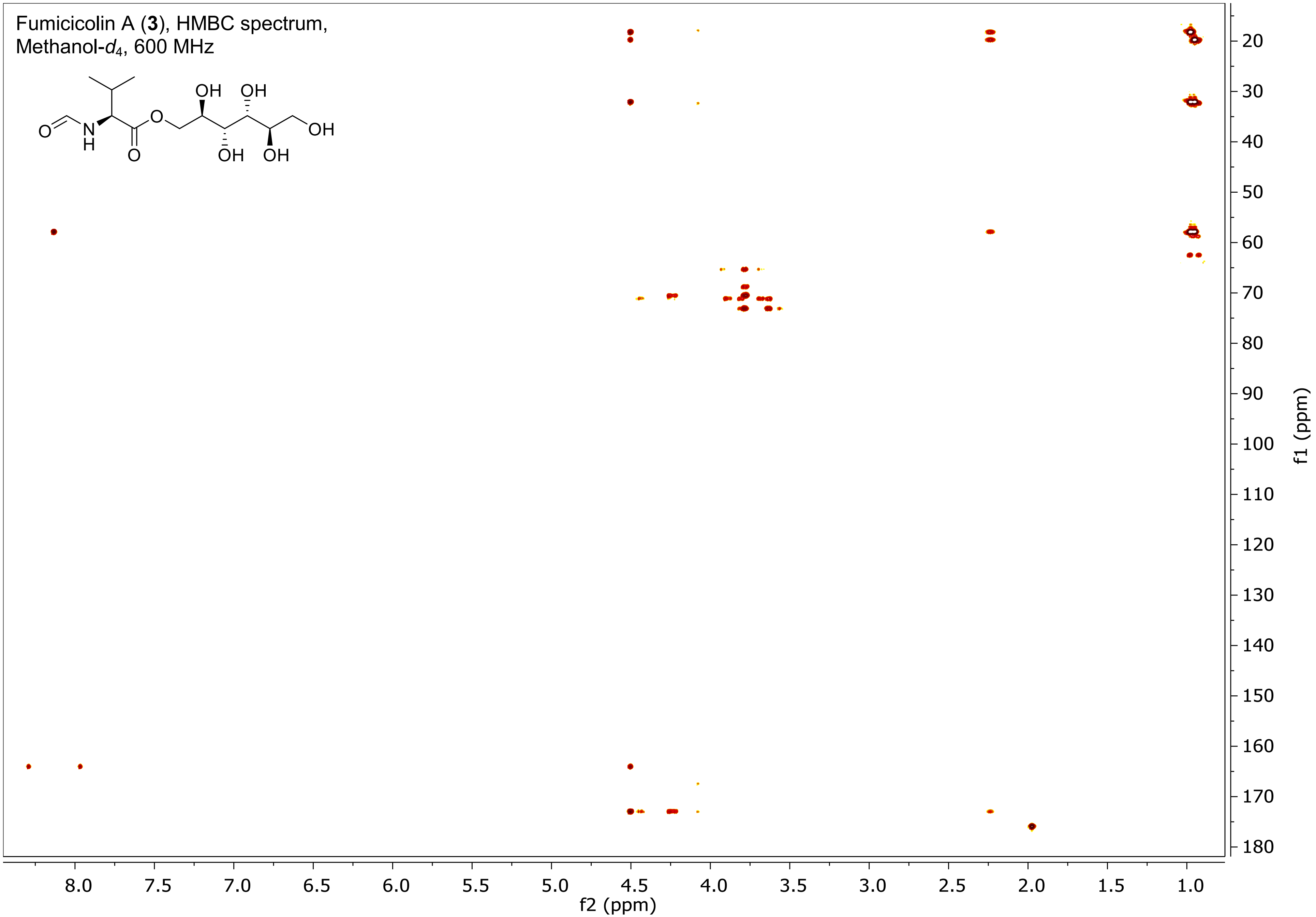

Fumicicolin A (3), HMBC spectrum,
Methanol-d_4, 600 MHz
OH
OH
OH
OH
OH
O
N
H
O
O
f2 (ppm)
f1 (ppm)

^{1}H (800 MHz) and ^{13}C (201 MHz) NMR spectroscopic data for *N*-formylvaline (4) in DMSO-d_6

Chemical shifts were referenced to $\delta(C\underline{H}D_2SOCD_3)$ = 2.50 and $\delta(^{13}\underline{C}HD_2SOCD_3)$ = 39.5. ^{13}C chemical shifts were determined via HMBC and HSQC spectra. One-bond (^{13}C,^{1}H)-*J*-coupling constants were determined from the acquired HSQC spectra. (^{1}H,^{1}H)-*J*-coupling constants were determined from the ^{1}H or dqfCOSY spectra. HMBC correlations are from the proton(s) stated to the indicated ^{13}C atom.

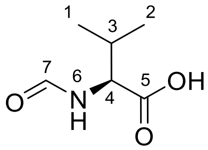

No.	δc	Proton	$^{1}J_{CH}$	δH (J_{HH}[Hz])	HMBC
1	17.9	1-H	124.5	0.79 ($J_{1,3}$ = 6.5 Hz)	2
2	19.7	2-H	124.6	0.77 ($J_{2,3}$ = 6.5 Hz)	1
3	30.8	3-H	128.7	2.04 ($J_{3,1}$ = 6.5 Hz, $J_{3,2}$ = 6.5 Hz, $J_{3,4}$ = 7.5 Hz)	1, 2
4	57.9	4-H	136.7	3.78 ($J_{4,3}$ = 7.5 Hz)	1, 2, 7
5	ND				
6		N-H		7.43 (br s)	
7	160.1	7-H	187.7	8.01	

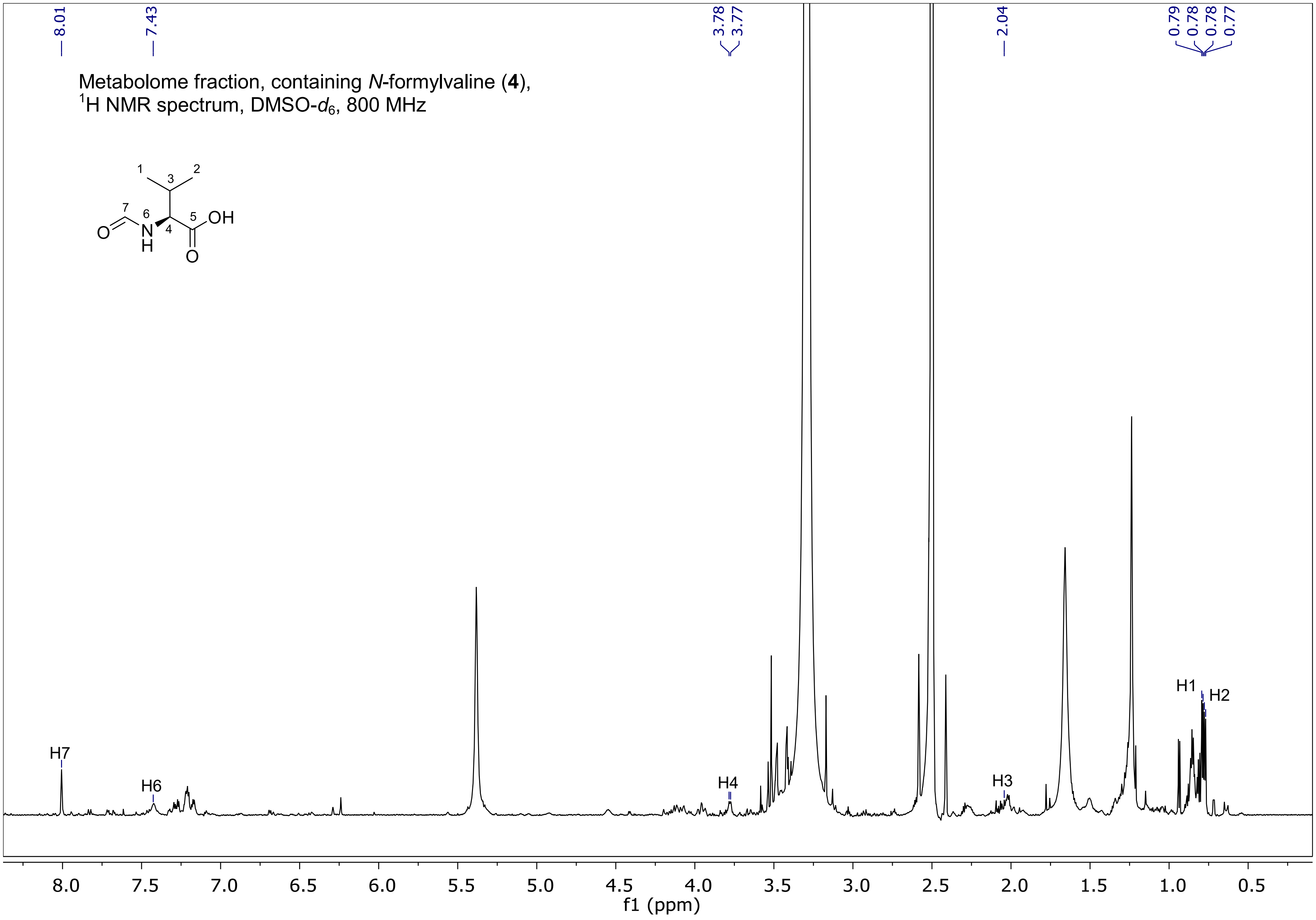
8.01
7.43
3.78
3.77
2.04
0.79
0.78
0.78
0.77
Metabolome fraction, containing *N*-formylvaline (**4**),
^{1}H NMR spectrum, DMSO-d_6, 800 MHz
1
2
3
7
6
5
4
OH
O
N
H
O
H7
H6
H4
H3
H1
H2
8.0
7.5
7.0
6.5
6.0
5.5
5.0
4.5
4.0
3.5
3.0
2.5
2.0
1.5
1.0
0.5
f1 (ppm)

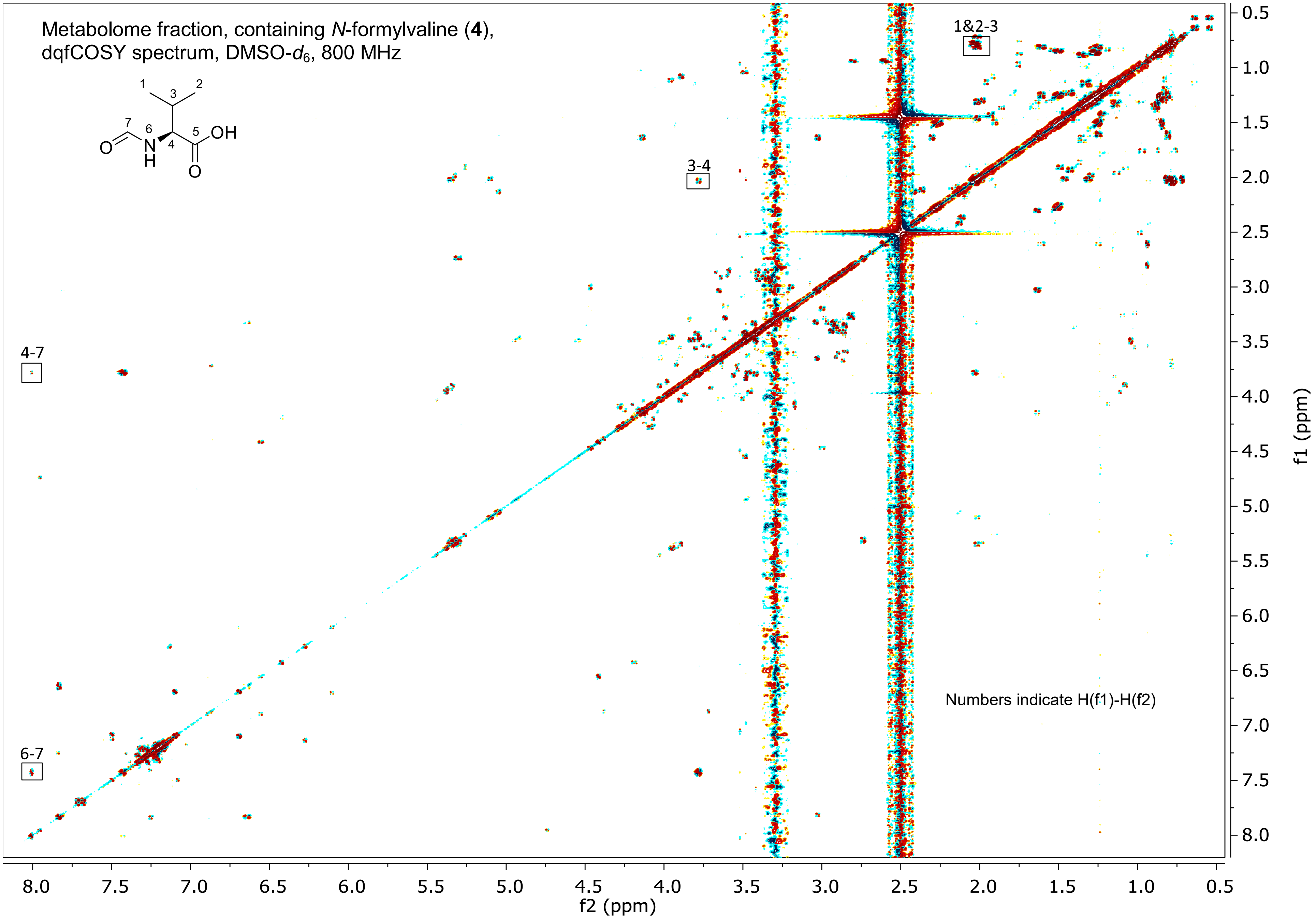
Metabolome fraction, containing N-formylvaline (4),
dqfCOSY spectrum, DMSO-d_6, 800 MHz
1&2-3
3-4
4-7
6-7
Numbers indicate H(f1)-H(f2)
f2 (ppm)
f1 (ppm)

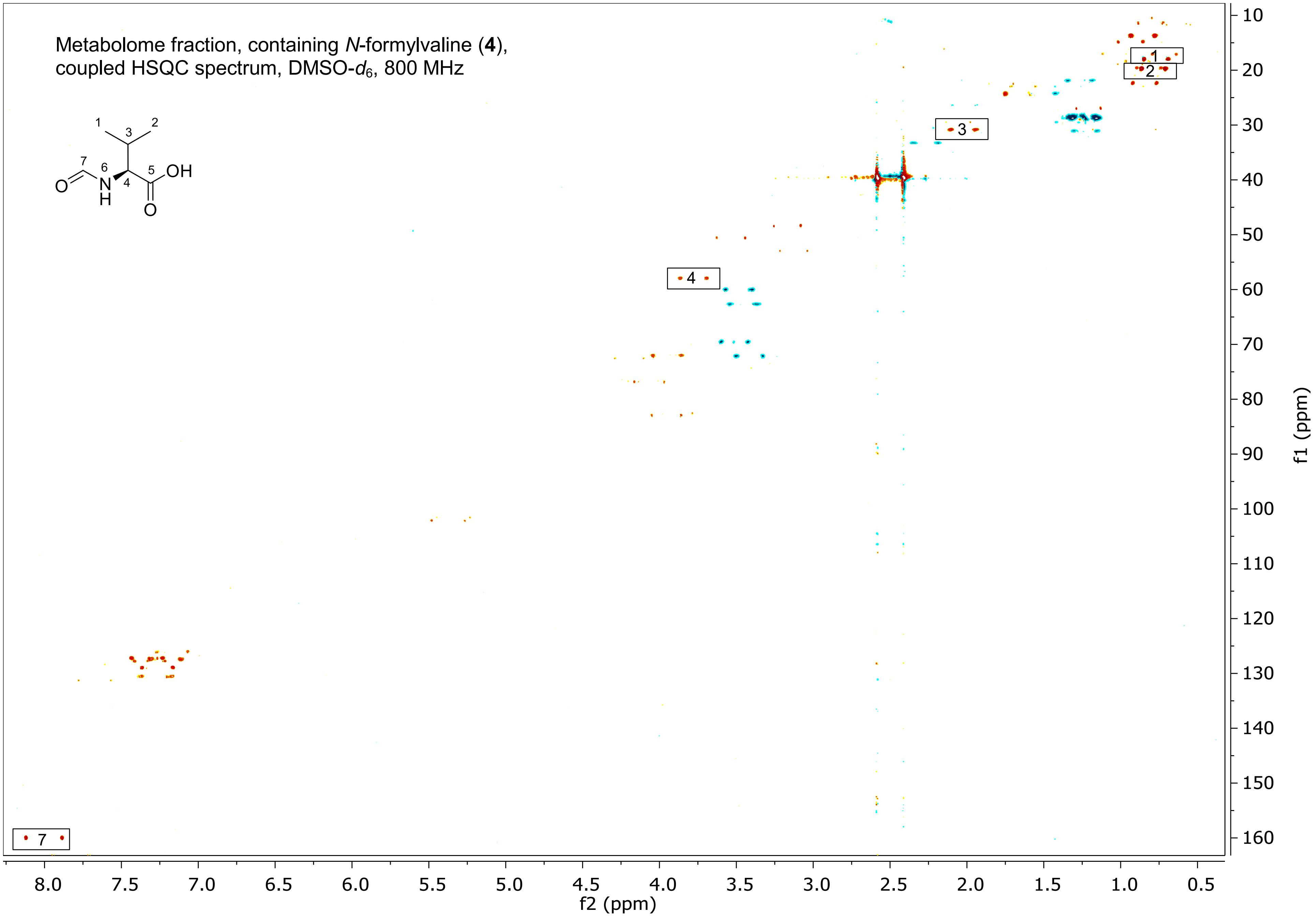

Metabolome fraction, containing *N*-formylvaline (**4**),
coupled HSQC spectrum, DMSO-d_6, 800 MHz
f2 (ppm)
f1 (ppm)

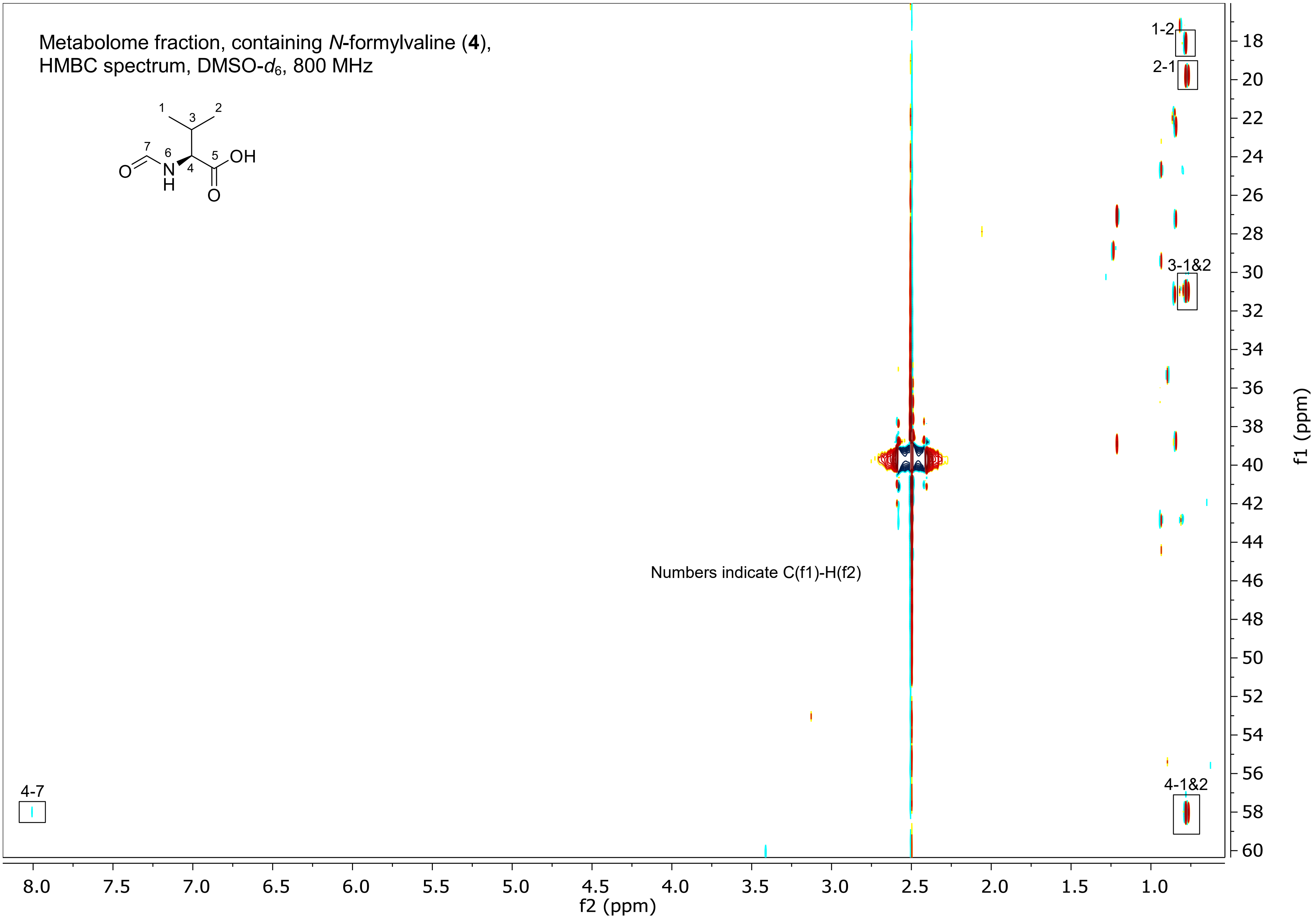

Metabolome fraction, containing N-formylvaline (4),
HMBC spectrum, DMSO-d6, 800 MHz
1
2
3
4
5
6
7
OH
O
N
H
O
1-2
2-1
3-1&2
4-1&2
4-7
Numbers indicate C(f1)-H(f2)
f1 (ppm)
f2 (ppm)

^{1}H (600 MHz) and ^{13}C (151 MHz) NMR spectroscopic data for (*S*)-2-isocyanoisovaleric acid (5) in D_2O

Chemical shifts were referenced to δ(<u>H</u>DO) = 4.80 and δ(^{13}<u>C</u>H_3OD) = 49.0. (^{1}H,^{1}H)-*J*-coupling constants were determined from the acquired ^{1}H or dqfCOSY spectra. HMBC correlations are from the proton(s) stated to the indicated ^{13}C atom.

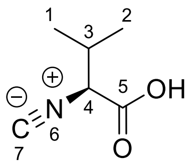

No.	δc	Proton	δH (J_{HH}[Hz])	HMBC
1	16.0	1-H	0.95 ($J_{1,3}$ = 6.7 Hz)	2, 3, 4
2	19.1	2-H	1.07 ($J_{2,3}$ = 6.7 Hz)	1, 3, 4
3	30.6	3-H	2.28 ($J_{3,1}$ = 6.7 Hz, $J_{3,2}$ = 6.5 Hz, $J_{3,4}$ = 7.5 Hz)	1, 2, 4
4	66.0	4-H	4.21 ($J_{4,3}$ = 7.5 Hz)	1, 2, 3
5	173.5			4
7	153.1			4

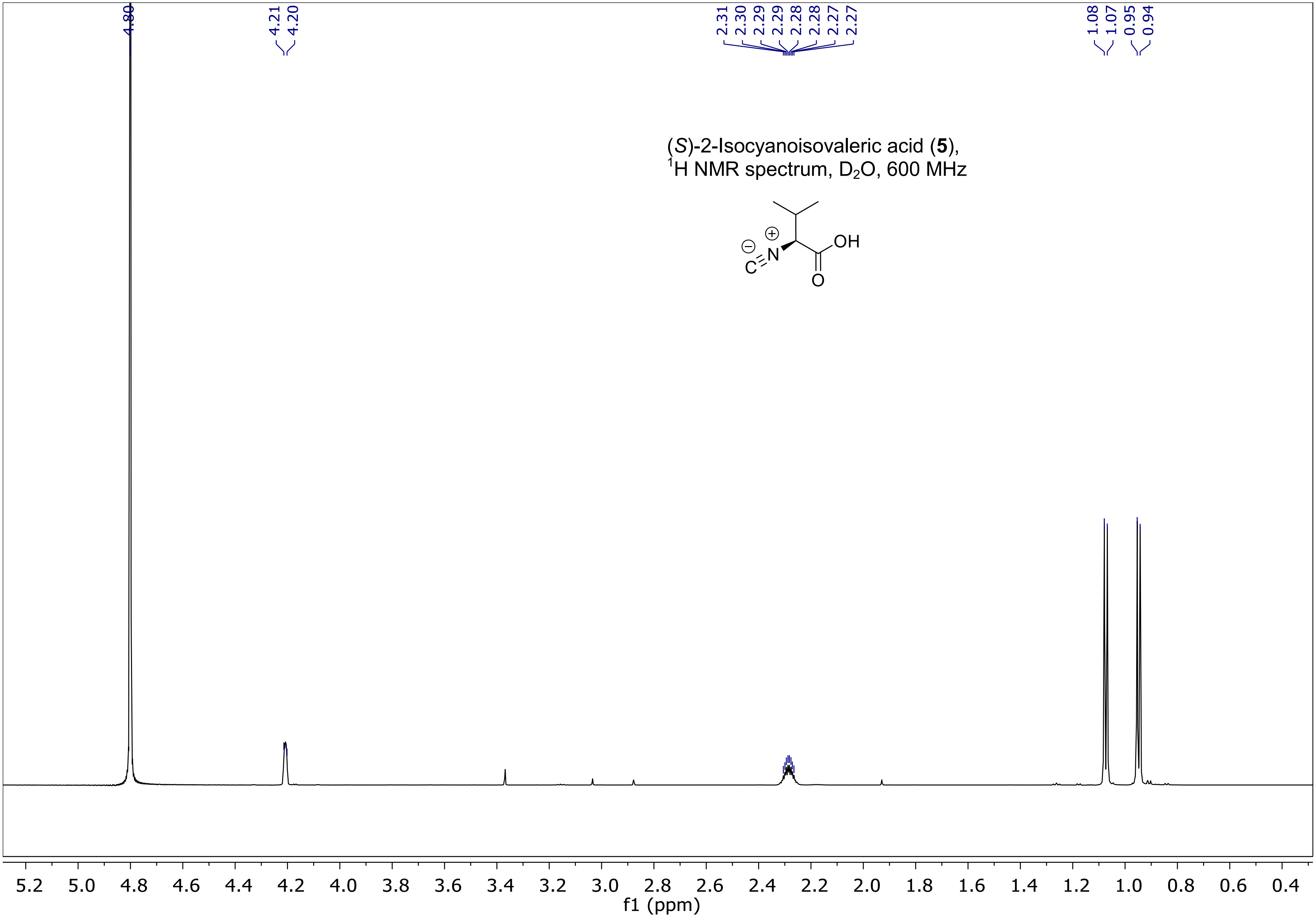
4.80
4.21
4.20
2.31
2.30
2.29
2.29
2.28
2.28
2.27
2.27
1.08
1.07
0.95
0.94
(S)-2-Isocyanoisovaleric acid (5),
¹H NMR spectrum, D₂O, 600 MHz
OH
N
C
O
5.2 5.0 4.8 4.6 4.4 4.2 4.0 3.8 3.6 3.4 3.2 3.0 2.8 2.6 2.4 2.2 2.0 1.8 1.6 1.4 1.2 1.0 0.8 0.6 0.4
f1 (ppm)

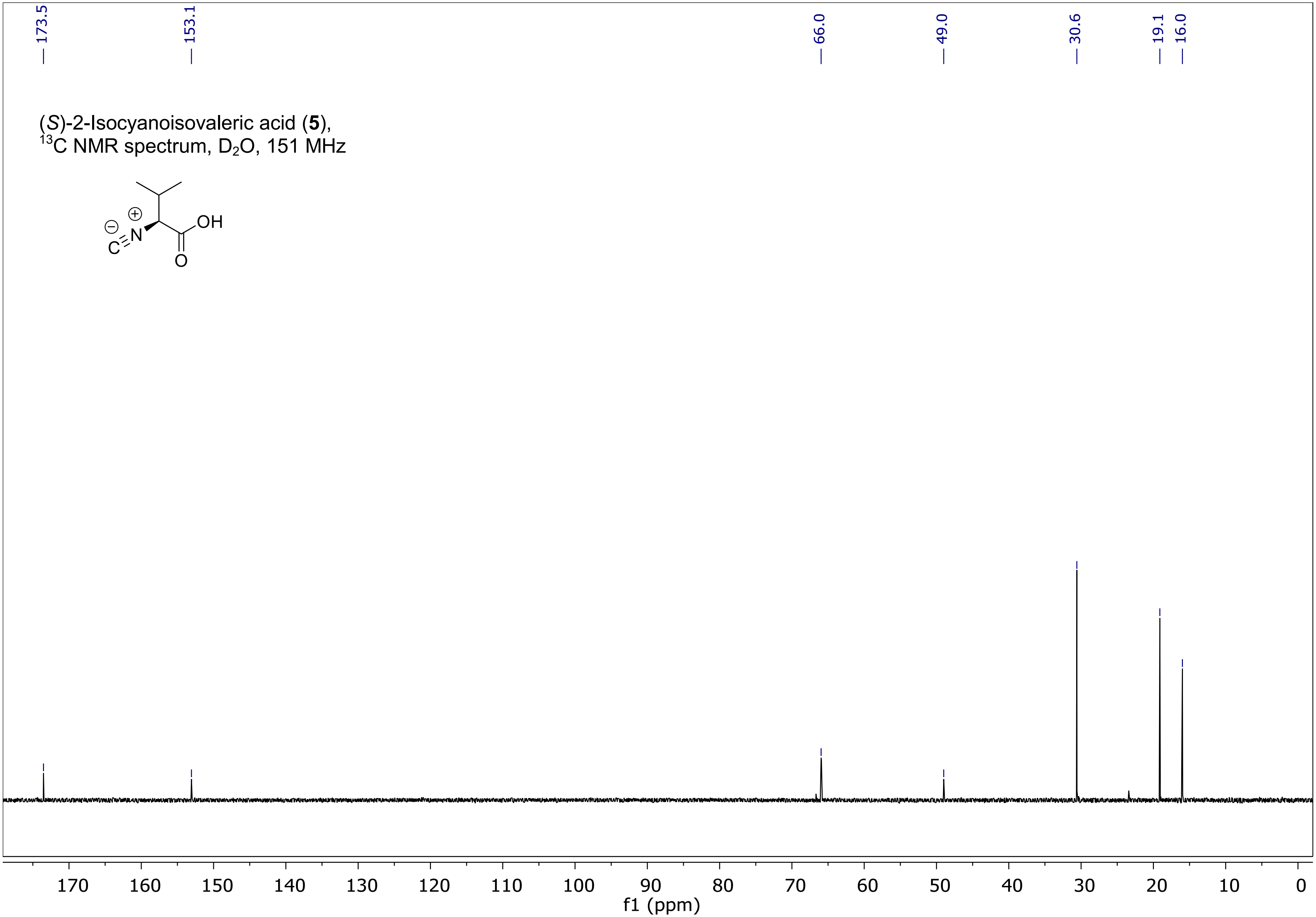
173.5
153.1
66.0
49.0
30.6
19.1
16.0
(S)-2-Isocyanoisovaleric acid (5),
13C NMR spectrum, D2O, 151 MHz
OH
N
C
O
170 160 150 140 130 120 110 100 90 80 70 60 50 40 30 20 10 0
f1 (ppm)

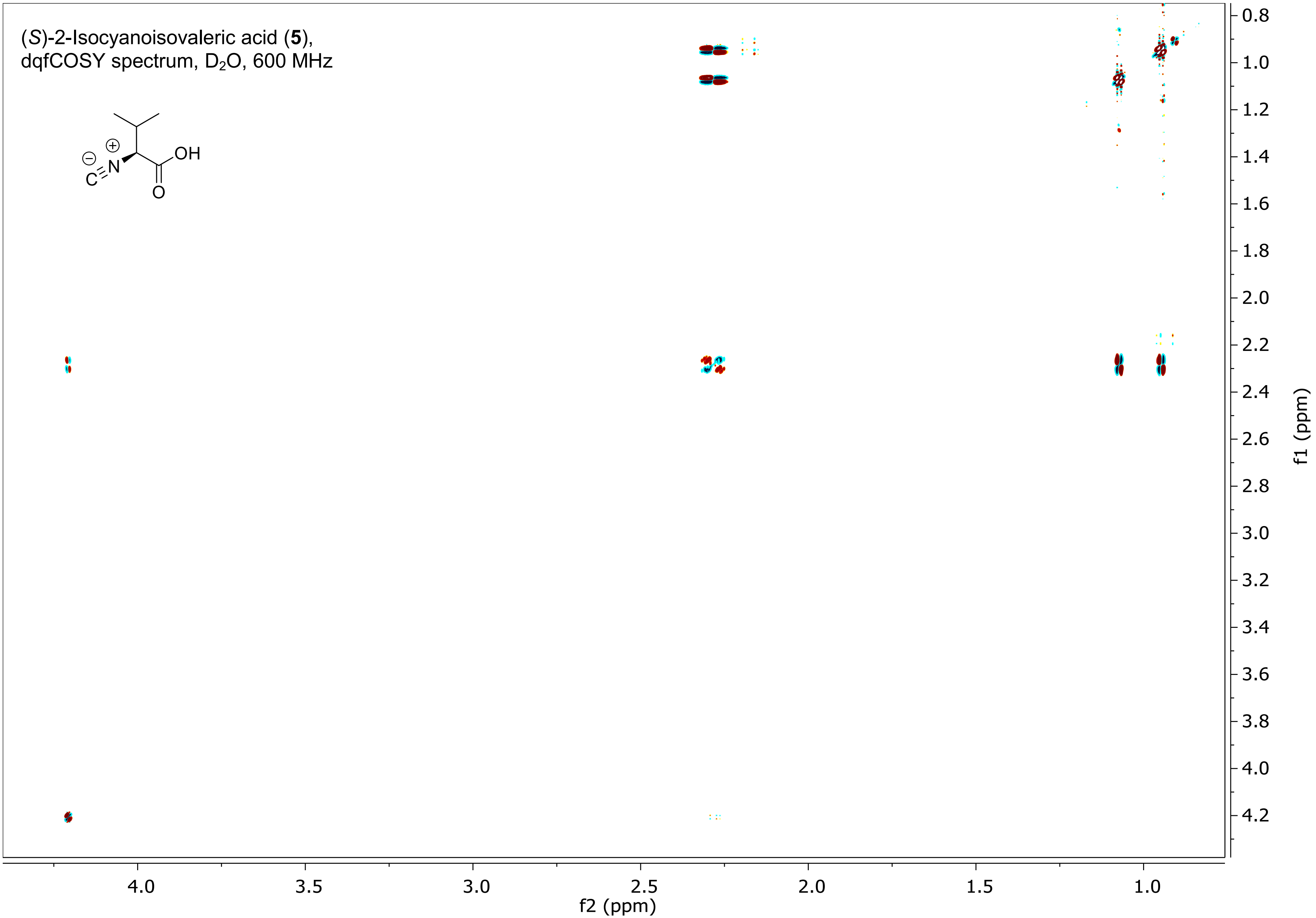
(S)-2-Isocyanoisovaleric acid (5),
dqfCOSY spectrum, D2O, 600 MHz
OH
O
N
C
f1 (ppm)
f2 (ppm)
4.0
3.5
3.0
2.5
2.0
1.5
1.0
0.8
1.2
1.4
1.6
1.8
2.2
2.4
2.6
2.8
3.2
3.4
3.6
3.8
4.2

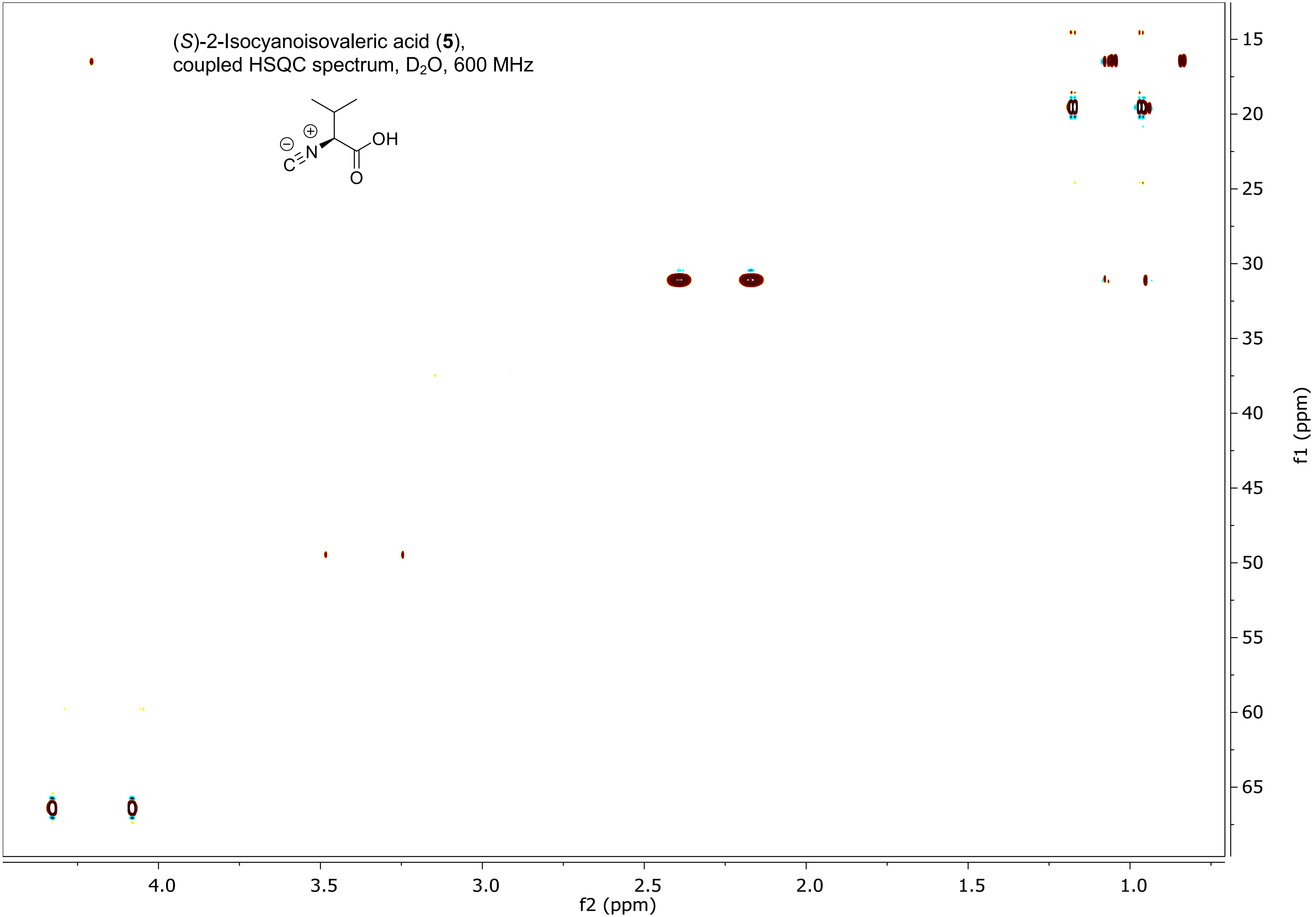
(S)-2-Isocyanoisovaleric acid (5),
coupled HSQC spectrum, D2O, 600 MHz
OH
N
C
O
f2 (ppm)
f1 (ppm)
4.0
3.5
3.0
2.5
2.0
1.5
1.0
15
20
25
30
35
40
45
50
55
60
65

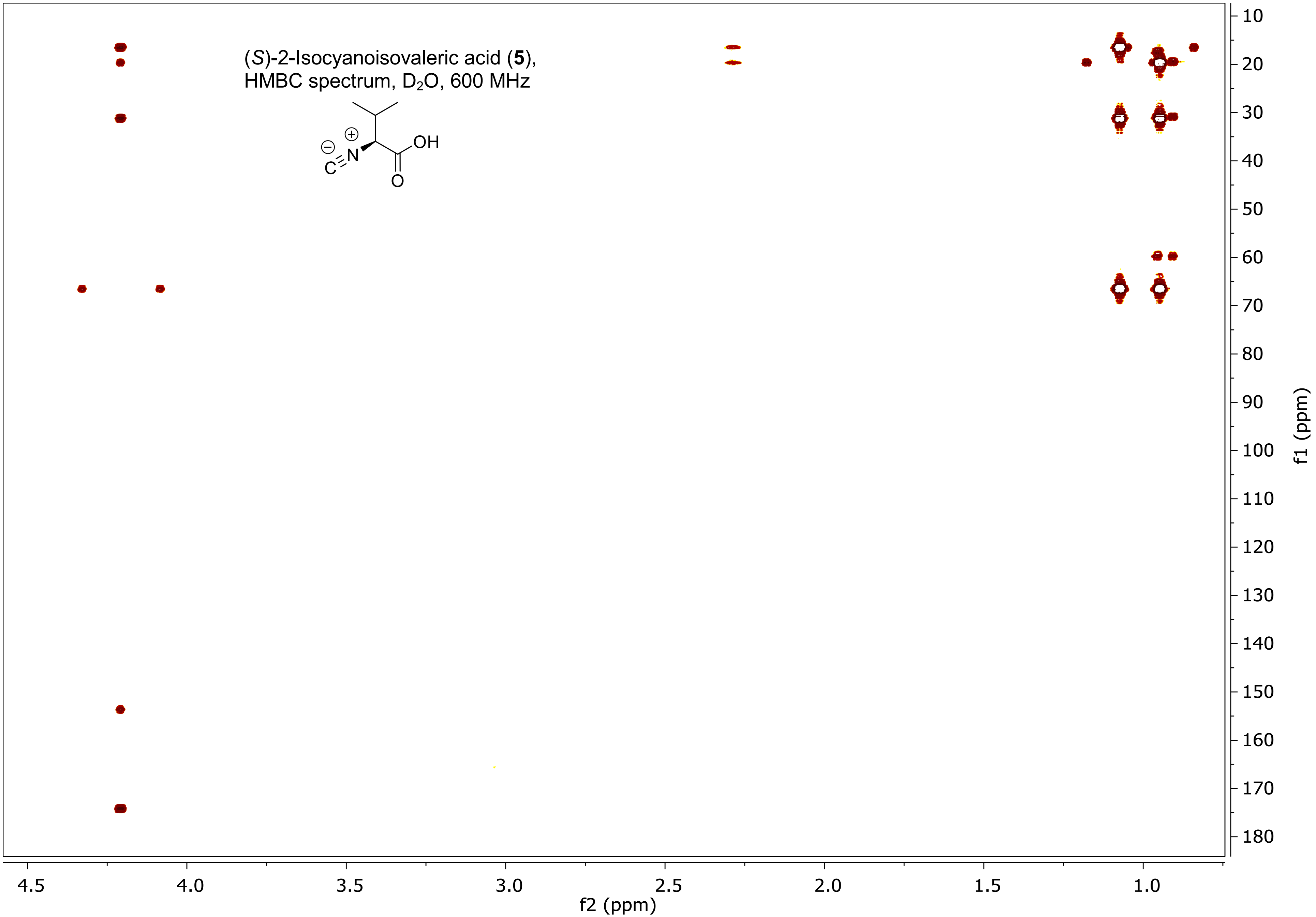
(S)-2-Isocyanoisovaleric acid (5),
HMBC spectrum, D2O, 600 MHz
OH
O
N
C
f2 (ppm)
f1 (ppm)

^{1}H (600 MHz) and ^{13}C (151 MHz) NMR spectroscopic data for heterocicolin C (12) in methanol-d_4

Chemical shifts were referenced to δ(C<u>H</u>D_2OD) = 3.31 and δ(^{13}<u>C</u>HD_2OD) = 49.0. (^{1}H,^{1}H)-*J*-coupling constants were determined from the ^{1}H or dqfCOSY spectra. HMBC correlations are from the proton(s) stated to the indicated ^{13}C atom.

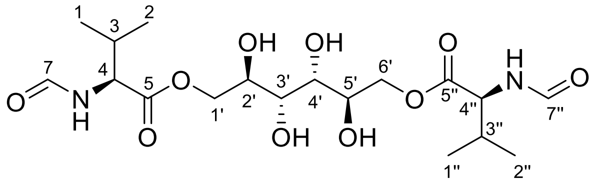

No.	δc	Proton	δH (J_{HH}[Hz])	HMBC
1/1''	18.0	1/1''-H	0.96 ($J_{1,3}$ = 6.7 Hz)	2, 3, 4
2/2''	19.5	3/3''-H	0.99 ($J_{2,3}$ = 6.7 Hz)	1, 3, 4
3/3''	31.9	3/3''-H	2.25 ($J_{3,1}$ = 5.5 Hz, $J_{3,2}$ = 6.7 Hz, $J_{3,4}$ = 6.7 Hz)	1, 2, 4
4/4''	57.7	4/4''-H	4.51 ($J_{4,3}$ = 5.5 Hz)	1, 2, 3, 7
5/5''	172.7			1'/6'b, 3, 4
7/7''	163.8	7/7''-H	8.14	4
1'/6'	68.6	1'/6'-Ha	4.47 ($J_{1'a,1'b}$ = 11.5 Hz)	3', 4'
		1'/6'-Hb	4.45 ($J_{1'a,1'b}$ = 11.5 Hz, $J_{1'a,2'}$ = 6.7 Hz)	1, 2, 4
2'/5'	70.3	2'/5'-H	3.89 ($J_{3',2'}$ = 8.5 Hz, $J_{2',1'b}$ = 6.7 Hz)	1'/6'b, 3', 4'
3'/4'	70.5	3'/4'-H	3.81 ($J_{3',2'}$ = 8.5 Hz)	2', 5'

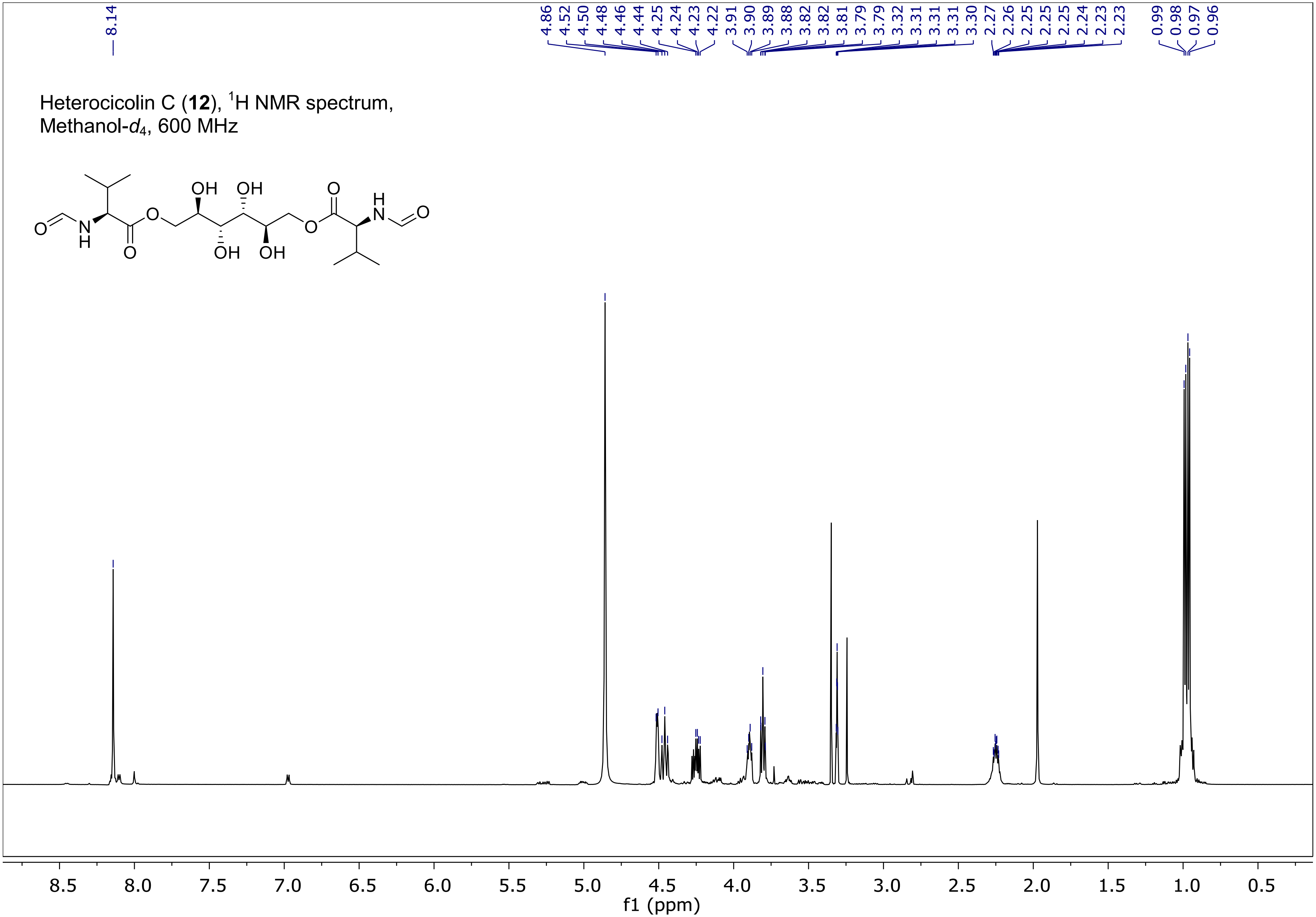
8.14
4.86 4.52 4.50 4.48 4.46 4.44 4.25 4.24 4.23 4.22 3.91 3.90 3.89 3.88 3.82 3.82 3.81 3.79 3.79 3.32 3.31 3.31 3.31 3.30 2.27 2.26 2.25 2.25 2.25 2.24 2.23 2.23 0.99 0.98 0.97 0.96
Heterocicolin C (12), 1H NMR spectrum,
Methanol-d4, 600 MHz
OH
OH
O
H
N
O
O
N
H
O
O
OH
OH
O
8.5 8.0 7.5 7.0 6.5 6.0 5.5 5.0 4.5 4.0 3.5 3.0 2.5 2.0 1.5 1.0 0.5
f1 (ppm)

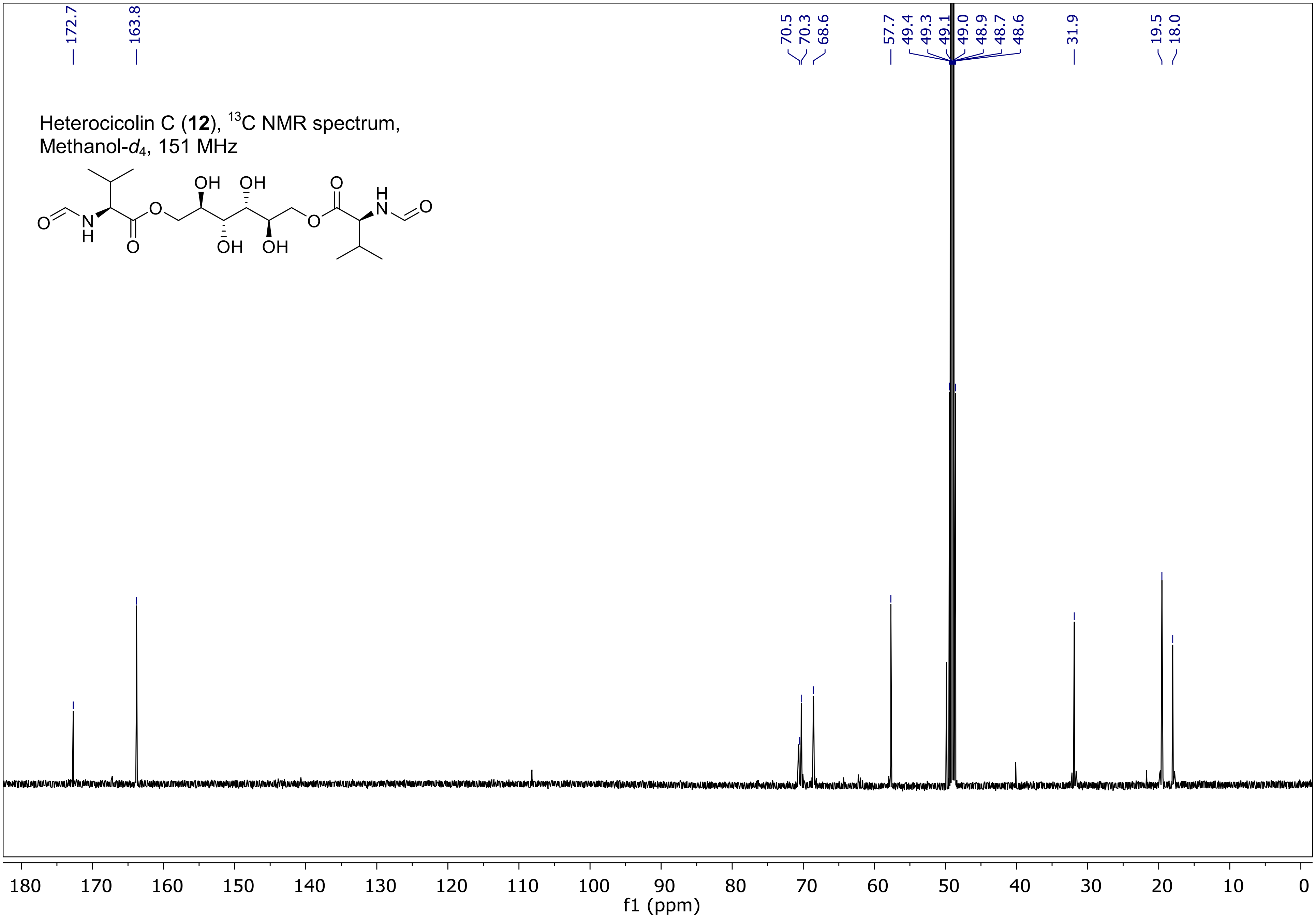
Heterocicolin C (12), 13C NMR spectrum,
Methanol-d4, 151 MHz
172.7
163.8
70.5
70.3
68.6
57.7
49.4
49.3
49.1
49.0
48.9
48.7
48.6
31.9
19.5
18.0
180 170 160 150 140 130 120 110 100 90 80 70 60 50 40 30 20 10 0
f1 (ppm)

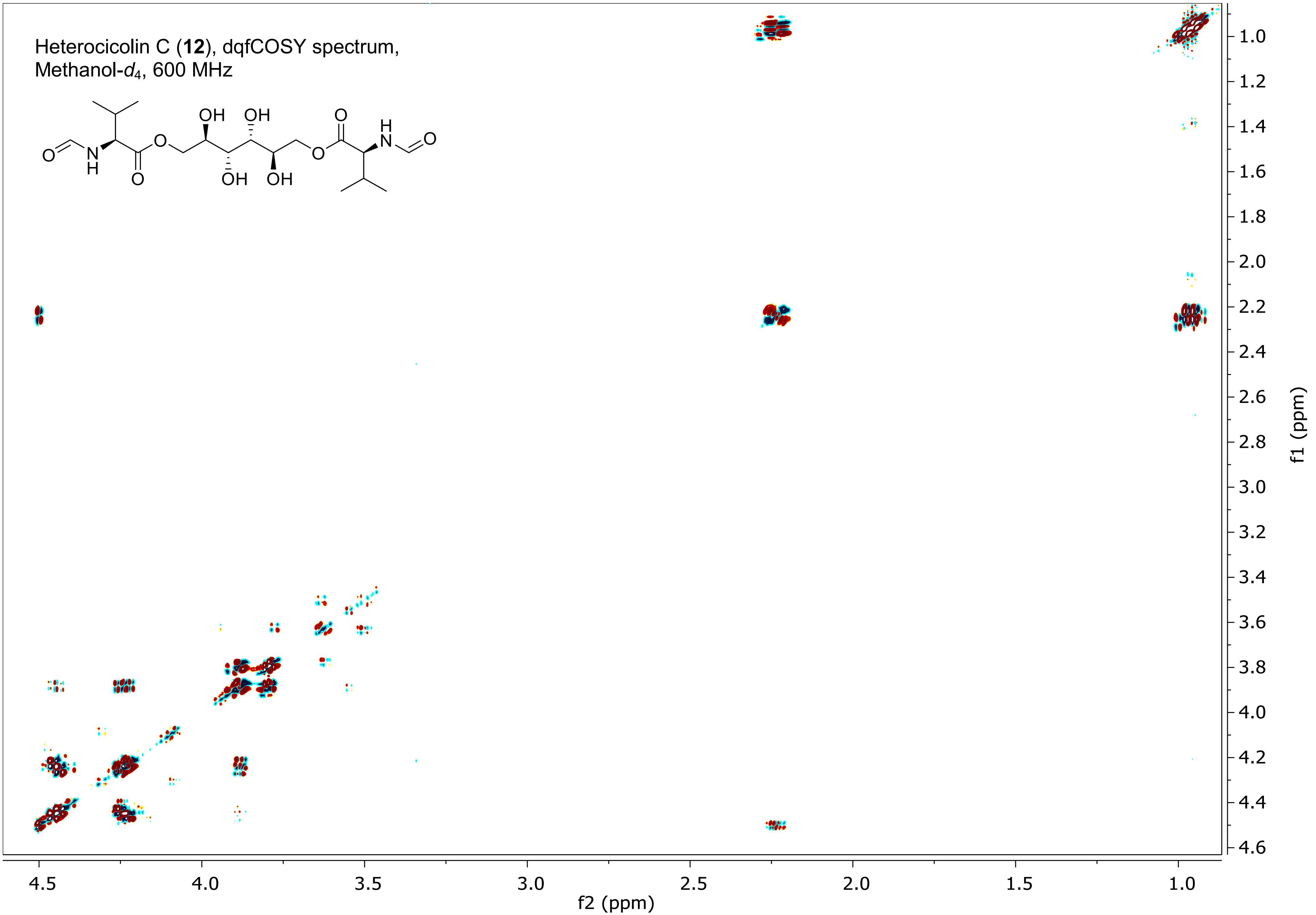
Heterocicolin C (12), dqfCOSY spectrum,
Methanol-d_4, 600 MHz
f2 (ppm)
f1 (ppm)

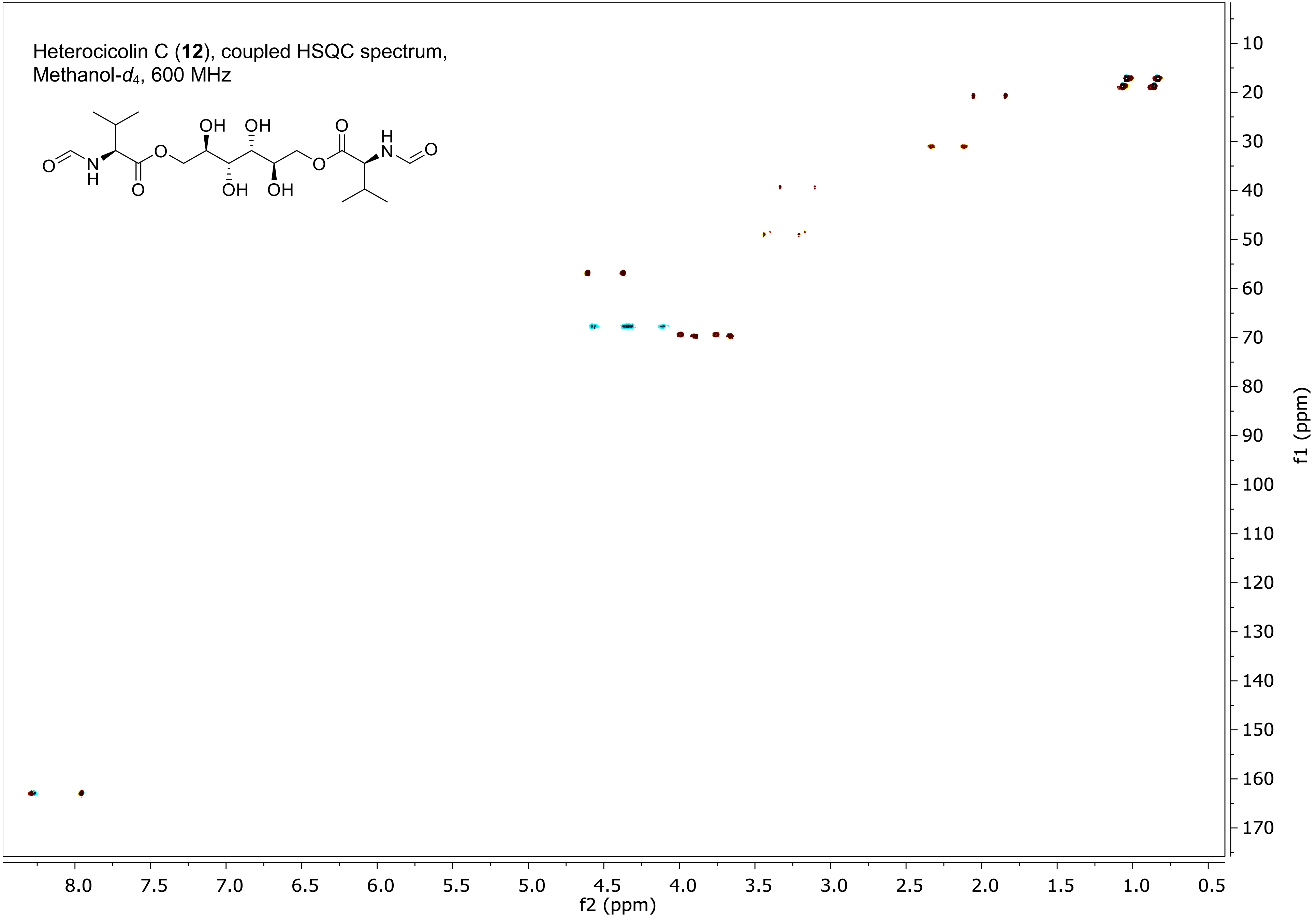

Heterocicolin C (12), coupled HSQC spectrum,
Methanol-d4, 600 MHz
f2 (ppm)
f1 (ppm)

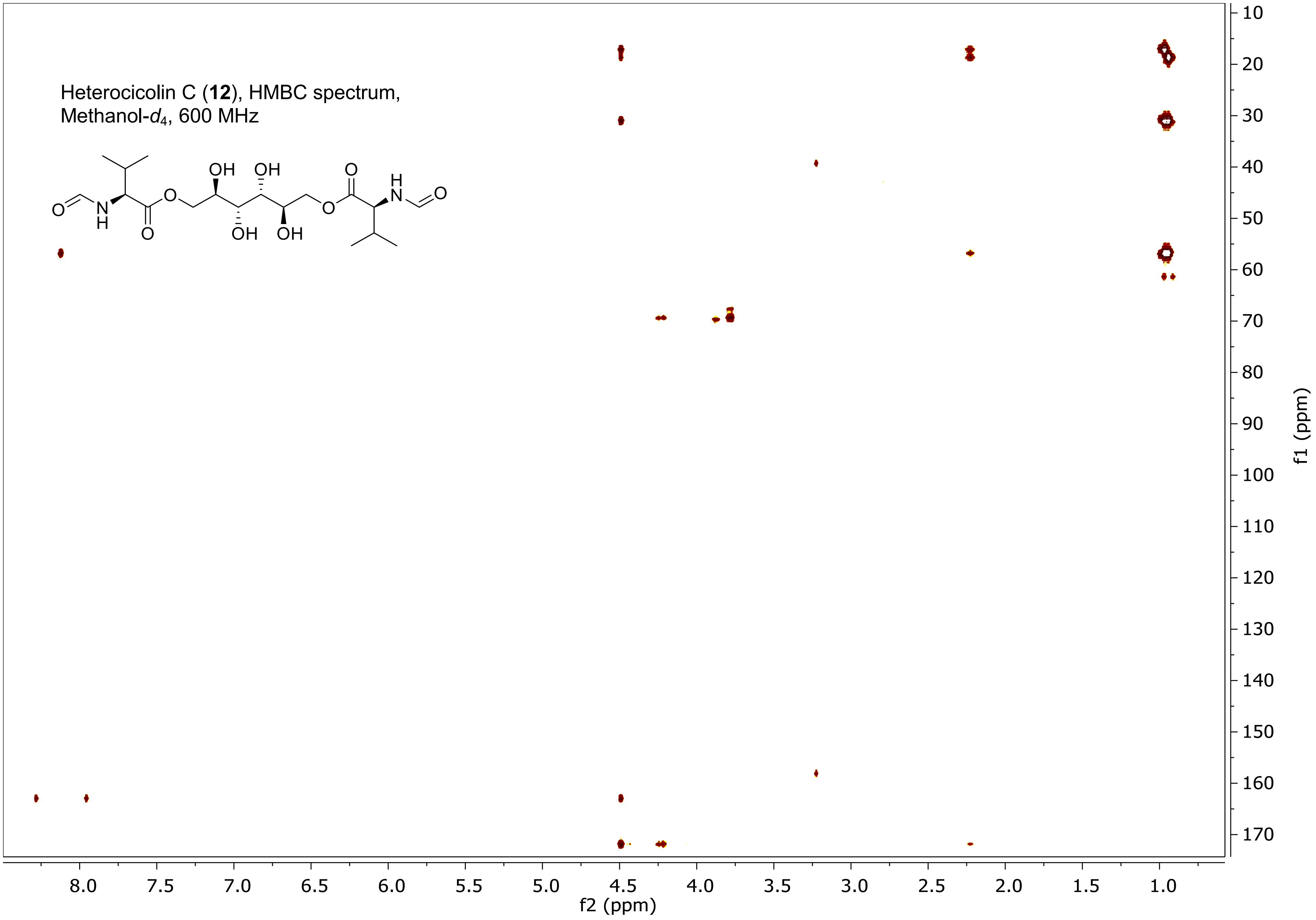
Heterocicolin C (12), HMBC spectrum,
Methanol-d_4, 600 MHz
f2 (ppm)
f1 (ppm)

^{1}H (600 MHz) and ^{13}C (151 MHz) NMR spectroscopic data for heterocicolin E (14) in methanol-d_4

Chemical shifts were referenced to δ(C<u>H</u>D$_2$OD) = 3.31 and δ(^{13}<u>C</u>HD$_2$OD) = 49.0. (^{1}H,^{1}H)-J-coupling constants were determined from the ^{1}H or dqfCOSY spectra. HMBC correlations are from the proton(s) stated to the indicated ^{13}C atom.

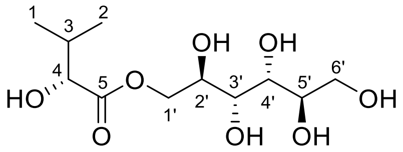

No.	δc	Proton	δH (J_{HH}[Hz])	HMBC
1	17.1	1-H	0.93 ($J_{1,3}$ = 7.0 Hz)	2, 3, 4
2	19.2	2-H	1.00 ($J_{2,3}$ = 7.0 Hz)	1, 3, 4
3	33.4	3-H	2.09 ($J_{3,1}$ = 7.0 Hz, $J_{3,2}$ = 7.0 Hz, $J_{3,4}$ = 4.5 Hz)	1, 2, 4
4	76.8	4-H	4.01 ($J_{4,3}$ = 4.5 Hz)	1, 2, 3
5	175.6			4, 1’
1’	68.2	1’-Ha	4.45 ($J_{1'a,1'b}$ = 11.5 Hz, $J_{1'a,2'}$ = 3.0 Hz)	3’
		1’-Hb	4.24 ($J_{1'b,1'a}$ = 11.5 Hz, $J_{1'b,2'}$ = 6.9 Hz)	
2’	70.4	2’-H	3.89 ($J_{2',1'a}$ = 3.0 Hz, $J_{2',1'b}$ = 6.9 Hz, $J_{2',3'}$ = 9.0 Hz)	1’
3’	71.0	3’-H	3.78 (m)	1’b, 2’, 4’, 5’
4’	71.0	1’-H	3.79 (m)	2’, 3’, 5’, 6’
5’	73.0	2’-H	3.69 ($J_{5',6'a}$ = 3.7 Hz, $J_{5',6'b}$ = 6.4 Hz, $J_{5',4'}$ = 6.4 Hz)	4’, 6b’
6’	65.2	6’-Ha	3.81 ($J_{6'a,6'b}$ = 11.5 Hz, $J_{6'a,5'}$ = 3.7 Hz)	4’, 5’
		6’-Hb	3.64 ($J_{6'b,6'a}$ = 11.5 Hz, $J_{6'b,5'}$ = 6.4 Hz)	

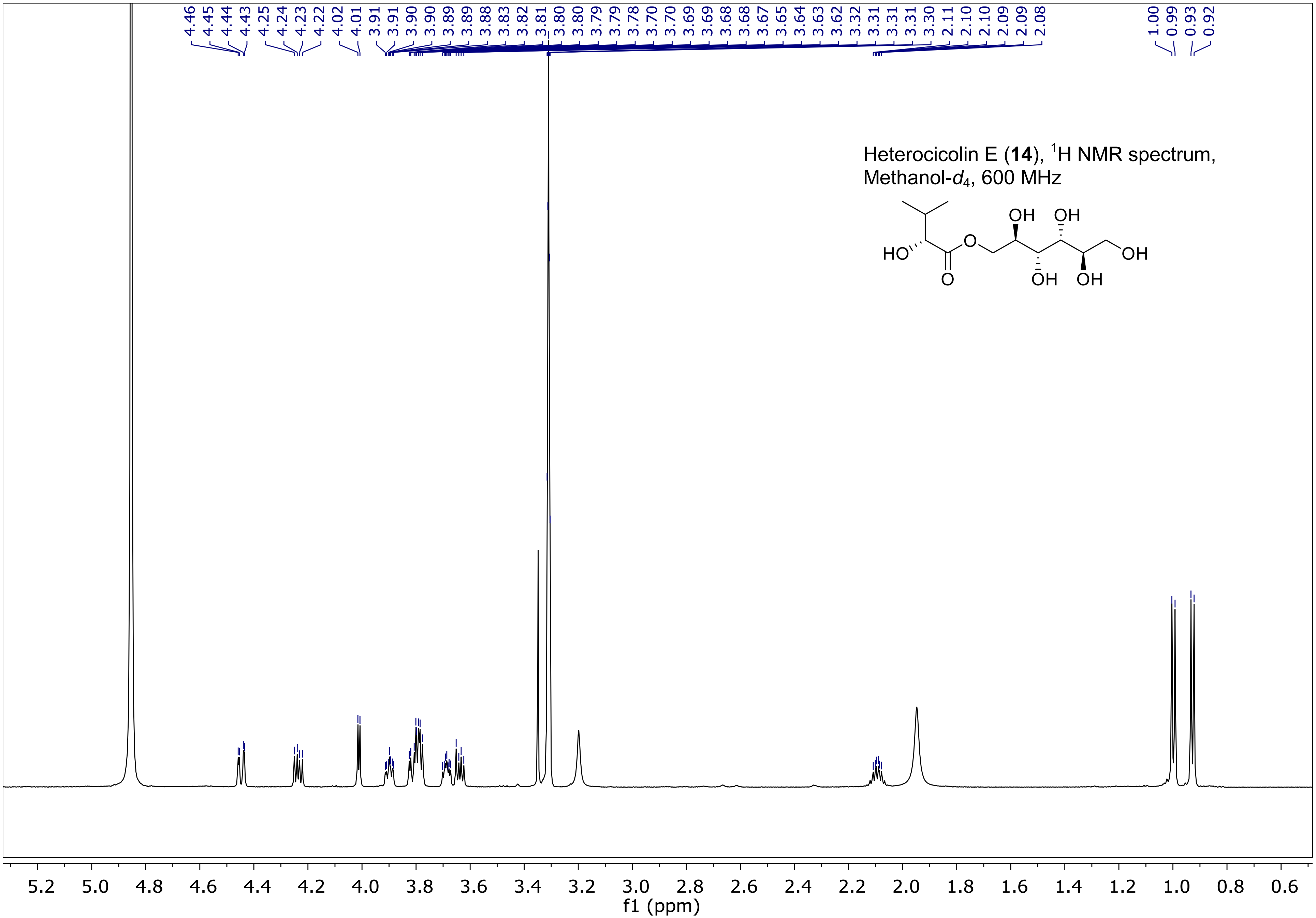
4.46 4.45 4.44 4.43 4.25 4.24 4.23 4.22 4.02 4.01 3.91 3.91 3.90 3.90 3.89 3.89 3.88 3.83 3.82 3.81 3.80 3.80 3.79 3.79 3.78 3.70 3.70 3.69 3.69 3.68 3.68 3.67 3.65 3.64 3.63 3.62 3.32 3.31 3.31 3.31 3.30 2.11 2.10 2.10 2.09 2.09 2.08
1.00 0.99 0.93 0.92
Heterocicolin E (**14**), ^{1}H NMR spectrum,
Methanol-d_4, 600 MHz
HO
O
O
OH
OH
OH
OH
OH
5.2 5.0 4.8 4.6 4.4 4.2 4.0 3.8 3.6 3.4 3.2 3.0 2.8 2.6 2.4 2.2 2.0 1.8 1.6 1.4 1.2 1.0 0.8 0.6
f1 (ppm)

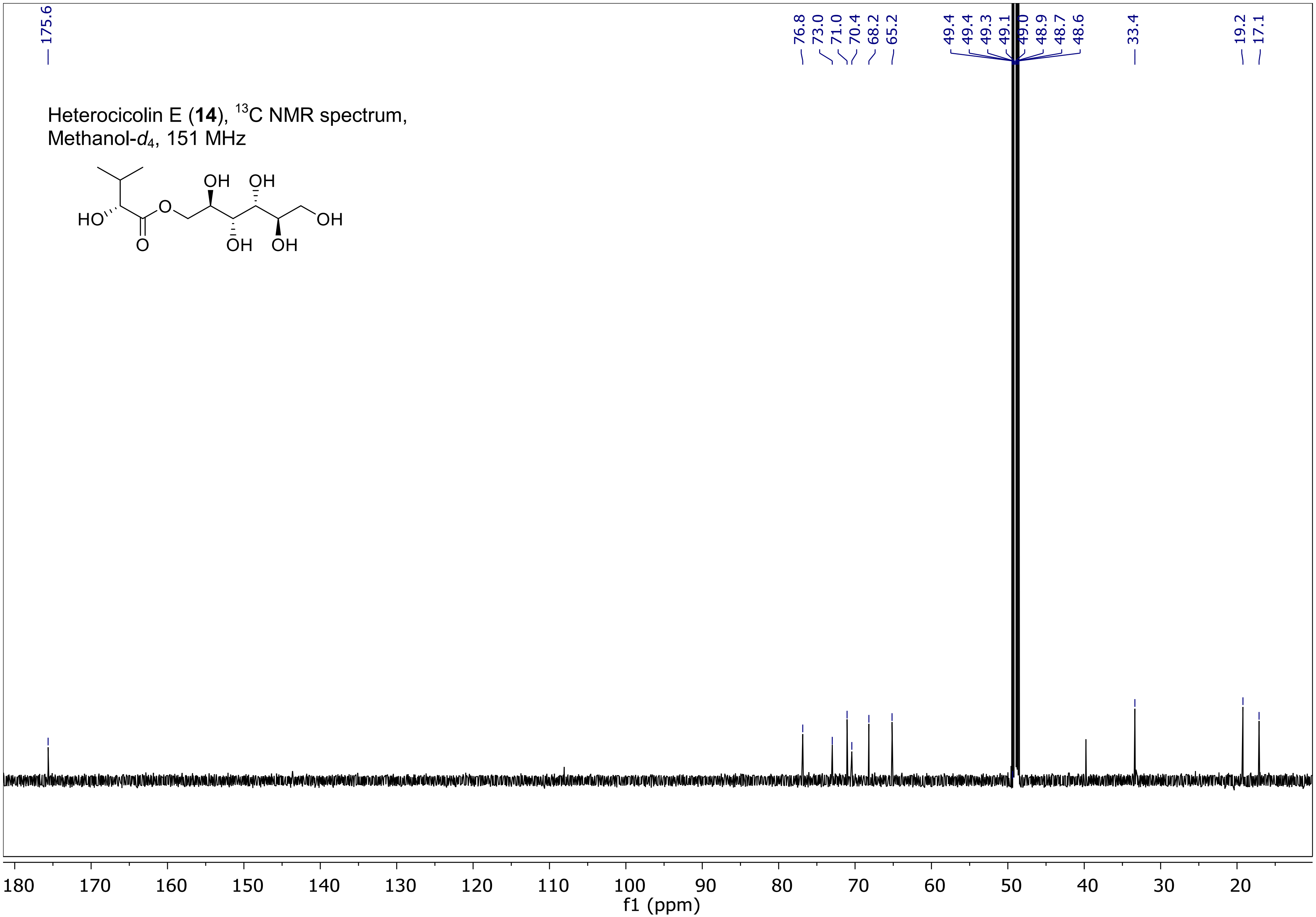

175.6
76.8
73.0
71.0
70.4
68.2
65.2
49.4
49.4
49.3
49.1
49.0
48.9
48.7
48.6
33.4
19.2
17.1
Heterocicolin E (14), 13C NMR spectrum,
Methanol-d4, 151 MHz
OH
OH
HO
O
OH
O
OH
OH
180
170
160
150
140
130
120
110
100
90
80
70
60
50
40
30
20
f1 (ppm)

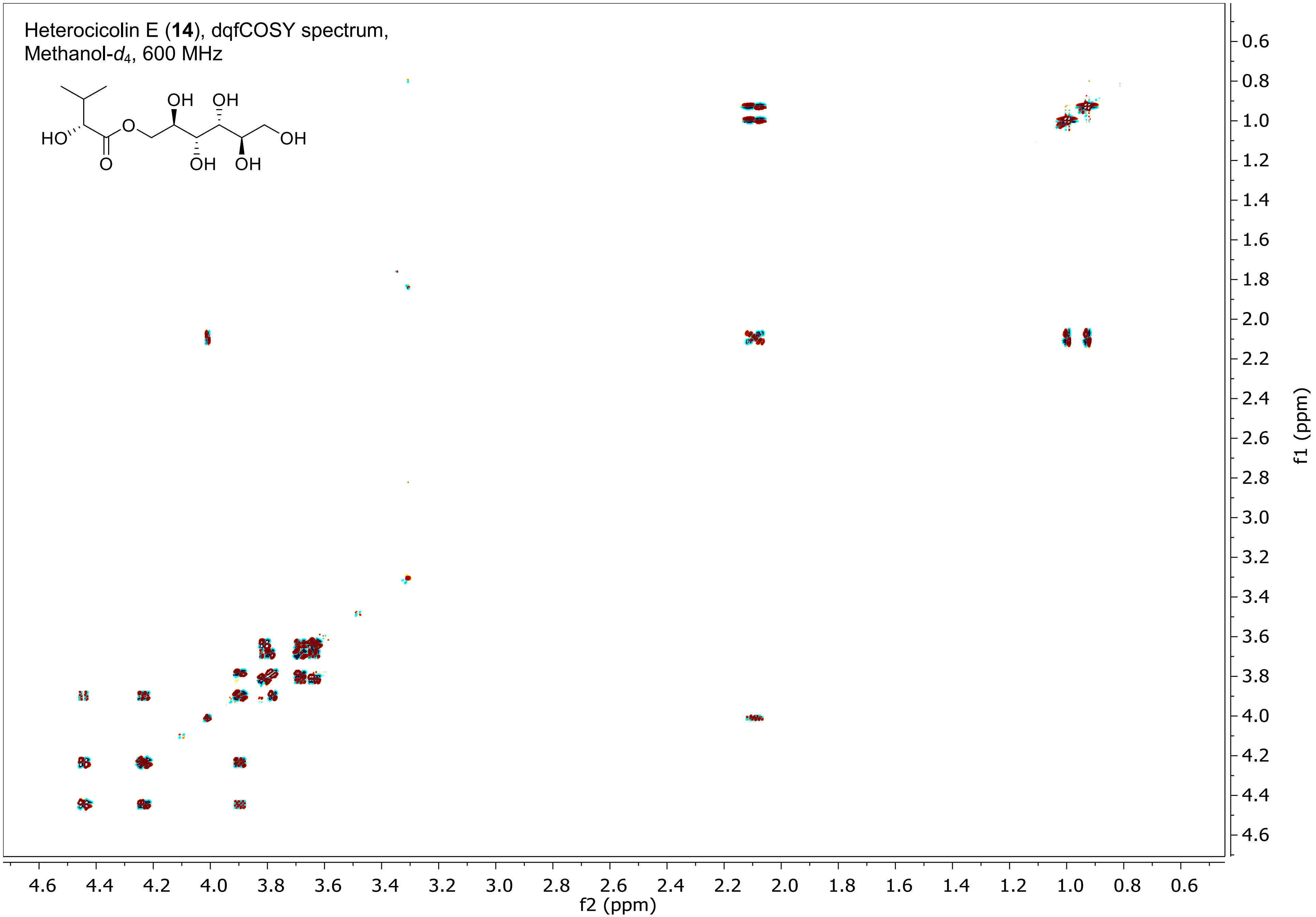
Heterocicolin E (14), dqfCOSY spectrum,
Methanol-d4, 600 MHz
HO
O
O
OH
OH
OH
OH
OH
f2 (ppm)
f1 (ppm)

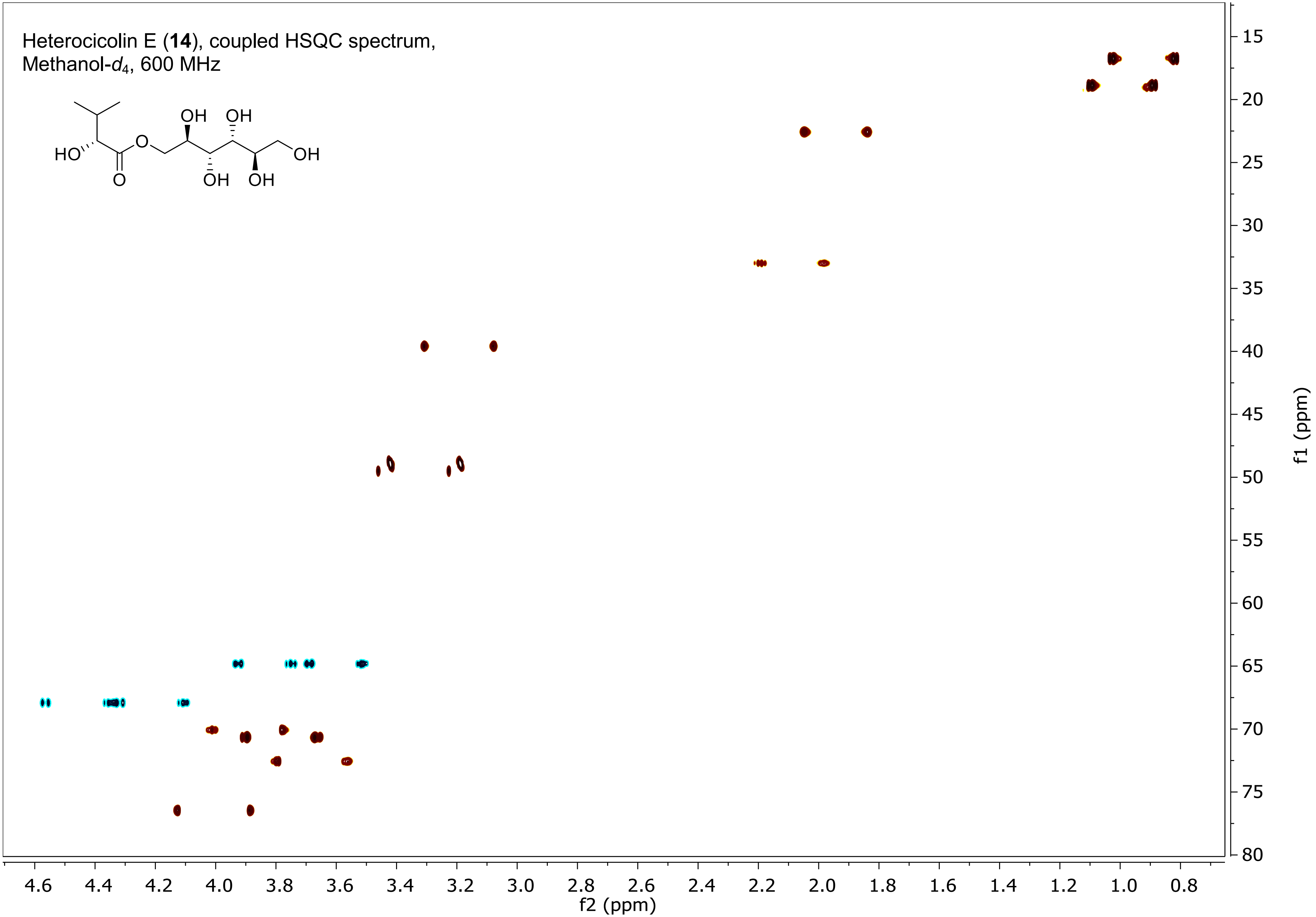

Heterocicolin E (**14**), coupled HSQC spectrum,
Methanol-d_4, 600 MHz
HO
O
O
OH
OH
OH
OH
OH
4.6
4.4
4.2
4.0
3.8
3.6
3.4
3.2
3.0
2.8
2.6
2.4
2.2
2.0
1.8
1.6
1.4
1.2
1.0
0.8
f2 (ppm)
15
20
25
30
35
40
45
50
55
60
65
70
75
80
f1 (ppm)

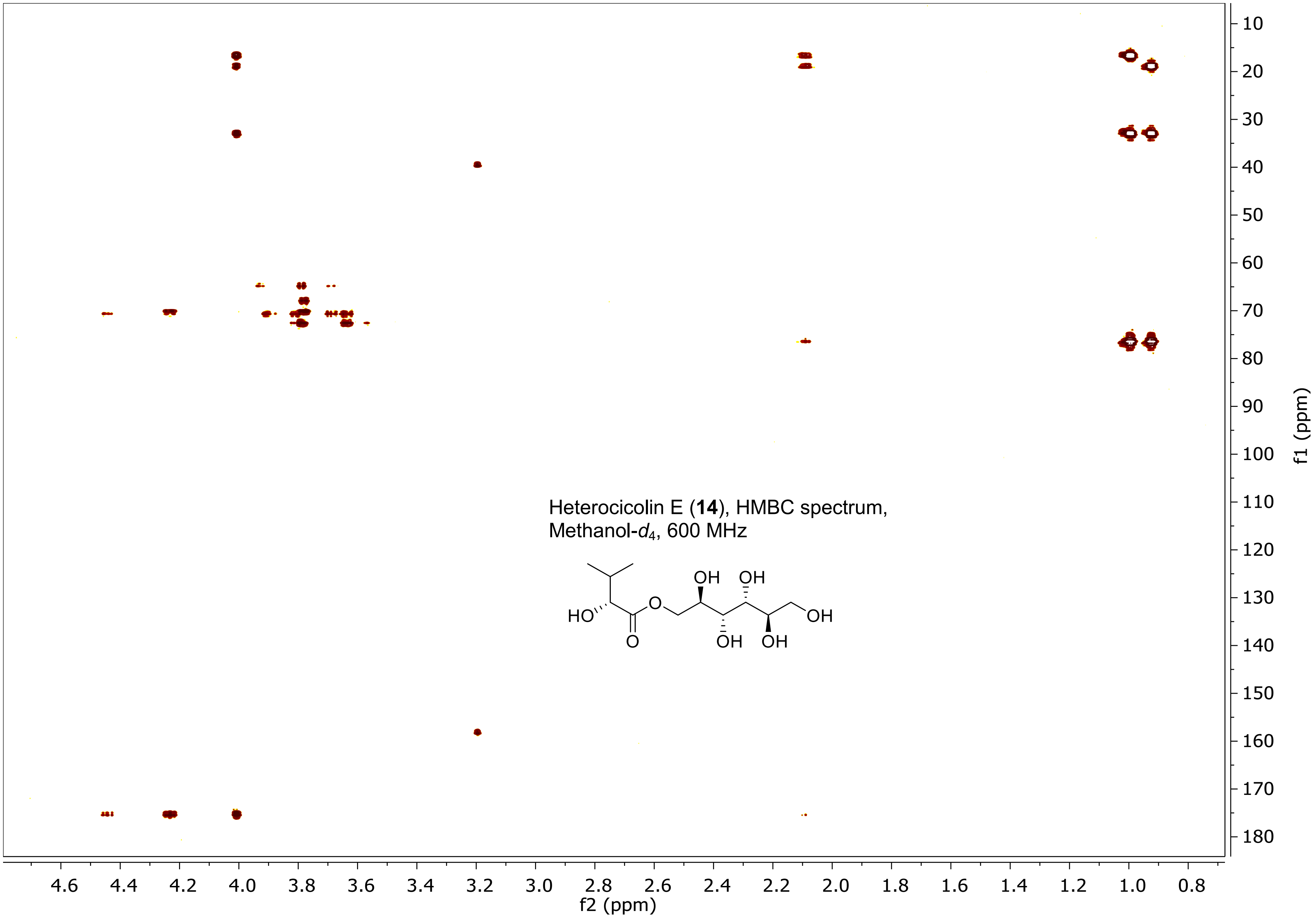
Heterocicolin E (14), HMBC spectrum,
Methanol-d_4, 600 MHz
HO
O
O
OH
OH
OH
OH
OH
f1 (ppm)
f2 (ppm)
10
20
30
40
50
60
70
80
90
100
110
120
130
140
150
160
170
180
4.6
4.4
4.2
4.0
3.8
3.6
3.4
3.2
3.0
2.8
2.6
2.4
2.2
2.0
1.8
1.6
1.4
1.2
1.0
0.8

^{1}H (600 MHz) and ^{13}C (151 MHz) NMR spectroscopic data for heterocicolin F (15) in methanol-d_4

Chemical shifts were referenced to δ(C<u>H</u>D_2OD) = 3.31 and δ(^{13}<u>C</u>HD_2OD) = 49.0. (^{1}H,^{1}H)-*J*-coupling constants were determined from the acquired ^{1}H or dqfCOSY spectra. HMBC correlations are from the proton(s) stated to the indicated ^{13}C atom.

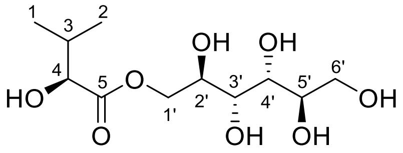

No.	δc	Proton	δH (J_{HH}[Hz])	HMBC
1	17.1	1-H	0.93 ($J_{1,3}$ = 7.0 Hz)	2, 3, 4
2	19.2	2-H	1.00 ($J_{2,3}$ = 7.0 Hz)	1, 3, 4
3	33.4	3-H	2.09 ($J_{3,1}$ = 7.0 Hz, $J_{3,2}$ = 7.0 Hz, $J_{3,4}$ = 4.6 Hz)	1, 2, 4
4	76.8	4-H	4.01 ($J_{4,3}$ = 4.6 Hz)	1, 2, 3
5	175.6			4, 1’
1’	68.3	1’-Ha	4.46 ($J_{1'a,1'b}$ = 11.5 Hz, $J_{1'a,2'}$ = 3.0 Hz)	3’
		1’-Hb	4.22 ($J_{1'b,1'a}$ = 11.5 Hz, $J_{1'b,2'}$ = 6.5 Hz)	
2’	70.5	2’-H	3.90 ($J_{2',1'a}$ = 3.0 Hz, $J_{2',1'b}$ = 6.5 Hz, $J_{2',3'}$ = 9.0 Hz)	1’
3’	71.1	3’-H	3.78 (m)	1’b, 2’, 4’, 5’
4’	71.0	1’-H	3.79 (m)	2’, 3’, 5’, 6’
5’	73.0	2’-H	3.69 ($J_{5',6'a}$ = 3.7 Hz, $J_{5',6'b}$ = 6.0 Hz, $J_{5',4'}$ = 8.5 Hz)	4’, 6b’
6’	65.2	6’-Ha	3.81 ($J_{6'a,6'b}$ = 11.2 Hz, $J_{6'a,5'}$ = 3.7 Hz)	4’, 5’
		6’-Hb	3.64 ($J_{6'b,6'a}$ = 11.2 Hz, $J_{6'b,5'}$ = 6.0 Hz)	

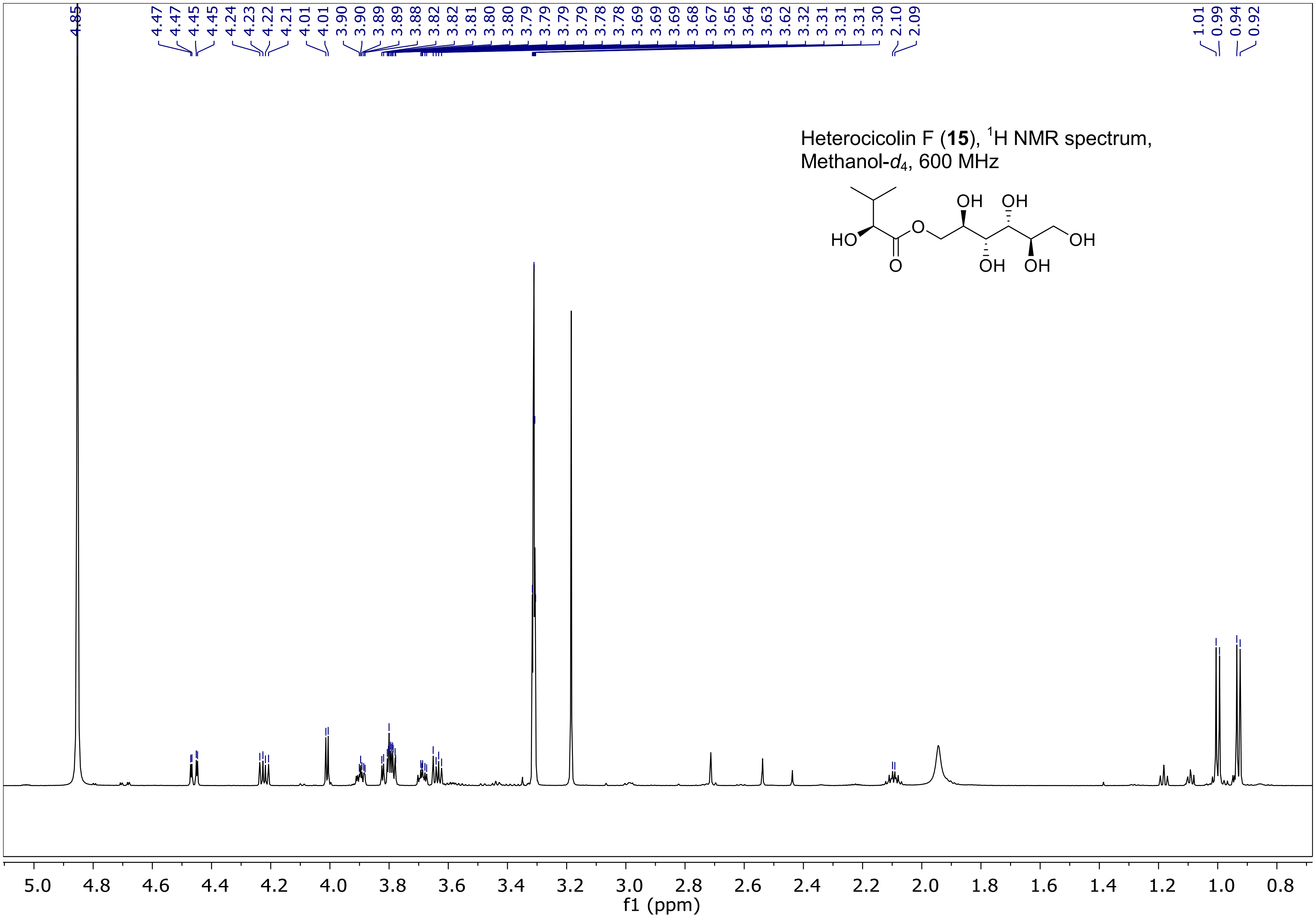
Heterocicolin F (15), ^{1}H NMR spectrum,
Methanol-d_4, 600 MHz
HO
O
O
OH
OH
OH
OH
OH
4.85
4.47
4.47
4.45
4.45
4.24
4.23
4.22
4.21
4.01
4.01
3.90
3.90
3.89
3.89
3.88
3.82
3.82
3.81
3.80
3.80
3.79
3.79
3.79
3.79
3.78
3.78
3.69
3.69
3.69
3.68
3.67
3.65
3.64
3.63
3.62
3.32
3.31
3.31
3.31
3.30
2.10
2.09
1.01
0.99
0.94
0.92
5.0
4.8
4.6
4.4
4.2
4.0
3.8
3.6
3.4
3.2
3.0
2.8
2.6
2.4
2.2
2.0
1.8
1.6
1.4
1.2
1.0
0.8
f1 (ppm)

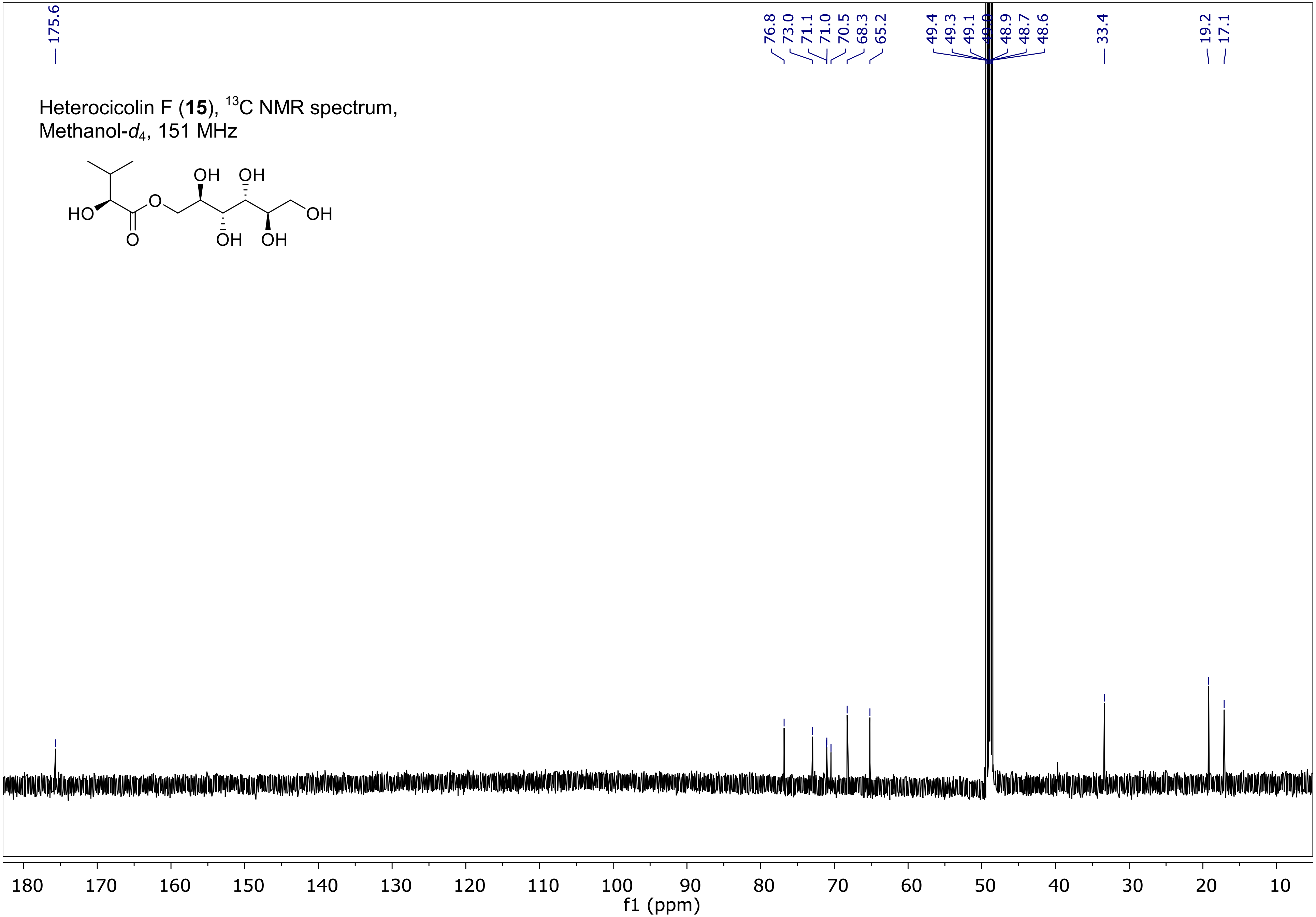

175.6
76.8
73.0
71.1
71.0
70.5
68.3
65.2
49.4
49.3
49.1
49.0
48.9
48.7
48.6
33.4
19.2
17.1
Heterocicolin F (15), 13C NMR spectrum,
Methanol-d4, 151 MHz
HO
O
O
OH
OH
OH
OH
OH
180 170 160 150 140 130 120 110 100 90 80 70 60 50 40 30 20 10
f1 (ppm)

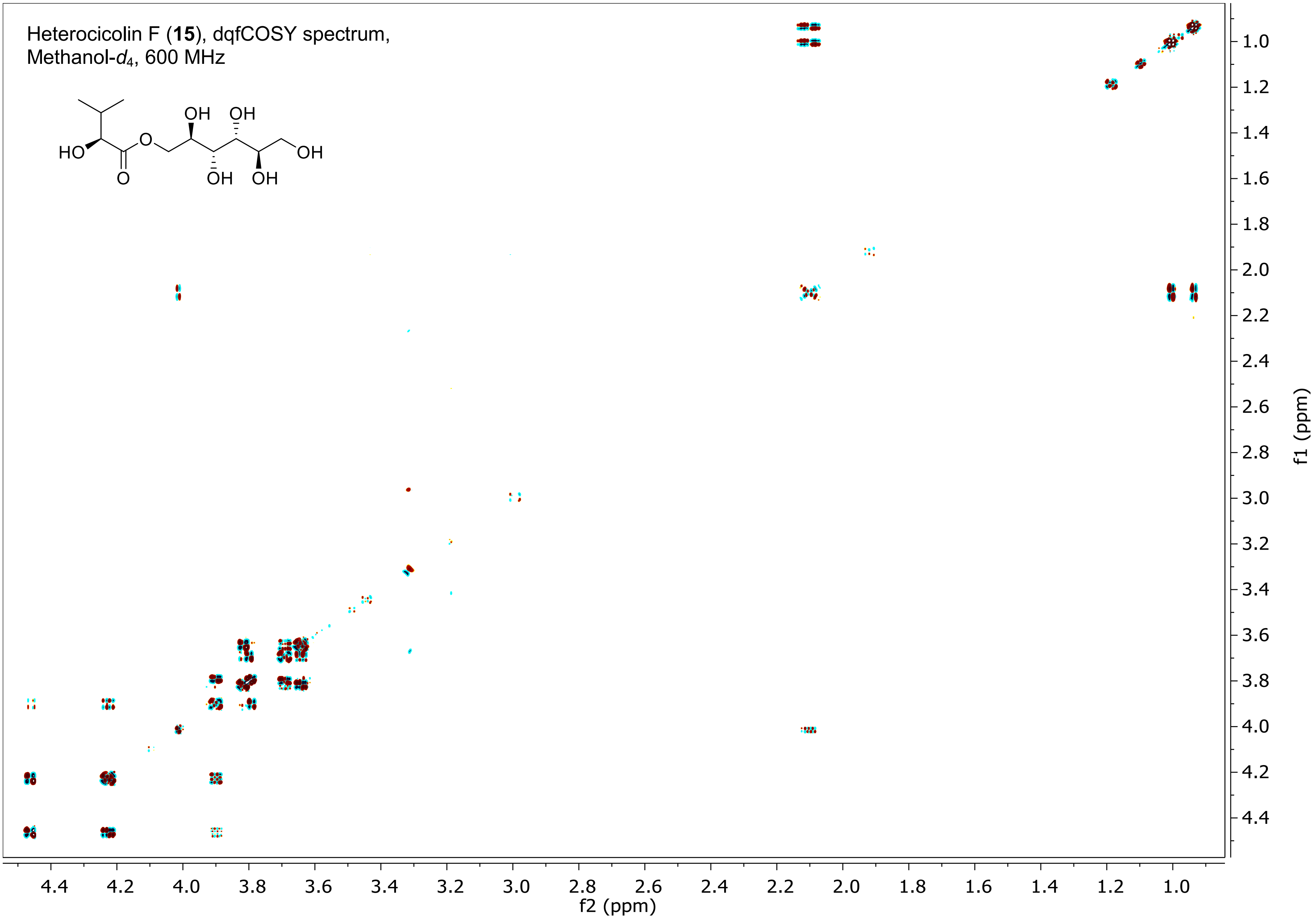
Heterocicolin F (**15**), dqfCOSY spectrum,
Methanol-d_4, 600 MHz
HO
O
O
OH
OH
OH
OH
OH
f2 (ppm)
f1 (ppm)
4.4
4.2
4.0
3.8
3.6
3.4
3.2
3.0
2.8
2.6
2.4
2.2
2.0
1.8
1.6
1.4
1.2
1.0

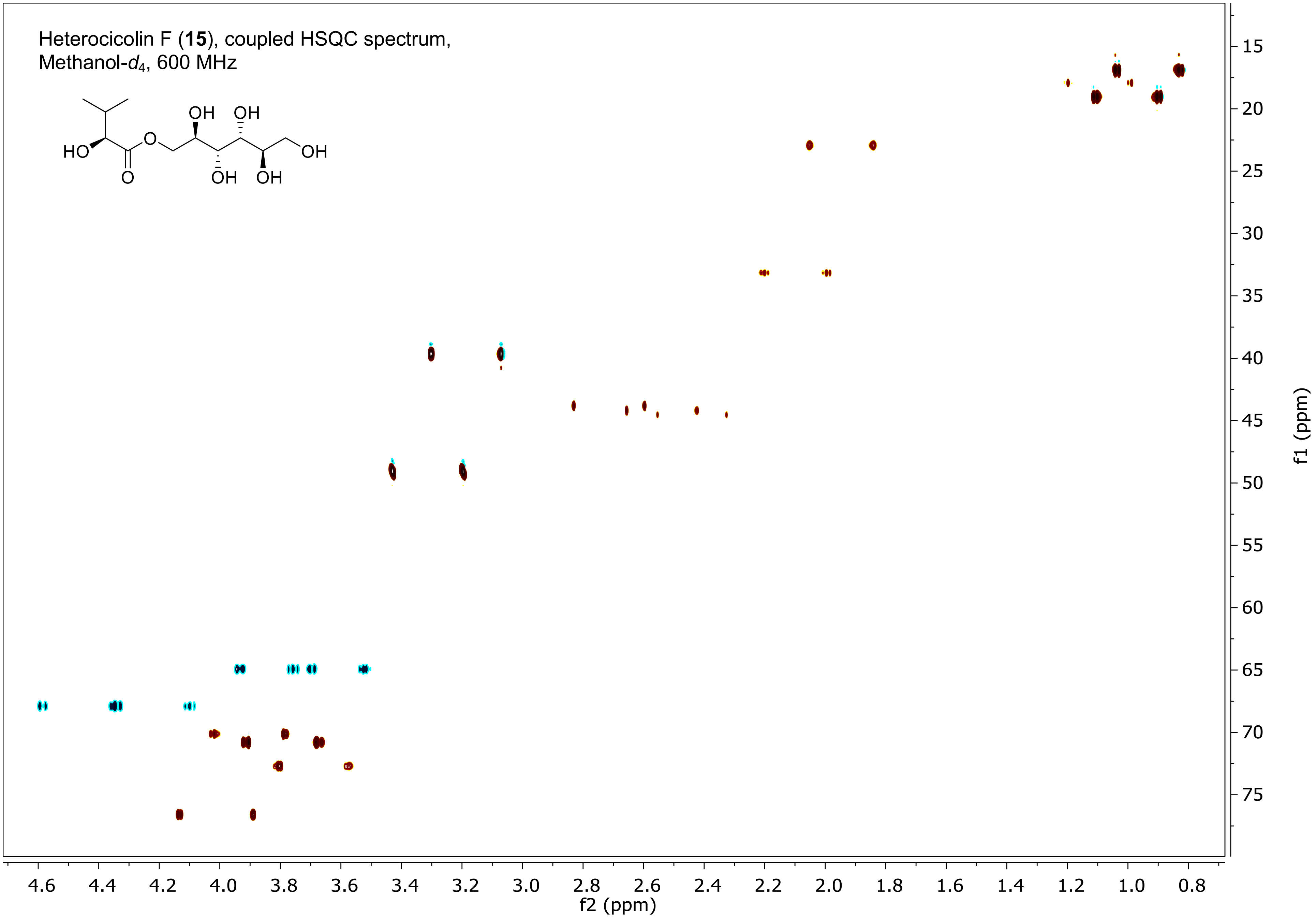
Heterocicolin F (15), coupled HSQC spectrum,
Methanol-d_4, 600 MHz
HO
O
OH
OH
OH
OH
OH
f1 (ppm)
f2 (ppm)
4.6 4.4 4.2 4.0 3.8 3.6 3.4 3.2 3.0 2.8 2.6 2.4 2.2 2.0 1.8 1.6 1.4 1.2 1.0 0.8
15 20 25 30 35 40 45 50 55 60 65 70 75

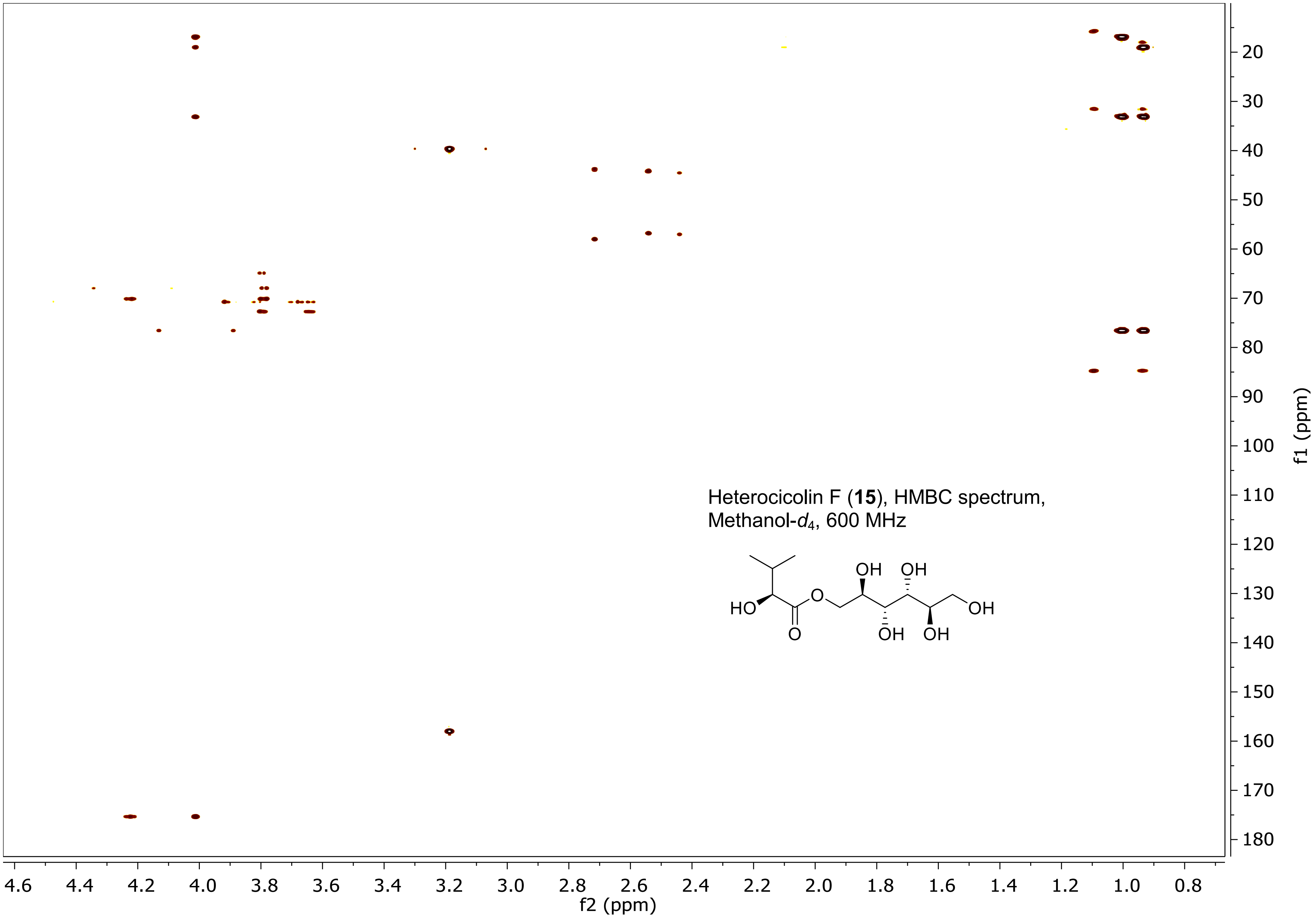
Heterocicolin F (15), HMBC spectrum,
Methanol-d4, 600 MHz
OH OH
HO
O
O
OH
OH OH
4.6 4.4 4.2 4.0 3.8 3.6 3.4 3.2 3.0 2.8 2.6 2.4 2.2 2.0 1.8 1.6 1.4 1.2 1.0 0.8
f2 (ppm)
20 30 40 50 60 70 80 90 100 110 120 130 140 150 160 170 180
f1 (ppm)

5. Supplementary References

1. Bodinaku, I. *et al.* Rapid phenotypic and metabolomic domestication of wild penicillium molds on cheese. *MBio* **10**, (2019).
2. Lim, F. Y. *et al.* Fungal isocyanide synthases and xanthocillin biosynthesis in Aspergillus fumigatus. *MBio* **9**, (2018).
3. Blachowicz, A. *et al.* Contributions of spore secondary metabolites to UV-C protection and virulence vary in different Aspergillus fumigatus strains. *MBio* **11**, 1–12 (2020).
4. Tannous, J. *et al.* New Insight Into Pathogenicity and Secondary Metabolism of the Plant Pathogen Penicillium expansum Through Deletion of the Epigenetic Reader SntB. *Front. Microbiol.* **11**, 1–13 (2020).
5. Leach, J., Lang, B. R. & Yoder, O. C. Methods for selection of mutants and in vitro culture of Cochliobolus heterostrophus. *J. Gen. Microbiol.* **128**, 1719–1729 (1982).
6. Vincent, W. J. B., Freisinger, C. M., Lam, P. Y., Huttenlocher, A. & Sauer, J. D. Macrophages mediate flagellin induced inflammasome activation and host defense in zebrafish. *Cell. Microbiol.* **18**, 591–604 (2016).
7. Wiles, T. J., Bower, J. M., Redd, M. J. & Mulvey, M. A. Use of zebrafish to probe the divergent virulence potentials and toxin requirements of extraintestinal pathogenic Escherichia coli. *PLoS Pathog.* **5**, (2009).
8. Kolter, R., Bloemberg, G. V, O'Toole, G. & Lugtenberg, B. J. Green Fluorescent Protein as a Marker for Pseudomonas spp. *Appl. Environ. Microbiol.* **63**, 4543 (1997).
