# Supplementary material for: Copper starvation induces antimicrobial isocyanide integrated into two distinct biosynthetic pathways in fungi
Source: Nat Commun. 2022 Aug 16;13:4828. doi: 10.1038/s41467-022-32394-x (PMC9381783; doi:10.1038/s41467-022-32394-x)
Supplement: Supplementary file 2 — Reporting Summary [file 41467_2022_32394_MOESM2_ESM.pdf]

nature portfolio

Corresponding author(s): Nancy P. Keller, Frank C. Schroeder

Last updated by author(s): Jul 19, 2022

Reporting Summary

Nature Portfolio wishes to improve the reproducibility of the work that we publish. This form provides structure for consistency and transparency in reporting. For further information on Nature Portfolio policies, see our Editorial Policies and the Editorial Policy Checklist.

Statistics

For all statistical analyses, confirm that the following items are present in the figure legend, table legend, main text, or Methods section.

n/a	Confirmed	
☐	☒	The exact sample size (*n*) for each experimental group/condition, given as a discrete number and unit of measurement
☐	☒	A statement on whether measurements were taken from distinct samples or whether the same sample was measured repeatedly
☐	☒	The statistical test(s) used AND whether they are one- or two-sided<br>*Only common tests should be described solely by name; describe more complex techniques in the Methods section.*
☐	☒	A description of all covariates tested
☐	☒	A description of any assumptions or corrections, such as tests of normality and adjustment for multiple comparisons
☐	☒	A full description of the statistical parameters including central tendency (e.g. means) or other basic estimates (e.g. regression coefficient) AND variation (e.g. standard deviation) or associated estimates of uncertainty (e.g. confidence intervals)
☐	☒	For null hypothesis testing, the test statistic (e.g. *F*, *t*, *r*) with confidence intervals, effect sizes, degrees of freedom and *P* value noted<br>*Give P values as exact values whenever suitable.*
☒	☐	For Bayesian analysis, information on the choice of priors and Markov chain Monte Carlo settings
☒	☐	For hierarchical and complex designs, identification of the appropriate level for tests and full reporting of outcomes
☒	☐	Estimates of effect sizes (e.g. Cohen's *d*, Pearson's *r*), indicating how they were calculated

Our web collection on statistics for biologists contains articles on many of the points above.

Software and code

Policy information about availability of computer code

Data collection	LC-MS data were collected using Thermo Scientific Xcalibur software version 4.1.31.9.
Data analysis	LC-MS data were analyzed using Metaboseek software version 0.9.7 and quantification was performed via integration in Excalibur Quan Browser version 4.1.31.9. All statistical analysis were performed with GraphPad Prism version 9.2 or Metaboseek version 0.9.7. NMR spectra were processed and baseline corrected using MestreLabs MNOVA software packages version 11.0.0-17609.

For manuscripts utilizing custom algorithms or software that are central to the research but not yet described in published literature, software must be made available to editors and reviewers. We strongly encourage code deposition in a community repository (e.g. GitHub). See the Nature Portfolio guidelines for submitting code & software for further information.

Data

Policy information about availability of data

All manuscripts must include a data availability statement. This statement should provide the following information, where applicable:

- Accession codes, unique identifiers, or web links for publicly available datasets
- A description of any restrictions on data availability
- For clinical datasets or third party data, please ensure that the statement adheres to our policy

Source data are provided with this paper. MS and MS/MS data for all fungal metabolome samples analyzed in this study (A. fumigatus, P. commune, P. expansum, C. heterostrophus, co-culture of A. fumigatus and P. expansum, and deuterium labeling experiments) have been uploaded to the GNPS Web site (massive.ucsd.edu). MassIVE ID number is MSV000089206 [https://massive.ucsd.edu/ProteoSAFe/dataset.jsp?accession=MSV000089206].
The crmA sequence data used in this study are available in the NCBI database under accession code XP_754255 [https://www.ncbi.nlm.nih.gov/protein/XP_754255] for A. fumigatus, XP_016596276 [https://www.ncbi.nlm.nih.gov/protein/XP_016596276.1] for P. expansum, and EMD96666 [https://www.ncbi.nlm.nih.gov/protein/

EMD96666] for C. heterostrophus.
The sequence data used in this study are available in the NCBI database under accession code GCF_000002655.1 [https://www.ncbi.nlm.nih.gov/assembly/GCF_000002655.1] for A. fumigatus, GCA_008931945.1 [https://www.ncbi.nlm.nih.gov/assembly/GCA_008931945.1] for P. commune, GCA_000338975.1 [https://www.ncbi.nlm.nih.gov/assembly/GCA_000338975.1] for C. heterostrophus. The sequence data for A. brassicicola is available in the JGI Mycocosm database under accession code ATCC96836 [https://mycocosm.jgi.doe.gov/Albra1/Albra1.home.html].

Field-specific reporting

Please select the one below that is the best fit for your research. If you are not sure, read the appropriate sections before making your selection.

[x] Life sciences   [ ] Behavioural & social sciences   [ ] Ecological, evolutionary & environmental sciences

For a reference copy of the document with all sections, see nature.com/documents/nr-reporting-summary-flat.pdf

Life sciences study design

All studies must disclose on these points even when the disclosure is negative.

Sample size	No sample size calculations were performed. Sample sizes were selected based on reasonable replication and prior experience with similar assays.<br>For liquid fungal cultures, our independent biological experiments with sample sizes (n=3 to 6) for each deletion and overexpression mutants of crmA and wild type of fungal species revealed reproducible production or abolition of the metabolites with statistical significance, which provided conclusive evidence of sufficient sample sizes as to whether metabolites were produced via a specific gene.<br>Similarly, sample numbers for measurements of anti-microbial effects (n=3 to 6) provided conclusive evidence.
Data exclusions	No were data excluded for any of the experiments in the manuscripts.
Replication	Generally, experiments were independently replicated at least three times, except in specific cases, e.g. for PCR analyses and Southern blots, as specified in Methods.
Randomization	Not applicable. Samples were allocated into experimental groups based on genotype and supplement. For liquid fungal cultures, wild type and dmaW and crmA mutants of Aspergillus fumigatus were grown in parallel under reference conditions. Wild type and crmA mutants of other fungal species, Penicillium expansum, P. commune, and Cochliobolus heterostrophus, were grown in parallel under reference conditions.<br>Similarly, anti-microbial effects were measured in parallel under reference conditions.
Blinding	Blinding is not possible since mutants have different properties.

Reporting for specific materials, systems and methods

We require information from authors about some types of materials, experimental systems and methods used in many studies. Here, indicate whether each material, system or method listed is relevant to your study. If you are not sure if a list item applies to your research, read the appropriate section before selecting a response.

Materials & experimental systems

n/a	Involved in the study	
[x]	[ ]	Antibodies
[x]	[ ]	Eukaryotic cell lines
[x]	[ ]	Palaeontology and archaeology
[x]	[ ]	Animals and other organisms
[x]	[ ]	Human research participants
[x]	[ ]	Clinical data
[x]	[ ]	Dual use research of concern

Methods

n/a	Involved in the study	
[x]	[ ]	ChIP-seq
[x]	[ ]	Flow cytometry
[x]	[ ]	MRI-based neuroimaging
