# Supplementary material for: Copper starvation induces antimicrobial isocyanide integrated into two distinct biosynthetic pathways in fungi
Source: Nat Commun. 2022 Aug 16;13:4828. doi: 10.1038/s41467-022-32394-x (PMC9381783; doi:10.1038/s41467-022-32394-x)
Supplement: Supplementary file 3 — Description of Additional Supplementary Files [file 41467_2022_32394_MOESM3_ESM.pdf]

File Name: Supplementary Data 1
Description: Fungal taxa containing crmA homologs.

File Name: Supplementary Data 2
Description: MS features whose production was abolished or strongly downregulated (>20 fold) in the crmA deletion mutant compared to WT or the crmA overexpression mutant under copper-limited conditions. All of these crmA-dependent features showed no significant change (0.05) in crmBC and crmCD deletion mutants compared to WT calculated by Metaboseek. Additional manually annotated columns include tentative compound class assignment.
